# Unusually high α-proton acidity of prolyl residues in cyclic peptides†

**DOI:** 10.1039/d0sc02508a

**Published:** 2020-07-02

**Authors:** Oliver R. Maguire, Bethany Taylor, Eleanor M. Higgins, Matthew Rees, Steven L. Cobb, Nigel S. Simpkins, Christopher J. Hayes, AnnMarie C. O'Donoghue

**Affiliations:** Department of Chemistry, Durham University South Road Durham DH1 3LE UK annmarie.odonoghue@durham.ac.uk; School of Chemistry, University of Birmingham Edgbaston Birmingham B15 2TT UK; School of Chemistry, University of Nottingham, University Park Nottingham NG7 2RD UK

## Abstract

The acidity of the α-proton in peptides has an essential role in numerous biochemical reactions and underpins their stereochemical integrity, which is critical to their biological function. We report a detailed kinetic and computational study of the acidity of the α-proton in two cyclic peptide systems: diketopiperazine (DKP) and triketopiperazine (TKP). The kinetic acidity (protofugality) of the α-protons were determined though hydrogen deuterium exchange studies in aqueous solutions. The acidities of the α-proton in prolyl residues were increased by 3–89 fold relative to other amino acid residues (prolyl > glycyl ≫ alanyl > tyrosyl). Experimental and computational evidence for the stereoelectronic origins of this enhanced prolyl reactivity is presented. TKPs were 10^6^-fold more reactive than their DKP analogues towards deprotonation, which we attribute to the advanced development of aromaticity in the earlier transition state for proton transfer in these cases. A Brønsted linear free energy analysis of the reaction data was conducted to provide estimates of α-proton p*K*_a_s.

## Abstract 2

Kinetic and computational studies reveal that prolyl residues in cyclic peptides are substantially more acidic than other residues due to a stereoelectronic effect.

## Graphical abstract

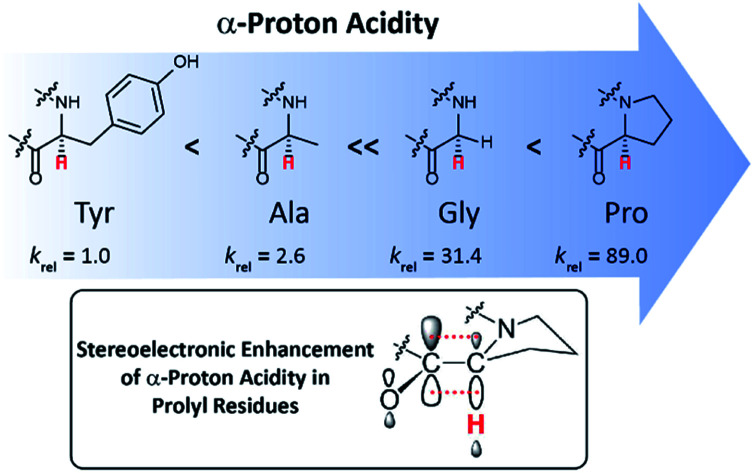

## Introduction

Proline is unique amongst the proteinogenic amino acids in its ability to induce structural and conformational modifications in proteins.^1^ These unique characteristics are often linked to proline being the only proteinogenic amino acid with a secondary N-terminal amino functionality. The resulting effect this has upon conformations of (a) the *cis*–*trans* isomers of the prolyl amide bond, and, (b) the *endo*/*exo* ring pucker of the pyrrolidine ring of proline, which links nitrogen to the α-carbon position, combine together to play a crucial role in the establishment of the correct secondary structure during protein folding.^2,3^ The rate of Xaa-Pro *cis*/*trans* isomerism has been shown to be a rate-limiting step in the folding of proteins.^4,5^ Additionally, the proliferation of prolyl residues in the enzymes of thermophilic organisms has been linked to enhanced protein stabilities in more extreme host environments.^6,7^

Many of the contributions that prolyl residues make to protein structure and stability are underpinned by stereoelectronic effects. The *gauche* effect from substitution of the 4-position plays an important role in the *endo*/*exo* ring pucker of the pyrrolidine ring which affects protein structure.^2,3,8,9^ The cumulative effects of n-to-π* interactions from proline and 4-hydroxyproline residues have been shown to contribute significantly to protein stability, as exemplified in the case of collagen.^2,10,11^

The unique chemistry of proline is not confined to influences on protein structures and stabilities. Proline and small-molecule derivatives have been widely shown to be efficient, stereoselective catalysts for a range of (bio)organic transformations.^12–17^ The superior abilities of proline derivatives as organocatalysts compared with other amino acids is often ascribed to the increased nucleophilicity of the prolyl secondary amine and influence on *cis*/*trans* isomerism in enamine intermediates.^18–24^ Mayr and co-workers have demonstrated a 100-fold increased nucleophilicity of the secondary amino group of proline towards reaction with diarylcarbenium ions relative to the primary amino groups of other amino acids.^25^ Myers and Raines recently reported a detailed kinetic study of the hydrogen–deuterium exchange reactions of cyclohexanone catalysed by proline derivatives in aqueous solution. Their kinetic structure–activity analysis demonstrated that inter- and intramolecular electrostatic interactions involving charged and electron-rich atoms derived from the proline catalyst, cyclohexanone substrate and buffer can have dramatic influences on catalytic activity.^26^ Moreover, Xaa-Pro bonds are a highly conserved structural motif for catalytically active peptides^27–32^ for which *cis*/*trans* isomerization has been directly linked to the stereoselectivity of the peptide catalyst.^33^ Finally, the enhanced catalytic ability of proline has broader implications in the origin of chirality in prebiotic chemistry.^34–41^ For example, Blackmond and co-workers reported that l-proline alkyl ammonium salts can induce the formation of an enantiomeric excess of d-sugars in the formose reaction.^42^

**Fig. 1:**
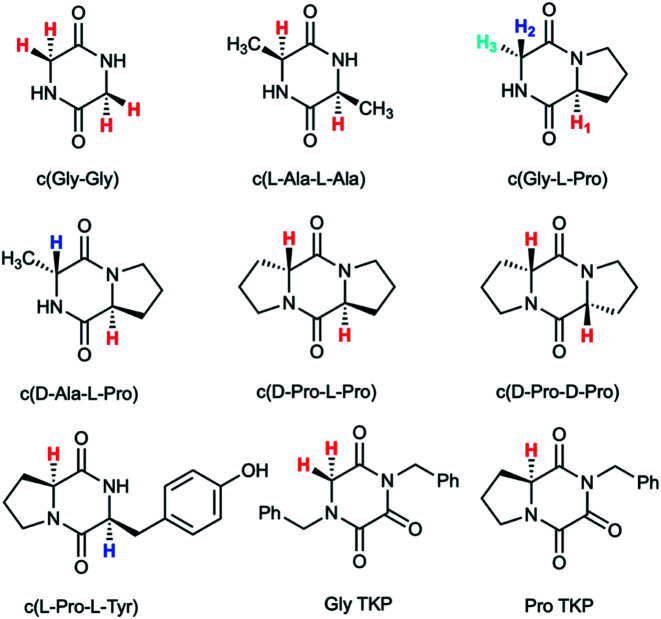
Diketopiperazines (DKPs) and triketopiperazines (TKPs) used in our hydrogen–deuterium exchange study of α-protons. The exchangeable protons of interest are highlighted.

Herein we report an additional unique property of proline in the substantial enhancement of the acidity of its α-proton relative to other amino acids in cyclic peptide systems. As the simplest examples of cyclic peptides, we have chosen 2,5-diketopiperazine (DKP) and triketopiperazine (TKP) systems (Fig. 1). We undertook a series of hydrogen–deuterium exchange studies to determine the kinetic lability, or protofugality,^43^ of the α-protons in these DKPs and TKPs. Second order rate constants for base-catalysed exchange were observed to be substantially higher for prolyl containing DKPs and TKPs. Furthermore, the TKPs were orders of magnitude more kinetically labile towards deprotonation than DKPs. Rate constants for deprotonation could be correlated using the Brønsted linear free energy relationship to shed light on the enhanced acidities (protofugalities) in these cyclic peptide systems and to provide estimates of α-carbon acid p*K*_a_s. Electronic structure calculations replicated experimental trends and led to further insight into the stereochemical origins of the enhanced lability of the prolyl α-protons in these DKPs and the role of aromaticity in the deprotonation of TKP α-protons. These data provide new insight into the fundamental properties of prolyl-containing cyclic peptide systems to inform synthetic and biological applications for which the stereochemical integrity of peptide derivatives, both in solution and *in vivo*, is crucial.^44–46^

## Results and discussion

### Deuterium exchange of diketopiperazines (DKPs)

**Fig. 2:**
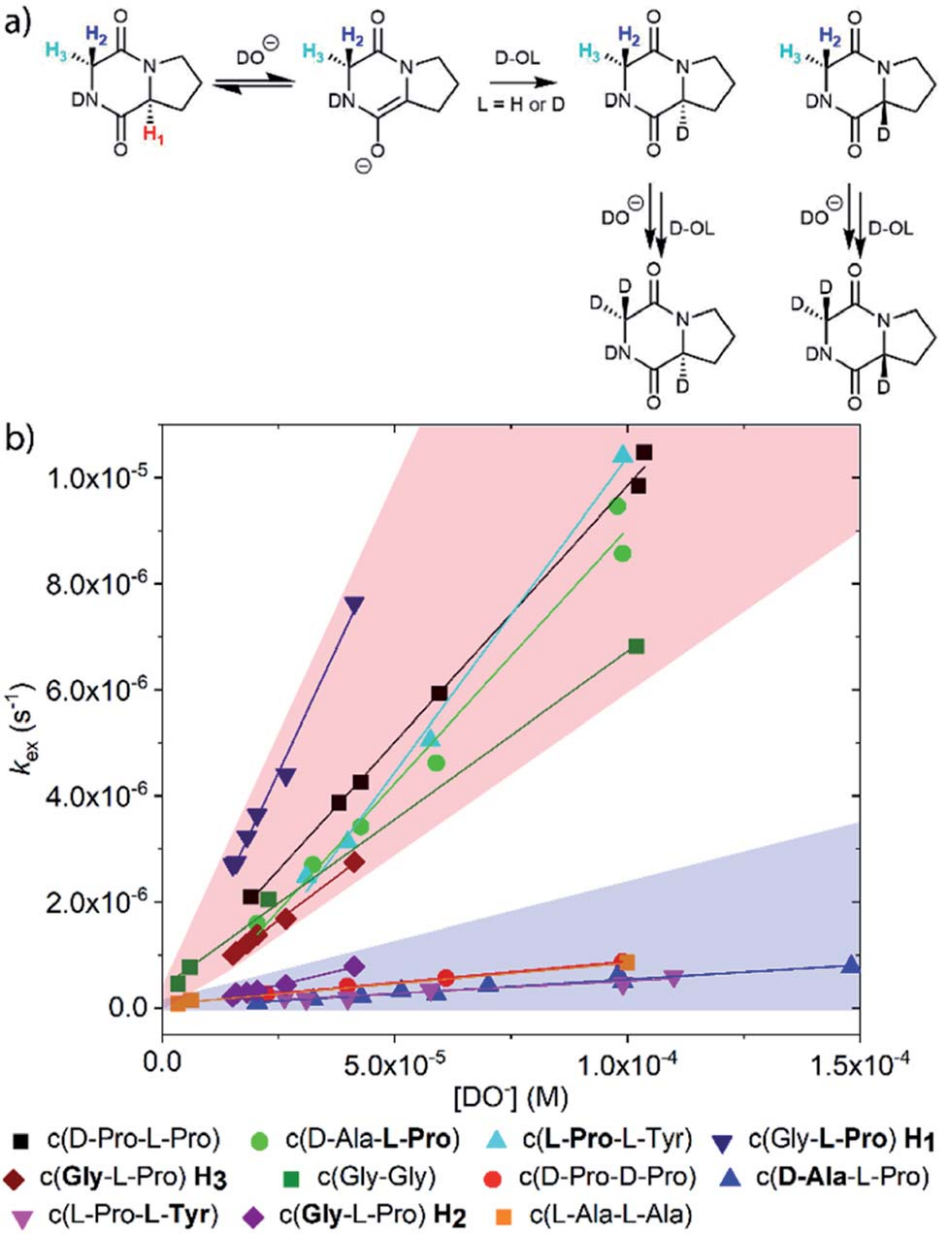
(a) Proposed mechanism of hydrogen–deuterium exchange *via* enolate formation shown for Gly–L–Pro; (b) dependence of the observed rate constant of H/D exchange, *k*_ex_, upon the concentration of deuteroxide for the DKPs studied at 25 °C and *I* = 1.0 (KCl). The red region encompasses the kinetic data for the majority of prolyl and glycyl residues whereas the blue region spans the data for all other amino acid residues. The slopes of linear fits of the H/D-exchange kinetic data are the second order rate constants for deuteroxide catalysed exchange, *k*_DO_ (M^−1^ s^−1^).

DKPs are small cyclic dipeptides found in many natural products and are also common by-products in solid phase peptide synthesis.^44^ Owing to their prevalence in pharmaceutically important compounds and their non-planar structure, DKPs have acquired the status of privileged structures in medicinal chemistry.^44,47^ We determined the reactivity towards deprotonation of the α-protons for a range of 2,5-DKPs in Fig. 1, which were prepared readily by cyclisation of appropriate protected linear peptides or were commercially available (ESI†). The reactions were analysed by ^1^H NMR spectroscopy in carbonate or 3-chloroquinuclidine buffered D_2_O solutions in the p*D* range 9.35–10.94 at 25 °C and ionic strength *I* = 1.0 (KCl). Outside this p*D* range, the exchange reactions were either too fast or slow for NMR kinetic analysis. The unambiguous observation of hydrogen–deuterium exchange supports a mechanism involving enolate formation (Fig. 2a), where exchange is accompanied by epimerization (see ESI† for details of kinetic evaluation). Fig. 2b shows the dependence of the observed first order rate constants of exchange, *k*_ex_ (s^−1^), upon deuteroxide concentration for the DKPs. A clear first order dependence on deuteroxide concentration is observed, however, no significant additional general base catalysis of exchange was detectable. The slopes of linear fits of the H/D-exchange kinetic data are the second order rate constants for deuteroxide catalysed exchange, *k*_DO_ (M^−1^ s^−1^) (Table 1).

**Table 1:** Values for *k*_DO_, *k*_HO_, and p*K*_a_s of the DKPs studied at 25 °C and *I* = 1.0 M (KCl)

	*k*_DO_ (M^−1^ s^−1^)	*k*_HO_ b (M^−1^ s^−1^)	*k*_rel_ c	p*K*_a_^d^
c(Gly–Gly)	6.33 × 10^−2^	3.17 × 10^−2^	12.2	20.9
c(l-Ala–l-Ala)	7.80 × 10^−3^	3.90 × 10^−3^	3.00	22.7
c(Gly–**l-Pro**)	1.87 × 10^−1^	9.35 × 10^−2^	144	18.8
c(**H2-Gly**–l-Pro)	2.09 × 10^−2^	1.05 × 10^−2^	16.2	21.2
c(**H3-Gly**–l-Pro)	6.59 × 10^−2^	3.30 × 10^−2^	50.8	19.9
c(d-Ala–**l-Pro**)	9.45 × 10^−2^	4.73 × 10^−2^	72.8	19.6
c(**d-Ala**–l-Pro)	5.51 × 10^−3^	2.76 × 10^−3^	4.25	22.6
c(d-Pro–l-Pro)	9.69 × 10^−2^	4.85 × 10^−2^	37.3	20.0
c(d-Pro–d-Pro)	8.62 × 10^−3^	4.31 × 10^−3^	3.30	22.6
c(**l-Pro**–l-Tyr)	1.19 × 10^−1^	5.95 × 10^−2^	91.5	19.3
c(l-Pro–**l-Tyr**)	2.09 × 10^−3^	1.05 × 10^−3^	1.60	23.7
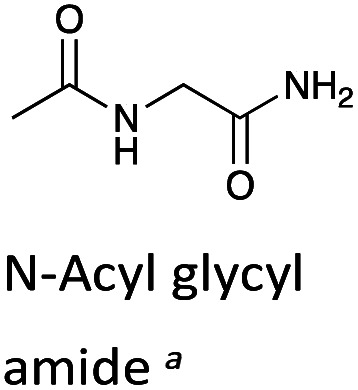	2.6 × 10^−3^	1.3 × 10^−3^	1.00	23.9

a H/D-exchange kinetic data for *N*-acyl glycyl amide, a linear analogue of a DKP, is included for comparison.^48^

b Calculated from experimental *k*_DO_ values using a secondary solvent isotope effect *k*_DO_/*k*_HO_ = 2.00 (see text).

c *k*_rel_ = relative rate constants for deprotonation compared to value for linear *N*-acyl glycyl amide as reference. The *k*_rel_ values have been statistically corrected for the number of exchangeable protons.

d Interpolated values from a Brønsted analysis (see text).

The comparison of reactivities towards deprotonation by a common base, DO^−^, allows for the determination of a DKP Brønsted kinetic acidity scale (or protofugality scale).^43^ The observed kinetic acidity (protofugalities) (*k*_DO_) of the DKP amino acid residues fall into two broad regions with Pro and Gly (red region) being substantially more kinetically acidic than Ala and Tyr (blue region) according to the following acidity trend:Pro > Gly ≫ Ala > Tyr

The Gly and Ala residues follow expected trends with Ala being less acidic than Gly due to inductive destabilisation by the electron donating methyl group of the carbanion/enolate formed upon deprotonation by DO^−^. The lower reactivity of Tyr could be due to the significant geometric rearrangement required upon enolate formation, which raises the barrier to deprotonation. The phenol(ate) ring of Tyr prefers a folded conformation over the DKP ring in the keto form.^49^ The change from sp^3^ to sp^2^ hybridisation upon enolate formation would enforce a more open conformation of the phenol(ate) ring with greater solvent exposure and an associated entropic penalty.^50^

**Fig. 3:**
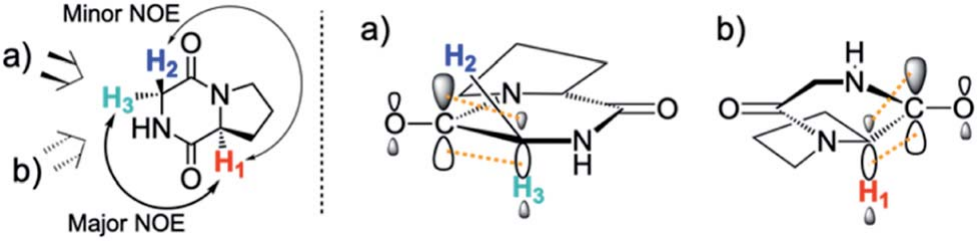
Proposed stereoelectronic effect to account for the difference in acidities between the glycyl α-protons in c(Gly–l-Pro) and the enhanced acidity of the prolyl α-proton. The structure on the left indicates the line of sight for (a) glycyl α-protons, and (b) prolyl α-proton. The larger NOE interaction between H_1_ and H_3_ and the smaller NOE interaction between H_1_ and H_2_ are shown. Proposed stereoelectronic overlap between σ_C–H_ and 
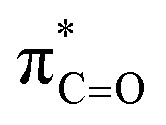
 indicated by dashed orange lines.

By contrast, the prolyl residues were more acidic than all other DKP residues with *k*_DO_ from 3–89-fold larger (apart from c(d-Pro–d-Pro)). A stereoelectronic effect is proposed as the major origin of the enhanced rates of deprotonation (higher protofugalities) of the Pro C_α_-protons in DKPs. Evidence for the contribution of a stereoelectronic effect initially came from the two glycyl α-protons H_2_ and H_3_ in c(Gly–l-Pro), which were found to have a 3.1-fold difference in reactivity towards DO^−^ (H_2_: *k*_DO_ = 2.09 × 10^−2^ M^−1^ s^−1^; H_3_: *k*_DO_ = 6.59 × 10^−2^ M^−1^ s^−1^). The higher ^1^H NMR chemical shift of the glycyl H_3_ proton (4.03 ppm) relative to the H_2_ proton (3.74 ppm) suggests a greater elongation of the C–H_3_ bond possibly as a result of an enhanced σ_C–H_ to 
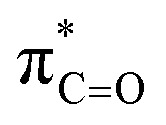
 interaction (Fig. 3a).

This stereoelectronic effect can also be present for the Pro α-proton in c(Gly–l-Pro) (H_1_, Fig. 3b) to explain the substantially increased reactivity towards deprotonation. The additional conformational restrictions imposed by the pyrrolidine ring predisposes Pro residues towards an optimal stereoelectronic alignment of the σ_C–H_ and 
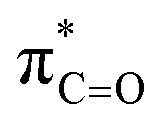
 orbitals for deprotonation of the Pro α-proton. The NOESY spectrum of c(Gly–l-Pro) in Fig. S1.†^36^ Shows the glycyl H_3_ α-proton at 4.03 ppm has a larger coefficient of interaction with the prolyl H_1_ α-proton at 4.17 ppm than the glycyl H_2_ α-proton at 3.74 ppm. This suggests that H_1_ in c(Gly–l-Pro) lies on the same face of the DKP as H_3_, which similarly enhances the orbital overlap between the σ_C–H_ and 
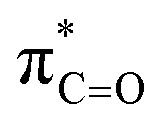
, lowering the barrier to enolate formation.

To gain further insight into the stereoelectronic effect we performed electronic calculations with c(Gly–l-Pro) at the BMK/6-31+g(d) level. An NBO analysis shows that the computed C–H bond lengths increased in the order C–H_2_ < C–H_3_ < C–H_1_ which is in agreement with the observed lability order of C–H_1_ > C–H_3_ > C–H_2_ (Table 2). The calculated structures confirmed that the prolyl H_1_ and glycyl H_3_ are located on the same face of the DKP ring. The computed structures and energies of enolates resulting from DKP deprotonation also corroborate the deuterium exchange kinetic data. The Pro-derived enolate is 2.6 kcal mol^−1^ more stable than the Gly-enolate formed from c(Gly–l-Pro).

**Table 2:** NBO analysis of the C–H bonds in c(Gly–l-Pro) at the BMK/6-31+g(d) level

c(Gly–l-Pro) Proton	Bond order	Electron occupancy of 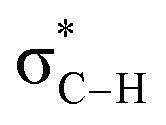	C–H Bond length (Å)
C–H_1_	0.961	0.02580	1.10573
C–H_2_	0.986	0.00812	1.0936
C–H_3_	0.976	0.02115	1.10375

Larger *k*_DO_ values are also observed for c(d-Ala–**l-Pro**), c(d-Pro–l-Pro) and c(**l-Pro**–l-Tyr). The absence of such an effect in c(d-Pro–d-Pro) can be attributed to the inter-dependent conformational preferences between the two pyrrolidine and DKP rings in the Pro–Pro case.^51^ Fig. S3.1† compares the starting DKPs and transition state structures for deprotonation of both c(d-Pro–l-Pro) and c(d-Pro–d-Pro) by LiOH using a solvation model for water at the BMK/6-31+g(d) level. The energies of the two transition states are similar. The main difference in activation barriers comes from the difference in energies of the two DKP starting materials with the central DKP ring of c(d-Pro–d-Pro) adopting a boat conformation and the pyrrolidine ring a more stable envelope conformation. By contrast, the planar DKP ring of c(d-Pro–l-Pro) enforces a less-stable half-chair pyrrolidine conformation increasing the energy of the reactant.

To our knowledge, there has been no reported kinetic study of the H/D exchange reactions of the α-protons of DKPs in biologically-relevant aqueous solution, although the acidity of the amide protons have been studied.^52^ Coote and Easton have examined the H/D exchange reactions of Gly, Ala and Leu-derived DKPs in non-aqueous d_6_-acetone with DBU as base at 50 °C.^53^ Their results indicated that N-substitution had a greater effect upon the acidity of the remote, as opposed to the adjacent, α-protons in the DKP ring.^54^ In our study the additional N-substitution derived from the pyrrolidine ring of Pro does not reduce the acidity of the remote α-protons of glycine and alanine residues relative to c(Gly–Gly) and c(l-Ala–l-Ala), respectively. However, as prolyl residues were not studied by Coote and Easton, a direct comparison is not possible.

### Cyclisation effects on proton transfer

To further assess the effect of cyclisation, we can compare our data for the DKPs with analogous data for linear peptide derivatives. Richard and co-workers have reported extensive, detailed studies of the H/D-exchange reactions of non-cyclic amino acids and peptides in aqueous solution.^48,55–60^ We have chosen to compare our data with their reported *k*_DO_ value^48^ for the Gly α-protons of *N*-acyl glycyl amide as a reference (*k*_rel_, Table 1), owing to its overall neutrality and equivalent chain atoms to the central DKP ring. With the exception of the Tyr residue of c(l-Pro–l-Tyr), all other amino acid residues show a significant increase in kinetic acidity (protofugality) upon cyclisation to a DKP.

In particular, it can be calculated that the prolyl α-proton in a DKP experiences the largest increase in *k*_DO_ compared to the linear system by over two orders of magnitude (*k*_rel_ = 144). Richard and co-workers reported near identical *k*_DO_ values (∼4.5 × 10^−5^ M^−1^ s^−1^) for the amino acids glycine and proline showing the lack of any significant stereoelectronic effects prior to cyclisation.^60^ Our new data highlights that cyclisation of a peptide substantially increases the labilities of the α-protons, which can be further enhanced by stereoelectronic factors especially in the case of Pro residues.

### Deuterium exchange of triketopiperazines (TKPs)

To further explore the enhanced acidity of the prolyl α-protons we also undertook deuterium exchange studies of the α-protons in two TKP systems (Fig. 1). TKPs are masked amino acids of similar structure to DKPs but with a carbonyl at the C2-position instead of a second sp^3^ α-carbon (Fig. 1).^61^ TKPs have recently been demonstrated to be excellent substrates in organocatalytic asymmetric Michael addition reactions. This has enabled highly enantioselective access to chiral TKPs and DKPs,^62^ α-CF_3_ amides,^63^ bicyclo[2.2.2]diazaoctanes related to the prenylated alkaloid family,^64^ prolinamides and 2,7-diazabicyclo[2.2.1]heptanes.^65^ To the best of our knowledge there has been no reported H/D-exchange reaction of TKPs in any solvent.

The H/D-exchange reactions of the TKPs were six orders of magnitude faster than observed for all the DKPs. At p*D* values above 6, the H/D-exchange reactions were too fast to follow by ^1^H NMR spectroscopy and significant hydrolytic ring-opening was observed. The H/D-exchange could be monitored in acetate buffers in the p*D* range 4.76–6.29 at 25 °C and ionic strength, *I* = 0.06 (KCl) for the prolyl TKP and *I* = 0.2 (KCl) for the glycyl TKP. Rates of deuterium exchange continued to decrease at lower p*D*s. Owing to the poor solubility of both TKPs in fully aqueous solution, kinetic studies were performed with a 40% d_3_-MeCN co-solvent (ESI†).

**Fig. 4:**
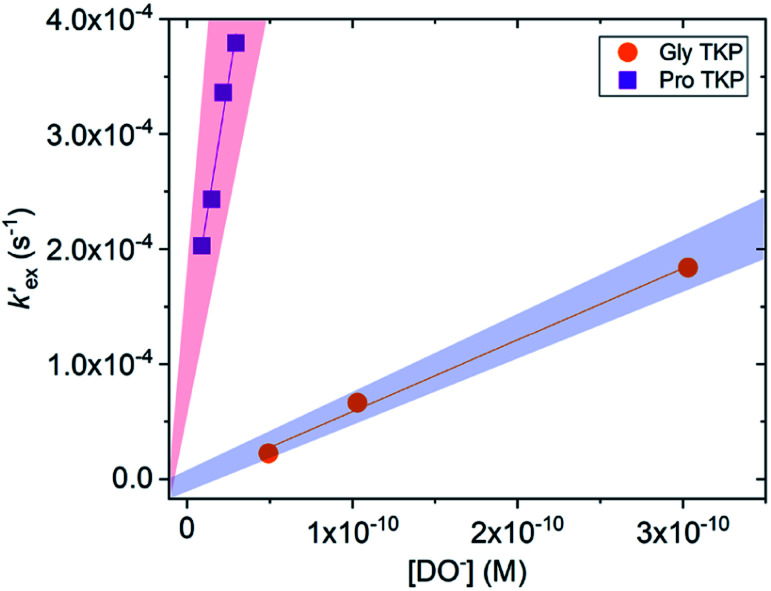
Plot of the buffer independent first order rate constants of exchange 
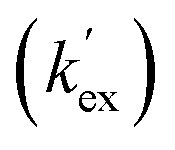
 against the concentration of deuteroxide for glycyl and prolyl TKPs in acetic acid buffer solutions with 40% d_3_-MeCN co-solvent, *I* = 0.2 (KCl) for Pro-TKP and *I* = 0.06 (KCl) for Gly–TKP and 25 °C. The red region indicates the kinetic acidity for prolyl TKP and the blue region indicates the kinetic acidity of the glycyl residue.

Unlike the DKPs, significant general base catalysis was observed for the TKPs. The *k*_DO_ values are obtained as the slope of a plot of buffer-independent 
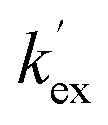
 values against DO^−^ concentration (Fig. 4). Individual 
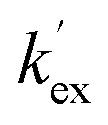
 were obtained as *y*-axis intercepts of plots of *k*^obs^_ex_ values *versus* buffer concentration ([AcO^−^]) at a constant p*D* (†). The *k*_DO_ values were ∼10^6^-fold higher for both TKPs compared with all the DKPs (Table 3). Furthermore, the prolyl effect was also observed in the TKPs, with the prolyl TKP being 17-fold more acidic than the glycyl TKP, indicating the generality of the prolyl effect in cyclic systems.

**Table 3:** Second order rate constants, *k*_AcO^−^_, *k*_DO_, and *k*_HO_, of the TKPs studied with 40% d_3_-MeCN co-solvent at 25 °C and *I* = 0.06–0.20 M (KCl)

	*k*_AcO^−^_ a (M^−1^ s^−1^)	*k*_DO_ (M^−1^ s^−1^)	*k*_HO_ b (M^−1^ s^−1^)
Gly TKP	7.21 × 10^−2^	5.09 × 10^5^	6.40 × 10^4^
Pro TKP	1.61 × 10^−2^	8.99 × 10^6^	1.14 × 10^6^

a *k*_AcO^−^_ is the second order rate constant for deprotonation by acetate anion and was obtained as the slope of *k*^obs^_ex_*versus* buffer concentration plots at p*D* 6.28 for Gly TKP and p*D* 4.76–5.28 for Pro TKP.

b A secondary solvent isotope effect of *k*_DO_/*k*_HO_ = 1.46 was used to convert *k*_DO_ to *k*_HO_.

The six orders of magnitude increase in *k*_DO_ is markedly larger than would be expected from an increased inductive effect caused by the inclusion of an additional carbonyl in the TKP *versus* DKP rings. Similarly, the 40% d_3_-MeCN co-solvent is expected to alter the observed rate constant by no more than ∼10-fold based on previous H/D-exchange studies of a range of carbon acids.^66^ The rate constants for enolate formation in TKPs (*k*_DO_ = 5.09 × 10^5^ to 8.99 × 10^6^ M^−1^ s^−1^) are closer to that for the formation of the aromatic phenolate anion from cyclohexa-2,5-dienone (*k*_HO_ = 2.0 × 10^6^ M^−1^ s^−1^).^67^

**Fig. 5:**
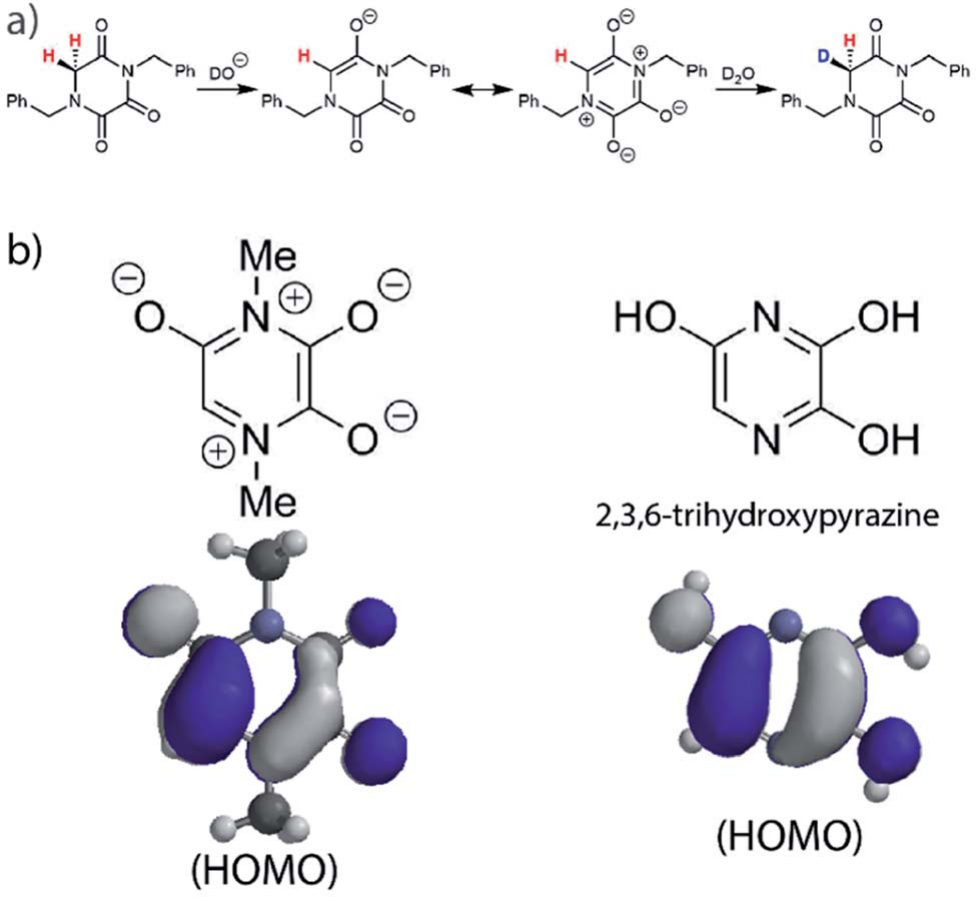
(a) Proposed mechanism of hydrogen–deuterium exchange *via* enolate formation for Gly TKP; (b) resonance structures and HOMOs for the fully delocalised TKP enolate and 2,3,6-trihydroxypyrazine.

In order to account for this significant rate enhancement, the contribution of aromaticity to transition state stabilisation is invoked. Our BMK/6-31+G calculations revealed the highly delocalised nature of the HOMO of the TKP enolate (Fig. 5, S3.2.4 and S3.2.5†), which clearly shares the same features as 2,3,6-trihydroxypyrazine, a structurally comparable aromatic model compound. The role of aromaticity in transition state stabilisation is well known *e.g.* for Diels–Alder reactions.^68^ A computational analysis of the contribution that aromaticity makes to the stability of the transition state for carbon acid deprotonation has been performed by Bernasconi and co-workers.^69^ Based upon these calculations, Bernasconi concluded that only minor progress in the formation of the product aromatic molecular orbitals is required in order for the transition state to take advantage of aromaticity for stabilisation. This is also supported by experimental observations.^70–74^ Thus, aromatic character could potentially stabilise the transition state for formation of the TKP enolate, prior to significant solvent reorganisation, explaining the substantially enhanced acidity of the α-protons.

### Brønsted linear free energy analysis: estimates for the α-carbon acid p*K*_a_s

**Fig. 6:**
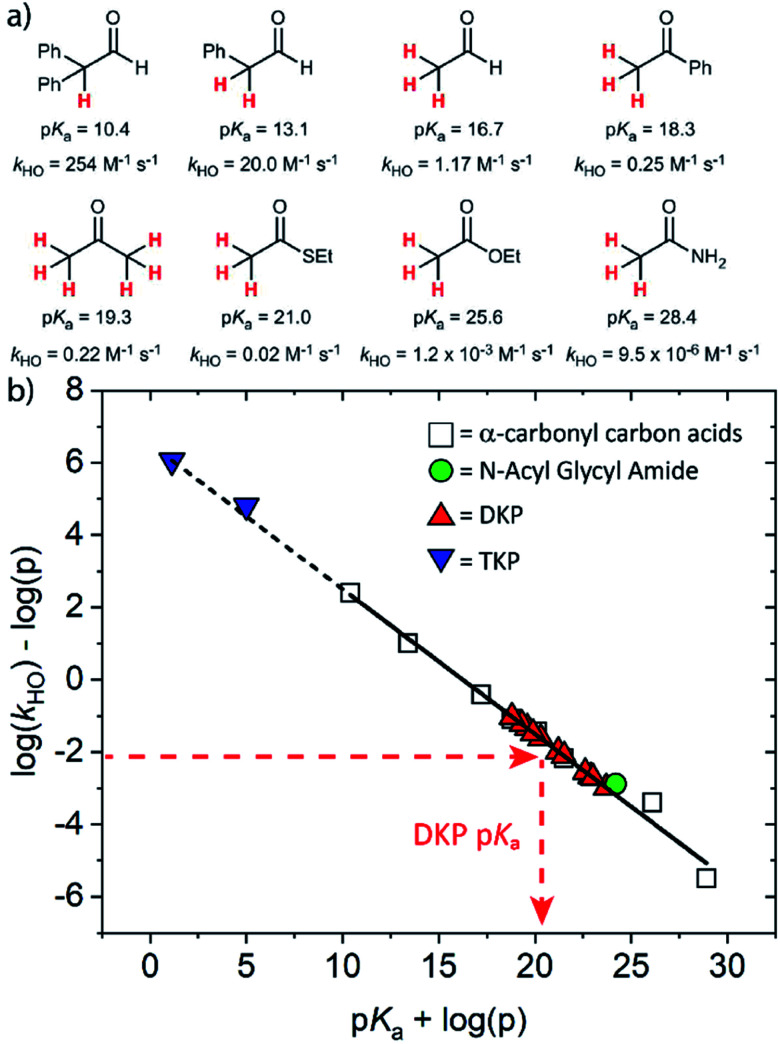
(a) α-Carbonyl compounds (□) used to construct the Brønsted LFER below with data from Richard and co-workers;^48,57–60,75–77^ (b) Brønsted linear free energy relationship between log(*k*_HO_/*p*) and p*K*_a_ for the series of α-carbonyl carbon acids above (□). The data is fitted with log(*k*_HO_/*p*) = −0.401p*K*_a_ + log(*p*) + 6.51 (—) where *p* = number of acidic α-CH protons. Kinetic data for the DKPs (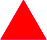) and *N*-acyl glycyl amide (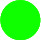) can be used to interpolate corresponding p*K*_a_ values using eqn (1), whereas kinetic data for TKPs (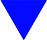) would require significant extrapolation.

The kinetic acidities (protofugalities) (*k*_DO_) measured above can be correlated to the thermodynamic p*K*_a_s of the carbon acids through a Brønsted linear free energy relationship (LFER). For the series of neutral α-carbonyl compounds in Fig. 6a, Richard and co-workers have reported an extended linear Brønsted correlation between experimental rate constants for hydroxide-catalysed α-deprotonation (*k*_HO_) and the corresponding carbon acid p*K*_a_s of a series of α-carbonyl acids (eqn (1); Fig. 6b, □) with β = −0.401.^77,78^1
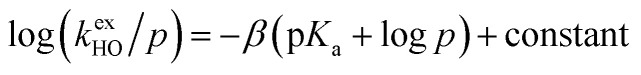


As DKPs are also neutral α-carbonyl acids, estimates of p*K*_a_ values can therefore be obtained from combining the experimental *k*_DO_ values for DKPs measured herein with the Brønsted LFER (Fig. 6b, 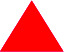). This assumes that a constant intrinsic barrier to proton transfer with changes in p*K*_a_, which underpins the linear Brønsted correlation observed for the series in Fig. 6a, also applies to the DKPs. Prior to use of the *k*_DO_ values on the Brønsted LFER, it is necessary to correct for a secondary solvent isotope effect (which typically range from *k*_DO_/*k*_HO_ = 1.36–2.40).^57,75^ We used an intermediate secondary solvent isotope effect of *k*_DO_/*k*_HO_ = 2.0 to estimate the *k*_HO_ values in Table 1, which is consistent with a specific base-catalysed proton transfer process.^48,57^ The interpolated p*K*_a_ values in Table 1 fall in the following ranges: p*K*_a_ (Pro) = 18.8–22.6; p*K*_a_ (Gly) = 19.9–21.2; p*K*_a_ (Ala) = 22.6–22.7; p*K*_a_ (Tyr) = 23.7.

Consistent with the observed higher rate constants for exchange for the Pro residues, the interpolated p*K*_a_s were 2–4 units lower than for other residues. Coote and Easton have previously calculated the α-carbon p*K*_a_s of c(Gly–Gly) and c(l-Ala–l-Ala) DKPs computationally as 24.0 and 26.1, respectively.^54^ The 3–4 unit difference from the interpolated values could potentially result from the solvation model used in computational calculations, or alternatively from the need to account for enhanced stereoelectronic effects upon cyclisation.

Using the same Brønsted analysis for TKPs requires an extensive 3–4 unit extrapolation (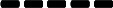) beyond the data in the existing correlation to predicted p*K*_a_s of 5.00 and 1.13 for the glycyl and prolyl TKPs, respectively (Fig. 6b, 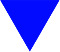). These extremely low predicted values would require the observation of the TKP enolate for p*D* values ≥ p*K*_a_, and rate-constants for re-protonation/deuteration should be slow relative to deprotonation. In our NMR experiments, deuterium exchange was clearly evident by the observation of an upfield triplet owing to α-CHD of the mono-deuterated glycyl TKP (Fig. S2.1†) showing that facile reprotonation occurs. Furthermore, no additional new NMR peaks were present that could be attributed to TKP enolate in both the glycyl and prolyl cases. This clearly demonstrates that the above Brønsted analysis is not appropriate for TKPs and highlights the significant difference of these systems from both DKPs and simple, non-cyclic α-carbonyl acids.

### Biological relevance

In addition to highlighting the uniquely high kinetic lability and acidity of α-protons in prolyl-containing cyclic peptides, these new data also present an alternative rationale for the origin of catalysis in amino acid-utilising enzymes. The carbon acidity of α-carbonyl compounds is crucial to many key biological reactions and an understanding of how enzymes enhance the rate of deprotonation is vital to probing metabolic processes.^55,79^ The restrictions on the conformation of an amino acid residue within a DKP has been shown herein to enhance the lability of the α-proton by up to 144-fold relative to a linear, non-cyclic reference, thereby reducing the barrier to deprotonation by up to ∼12.1 kJ mol^−1^. In the more restricted environment of an enzyme active site, a reduction in conformational mobility could similarly allow for enhanced σ_C–H_ and 
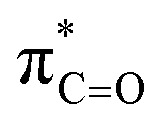
 interactions in non-cyclic, linear peptides and particularly for prolyl-containing systems. This provides an alternative potential origin for enzymatic rate enhancement in non-co-factor dependent amino acid racemases.^60,80^ Recently, a new type of prolyl hydroxylation at the C_α_-position was identified in the active site of polysaccharide deacetylases.^81^ The enhanced acidity of the prolyl group identified here could in principle aid the oxidation of the C_α_-position by molecular oxygen in order to form the 2-hydroxyproline.^82^

## Conclusions

We have demonstrated herein that the α-protons in Pro residues in DKP cyclic peptides have unexpectedly high rate constants of deprotonation, between 3 – 89-fold greater than other DKP amino acid residues studied. Evidence for the stereoelectronic origins of this prolyl effect was presented. Furthermore, our data highlights that cyclisation of a linear peptide substantially increases the labilities of the α-protons, which can be further enhanced by stereoelectronic factors especially in the case of Pro residues. Brønsted analysis was performed to provide estimates of DKP p*K*_a_s.

Our study also reveals the exceptionally enhanced α-kinetic acidity by up to six orders of magnitude of TKP cyclic peptides relative to DKPs, which can be rationalised by invoking an aromatic-like transition state for deprotonation in the former case. Rate constants for proton transfer in the TKPs are similar to that for the formation of the aromatic phenolate anion from cyclohexa-2,5-dienone. In TKP systems the enhanced acidity of the prolyl residue was also observed.

Our results thus demonstrate a new unique property for the chemistry of proline derivatives: the enhanced acidity of prolyl residue α-protons. The observation of this effect in both DKPs and TKPs indicates the generality of this effect in cyclic peptides. Future work by us will additionally probe the interplay between the effect of 4-Pro substitution, usually attributed to the *gauche* effect, and α-proton kinetic acidity.

## Experimental

Synthetic procedures, preparation of solutions, NMR and kinetic methods to monitor deuterium exchange reactions, kinetic evaluation and electronic structure calculations are found in the ESI.†

## Conflicts of interest

There are no conflicts to declare.

## Supplementary Material

SC-011-D0SC02508A-s001
