# Supplementary material for: Unusually high α-proton acidity of prolyl residues in cyclic peptides
Source: Chem Sci. 2020 Jul 2;11(29):7722–9. doi: 10.1039/d0sc02508a (PMC8159430; doi:10.1039/d0sc02508a)
Supplement: SC-011-D0SC02508A-s001 [file SC-011-D0SC02508A-s001.pdf]

Electronic Supplementary Material (ESI) for Chemical Science.
This journal is © The Royal Society of Chemistry 2020

Supplementary Information

Unusually high α-carbon acidity of prolyl residues in cyclic peptides

Oliver R. Maguire,[a,b] Bethany Taylor,[a] Eleanor M. Higgins,[a] Matthew Rees,[c] Steven L. Cobb,[a] Nigel S. Simpkins[c] Christopher J. Hayes[d] and AnnMarie C. O'Donoghue[*a]

[a] Department of Chemistry, Durham University, University Science Laboratories, South Road, Durham DH1 3LE, U.K.

[b] Current address: Radboud University Nijmegen, Institute for Molecules and Materials, Heyendaalseweg 135, 6525 AJ Nijmegen, The Netherlands.

[c] School of Chemistry, University of Birmingham, Edgbaston, Birmingham B15 2TT, U.K.

[d] School of Chemistry, University of Nottingham, University Park, Nottingham, NG7 2RD, U.K.

*Correspondence to: annmarie.odonoghue@durham.ac.uk

Table of Contents

Table of Contents **2**

S1 Hydrogen Deuterium Exchange studies on Diketopiperazines **4**

S1.1 General Instrumentation *4*

S1.2 Materials *4*

S1.3 DKP Synthesis *5*

S1.3.1 Synthesis of c(D-Pro-L-Pro) 5

S1.3.2 Synthesis of c(D-Pro-D-Pro) 7

S1.4 Kinetic Experiments *7*

S1.4.1 Solution Preparation 7

S1.4.2 Measurement of pH 7

S1.4.3 NMR experimental conditions 8

S1.4.4 The deuterium exchange experiment with DKPs 8

S1.4.4.1 Analysis of experimental error 10

S1.4.5 Hydrogen-deuterium exchange reactions of c(D-Pro-L-Pro) 11

S1.4.6 Hydrogen-deuterium exchange reactions of c(D-Pro-D-Pro) 26

S1.4.7 Hydrogen-deuterium exchange reactions of c(D-Ala-L-Pro) 30

S1.4.7.1 c(D-Ala-L-Pro) Prolyl α-proton 31

S1.4.7.2 c(D-Ala-L-Pro) Alanyl α-proton 50

S1.4.8 Hydrogen-deuterium exchange reactions of c(L-Pro-L-Tyr) 54

S1.4.8.1 c(L-Pro-L-Tyr) Prolyl α-proton 55

S1.4.8.2 UV-Vis Spectrophotometric determination of the tyrosyl phenolic pK_a on c(L-Pro-L-Tyr) 57

S1.4.8.3 c(L-Pro-L-Tyr) Tyrosyl α-proton 67

S1.4.9 Hydrogen-deuterium exchange reactions of c(Gly-L-Pro) 71

S1.4.9.1 c(Gly-L-Pro) Prolyl α-proton 71

S1.4.9.2 c(Gly-L-Pro) Glycyl α-protons 76

S1.4.10 Hydrogen-deuterium exchange reactions of c(Gly-Gly) 82

S1.4.11 Hydrogen-deuterium exchange reactions of c(L-Ala-L-Ala) 86

S2 Hydrogen Deuterium Exchange studies on Triketopiperazines **90**

S2.1 General Instrumentation *90*

S2.2 Materials *90*

S2.3 Synthesis of Triketopiperazines *90*

S2.4 Kinetic Experiments *90*

S2.4.1 Solution Preparation 91

S2.4.2 Measurement of pH 91

S2.4.3 NMR experimental conditions 92

S2.4.3.1 Analysis of experimental error 92

S2.4.4 Deuterium exchange reactions of TKPs 93

S2.4.5 First order rate constants for deuterium exchange, k_{ex}, of Glycyl TKP 97

S2.4.6 Estimates for the second order rate constant for deuteroxide catalysed exchange, k_{DO}, for Glycyl TKP 102

S2.4.7 First order rate constants for deuterium exchange, k_{ex}, for Prolyl TKP 104

S2.4.8 Second order rate constants for buffer catalysed, k_{A-}, and deuteroxide catalysed, k_{DO}, exchange for Prolyl TKP 119

S2.4.9 Converting the experimental k_{DO} values to k_{HO} values in Table 3 of the Main Text 123

S3 Computational Results **124**

S3.1 Computational Methods 124

S3.2 Geometry Optimisations using BMK/6-31+G(d) 124

S3.2.1 Gly-L-Pro 124
S3.2.2 Gly-L-Pro (Pro enolate) 125
S3.2.3 Gly-L-Pro (Gly enolate) 125
S3.2.4 2,3,6-Trihydroxypyrazine 126
S3.2.5 TKP enolate 127
S3.2.6 c(D-Pro-D-Pro) + LiOH 128
S3.2.7 c(D-Pro-L-Pro) + LiOH 129
S3.2.8 c(D-Pro-D-Pro) + LiOH (Transition State) 130
S3.2.9 c(D-Pro-L-Pro) + LiOH (Transition State) 131

S3.2 Analysis of Computational Results 132

S4 References 133

S1 Hydrogen Deuterium Exchange studies on Diketopiperazines

S1.1 General Instrumentation

NMR: Synthetic and Kinetic NMR spectra were recorded on Bruker Ultrashield 400 MHz, Varian 400 MHz and Oxford Varian Unity Inova 500 MHz NMR spectrometers. High Resolution ^{1}H NMR, ^{13}C NMR, COSY, HSQC, HMBC spectra were recorded on a Varian Inova 700 MHz NMR spectrometer. NMR samples were prepared in D_2O and $CDCl_3$. All chemical shifts are reported relative to residual solvent peaks of δ_H 4.79 ppm for D_2O and δ_H 7.26 ppm and δ_C 77.2 ppm for $CDCl_3$.

Mass Spectrometry: A Waters TQD mass spectrometer was used to record low resolution mass spectra. A Thermo-Finnigan LTQ FT mass spectrometer was used to record high resolution mass spectra.

UV-Vis Spectrophotometry: A Cary 100 UV-Vis spectrophotometer with a temperature regulated cuvette holder and attached heating unit was used to collect all absorbance spectra.

pH Measurement: A MeterLab^{TM} PHM 290 pH-Stat Controller equipped with a radiometer combination electrode filled with saturated KCl solution was used to measure pH.

Melting Point: Melting point measurements were made on a Gallenkamp melting point apparatus.

S1.2 Materials

NMR Solvents: d_2-Deuterium oxide (99.9 atom % D) and d_3-acetonitrile (99.8 atom % D) were purchased from Goss Scientific. Chloroform-d_1 (99.8 atom % D), potassium deuteroxide (99.8 atom % D, 40 wt %) and deuterium chloride (99 atom % D, 35 wt %) were purchased from Sigma-Aldrich. A stock solution of 2 M potassium chloride was prepared by dissolving solid potassium chloride in D_2O. Stock solutions of potassium deuteroxide and deuterium chloride were prepared by dissolving concentrated potassium deuteroxide and deuterium

chloride in D_2O and then titrating against volumetric hydrochloric acid and volumetric sodium hydroxide solutions, respectively, to determine the concentration of KOD and DCl.

Synthetic Reagents: L-Proline benzyl ester hydrochloride salt, D-proline benzyl ester hydrochloride salt, *N*-carboxybenzyl-D-proline and (benzotriazol-1-yloxy)-tripyrrolidinophosphonium hexafluorophosphate (PyBOP) were purchased from TCI UK. Dichloromethane AR grade, ethyl acetate reagent grade, methanol HPLC-grade were purchased from Fisher Scientific. Pd/C 10 % catalyst and thionyl chloride were purchased from Sigma Aldrich.

Kinetic Experiments: Potassium carbonate, 3-chloroquinuclidine hydrogen chloride salt, potassium chloride, tetramethylammonium hydrogensulfate, sodium trimethylsilylpropyl sulfonate and c(Gly-Gly) were purchased from Sigma Aldrich. c(D-Ala-L-Pro), c(L-Ala-L-Ala), c(L-Pro-L-Tyr) and c(Gly-L-Pro) were purchased from Bachem.

S1.3 DKP Synthesis

S1.3.1 Synthesis of c(D-Pro-L-Pro)

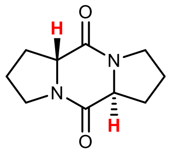

A round bottom flask was charged with L-proline benzyl ester hydrochloride salt (243 mg, 1.0 mmol) which was then partially dissolved in 5 mL of dry dichloromethane. *N*-Methylmorpholine (110 µL, 1.0 mmol, 1 eq) was added to the solution along with *N*-carboxybenzyl-D-proline (250 mg, 1.0 mmol, 1 eq). (Benzotriazol-1-yloxy)tripyrrolidinophosphonium hexafluorophosphate, PyBOP, (570 mg, 1.1 mmol, 1.1 eq) was added to the solution and the reaction was left to stir at 25 °C under an inert argon atmosphere for 22 h. Solvent was removed under reduced pressure to give a viscous yellow oil. Upon dissolving the crude yellow oil in ethyl acetate a white precipitate was formed and this was removed via gravity filtration. Oil was purified upon a silica column (ethyl acetate 100 %) to give benzyl-2-([2-benzyloxycarbonylpyrrodin-1-yl]carbonyl)pyrrolidine-1-carboxylate (211 mg, 0.5 mmol, yield 48 %, R_f 0.35). This procedure was repeated with 1 eq of PyBOP (520 mg, 1.0 mmol) to give a further amount of benzyl-2-([2-

benzyloxycarbonylpyrrodin-1-yl]carbonyl)pyrrolidine-1-carboxylate (135 mg, 0.3 mmol, yield 31 %).

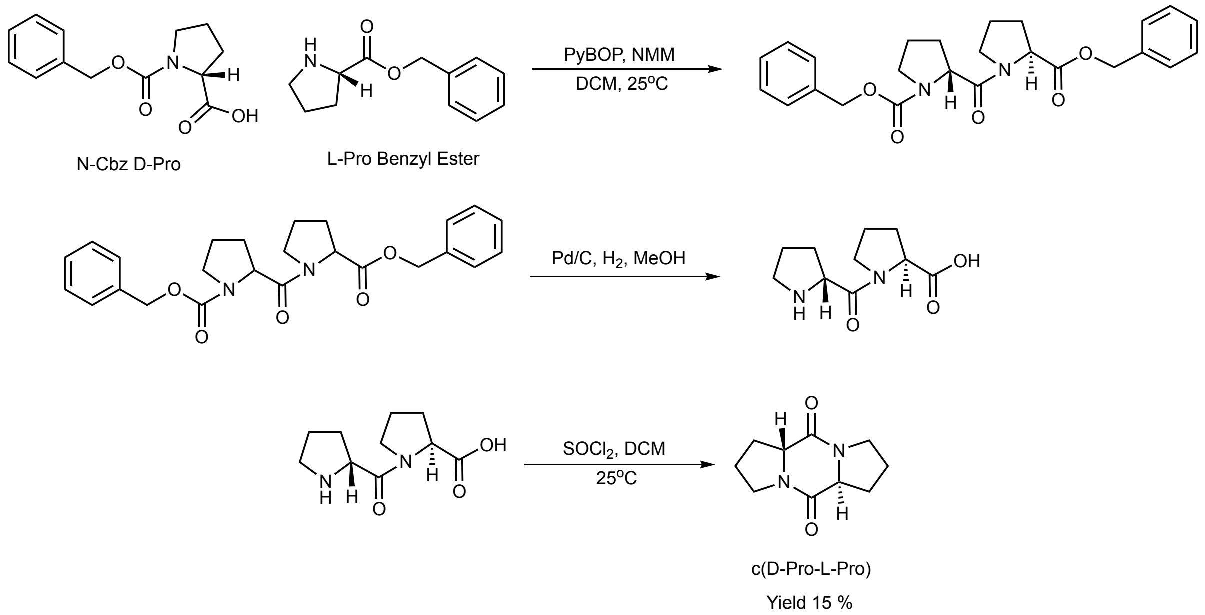

Scheme S1: Preparation of c(D-Pro-L-Pro). The synthesis of c(D-Pro-D-Pro) used an identical route but with L-Pro benzyl ester replaced with D-Pro benzyl ester.

Both of the benzyl-2-([2-benzyloxycarbonylpyrrodin-1-yl]carbonyl)pyrrolidine-1-carboxylate (346 mg, 1.3 mmol) were combined and dissolved in HPLC grade methanol (15 mL). Pd/C 10 % catalyst (83 mg) was added and solution was subjected to hydrogenation at 40 bar pressure for 12 h. The solution was filtered three times to remove Pd/C catalyst and then solvent was removed under reduced pressure to leave a white solid, 1-([pyrrodin-2-yl]carbonyl)pyrrolidine-2-carboxylic acid (179 mg, 0.8 mmol, 100 % Yield).

1-([Pyrrodin-2-yl]carbonyl)pyrrolidine-2-carboxylic acid (179 mg , 0.8 mmol) was dissolved in 5 mL of dichloromethane and thionyl chloride (70 µL, 1.0 mmol, 1.2 eq) was added dropwise. The solution was left to stir for 48 h at 25 °C. The solvent was removed under reduced pressure to leave a light brown solid, which was dissolved again in dichloromethane and extracted with 5 % aqueous HCl solution. The organic layer was removed and concentrated under reduced pressure to leave the title compound as a light yellow solid (48 mg, 0.25 mmol, yield 15 %). The enantiopurity of the c(D-Pro-L-Pro) was confirmed using chiral HPLC. **^{1}H NMR** (400 MHz, D_2O): δ_H 1.66-2.00 (6H, m), 2.15-2.23 (2H, m), 3.43-3.52 (2H, m), 3.88-3.98 (2H, m), 4.29-4.36 (2H, m); ***m/z*** (ES+): $[M–H]^+$ $C_{10}H_{15}N_2O_2$ requires 194.2 found 194.2. Melting Point: 188 – 190 °C (Literature: 179 – 181 °C).[1]

S1.3.2 Synthesis of c(D-Pro-D-Pro)

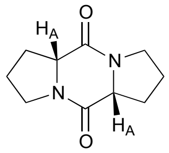

c(D-Pro-D-Pro) was prepared using an identical method to c(D-Pro-L-Pro) as detailed above, but with D-proline benzyl ester hydrochloride salt used as a starting reagent instead. The title compound was obtained as a white solid (31 mg, 0.40 mmol, yield 12 %). The enantiopurity of the c(D-Pro-D-Pro) was confirmed using chiral HPLC. **^{1}H NMR** (400 MHz, D_2O): δ_H 1.95-2.15 (6H, m), 2.27-2.40 (2H, m), 3.41-3.52 (2H, m), 3.55-3.63 (2H, m), 4.44-4.52 (2H, m); ***m/z*** (ES+): $[M–H]^+$ $C_{10}H_{15}N_2O_2$ requires 194.2 found 194.2.

S1.4 Kinetic Experiments

S1.4.1 Solution Preparation

Potassium carbonate ($KDCO_3$ / K_2CO_3) buffer solutions were prepared by dissolving solid potassium carbonate in D_2O. Deuterium chloride solution was used to adjust the percentage of free base form of the buffer. A 2 M potassium chloride solution was employed to adjust to ionic strength $I = 1.0$.

3-Chloroquinuclidine buffer solutions were prepared from 3-chloroquinuclidine deuterium chloride salt (prepared from 3-chloroquinuclidine hydrogen chloride salt by exchanging the N-H for a N-D with D_2O using a freeze-drying procedure) and a potassium deuteroxide solution. A 2 M potassium chloride solution was employed to adjust to ionic strength $I = 1.0$.

Tetramethylammonium deuteriosulfate and sodium trimethylsilylpropyl sulfonate internal standard solutions were prepared with D_2O and a 2 M potassium chloride solution to adjust to ionic strength $I = 1.0$.

S1.4.2 Measurement of pH

The pH of the buffered solutions was determined using a MeterLab™ PHM 290 p*H*-Stat Controller equipped with a radiometer combination electrode filled with saturated KCl

solution at 25 ºC. The pH meter was calibrated with pH 7 potassium phosphate and pH 12.45 saturated calcium hydroxide solutions. The p*D* values were calculated by adding 0.4 to the pH meter reading.[2] An activity coefficent for the deuteroxide ion (γ_{DO}) of 0.722 and a pK_w(D_2O) = 14.951 was used to determine the concentration of deuteroxide ion from Eqn. S1:

$$\gamma_{OH}[OD^-] = 10^{[pD - pK_w(D_2O)]} \qquad \text{Eqn. S1}$$

S1.4.3 NMR experimental conditions

The hydrogen deuterium exchange experiments were carried out on either a Varian 400 MHz or a Varian 500 MHz NMR spectrometer. A pulse sequence with a 20 s relaxation delay, an acquisition time of 4 s and a 90º pulse angle was employed. A total of 64 transients were taken for each spectrum (a total acquisition time of ~ 25 min 30 s). To correct for any exchange that took place between the first and last transient the measurement times, *t*, for the exchange experiments were calculated from the mid-point of the acquisition. ^{1}H NMR spectral baselines were subject to a first-order drift correction before integration of the peak areas. The integration of substrate peak areas was compared to the peaks of the tetramethylammonium deuteriosulfate or sodium trimethylsilylpropyl sulfonate internal standard with an arbitrary value of 1000 for the integration of the internal standard.

S1.4.4 The deuterium exchange experiment with DKPs

Deuterium exchange reactions were carried out in carbonate ($KDCO_3$ / K_2CO_3) or 3-chloroquinuclidine buffered D_2O solutions in the p*D* range 9.35 – 10.98 at 25 °C and ionic strength *I* = 1.0 M. Reactions were initiated by the addition of buffer solution to the DKP and the internal standard (either tetramethylammonium deuteriosulfate or sodium trimethylsilylpropyl sulfonate, both in D_2O). Typical concentrations of DKP and internal standard were 5.0 mM and 0.4 mM respectively. A portion of the resulting solution was extracted and placed in a sealed NMR tube and thermostated at 25 ± 0.1 °C in a waterbath. The remainder of the solution was kept in the same waterbath and used to monitor the p*D* of the solution during the course of the reaction. pD values recorded were found to be constant within error (±0.03). To monitor the extent of deuterium exchange ^{1}H NMR spectroscopy was employed. Deuterium exchange was followed until the disappearance of typically 75−90% of the signal of the most acidic alpha proton. The integrated areas of signals from other protons in the DKPs did change due to diasteromeric interconversion – this is discussed below in the

experimental results for each individual diketopiperazine (Section S1.4.5 – S1.4.11). Only minor amounts of hydrolysis were observed (< 10 % after 2 weeks).

The extent of deuterium exchange was evaluated by monitoring the disappearance of signal from α-protons relative to a peak from the internal standard. The fraction of unreacted substrate remaining at each time point, *f*(s), was determined via Equation S2:

$$f(\mathrm{s}) = \frac{(A_{\alpha-\mathrm{H}}/A_{\mathrm{int}})_t}{(A_{\alpha-\mathrm{H}}/A_{\mathrm{int}})_{t=0}} \quad \text{Eqn. S2}$$

where $A_{\alpha\text{-H}}$ is the integral of the α-proton, A_{int} is the integral of the relevant peak on the internal standard and *t* is the time. Experimental pseudo first order rate constants of exchange, k_{ex}, of the α-protons were determined from the variation of *f*(s) with time via a fit of Eqn. S3 to the reaction data:

$$f(\mathrm{s}) = \exp{(-k_{\mathrm{ex}}t)} \quad \text{Eqn. S3}$$

For c(L-Pro-L-Tyr) the pK_a (10.68) of the phenol in D_2O was determined via UV-Vis spectrophotometry (See S1.4.8.2) and the observed k_{ex} (s^{-1}) was corrected for the fraction of c(L-Pro-L-Tyr) in neutral form.

The experimental pseudo first order rate of rate constant of exchange, k_{ex}, can potentially have kinetic contributions from the exchange pathways of three different components of solvent: solvent-promoted routes (k_{D2O}), acid catalysed ($k_{\mathrm{D3O+}}$), and base catalysed (k_{DO}) (Eqn. S4).

$$k_{\mathrm{ex}} = k_{\mathrm{D_2O}} + k_{\mathrm{D3O+}}[\mathrm{D_3O^+}] + k_{\mathrm{DO}}[\mathrm{DO^-}] \quad \text{Eqn. S4}$$

For the deuterium exchange of the α-protons in the DKPs, the values of k_{ex} increased with the p*D* of the solution indicating that the base catalysed rather than acid catalysed route was dominant. In addition, the uncatalysed pD independent route for exchange is typically negligible for most weakly acidic carbon acids and therefore Equation S4 can be simplified to Eqn. S5.

$$k_{\mathrm{ex}} = k_{\mathrm{DO}}[\mathrm{DO^-}] \quad \text{Eqn. S5}$$

Thus second order rate constants for deuteroxide-catalysed exchange, k_{DO}, can be determined as the slope of a linear correlation of k_{ex} and the concentration of deuteroxide ion. Basic buffer components may also contribute to k_{ex} (Eqn. S6):

$$k_{ex} = k_{DO}[DO^-] + k_{buff}[A^-] \qquad \text{Eqn. S6}$$

where k_{buff} is the second order rate constant for general base-catalysed exchange and $[A^-]$ is the concentration of the base form of the buffer.

S1.4.4.1 Analysis of experimental error

There are several possible sources of experimental error:

- **Temperature variation:** The majority of the deuterium exchange experiments were carried out on a 400 MHz NMR spectrometer that was not thermostated. Care was taken to minimise the effect of temperature changes when the samples were removed from the thermostated waterbath. In transit between the waterbath and the NMR instrument the sealed NMR tubes were incubated in a container at 25 °C. The room which housed the NMR spectrometer was thermostated but the temperature did fluctuate by ~ ± 2 °C. The impact of the variation in temperature is expected to be minimal. The exchange experiments typically took two weeks to complete and with only one measurement taken a day (duration of acquisition ~ 25 min) the time spent out of the waterbath is less than 1.5 % of the total length of the experiment. The exchange experiments undertaken on a 500 MHz machine were thermostated at 25 ± 1 °C.
- **Variation in p*D*:** For exchange reactions in 3-chloroquinuclidine buffers there was no change in p*D* of the solutions over two weeks. Small changes in p*D* (usually < 0.04) were sometimes observed for reactions in the carbonate buffers over the same timescale.
- **Variation in ionic strength:** In order to prepare the carbonate buffers ($KDCO_3$ / K_2CO_3) a concentrated DCl solution was added to a K_2CO_3 solution. Upon addition of DCl the solution did effervesce slightly. A high local concentration of acid can unavoidably cause a sublimation reaction with the carbonate ($2D^+ + CO_3^{2-} \rightarrow D_2O + \uparrow CO_2$). The effect of this loss on the buffer capacity of the solution is expected to be minimal as the concentration of buffer used was 0.185 M and little effervescence was observed. The effect of the loss on ionic strength will be higher than on buffer capacity

as the contribution an ion makes to the ionic strength varies according to the square of its charge.[i] The loss of carbonate in this manner is not expected to significantly affect results, as the extent of effervescence observed was small and reliable pD values were obtained.

- **Initial values at t = 0 s:** the integrals used for the $t = 0$ values were obtained from data acquired ~ 16 minutes after initiation of the exchange experiment. The error introduced due to this time delay in acquisition is negligible as the exchange experiments typically took two weeks to complete.

Overall the sources of experimental error were minimised as much as possible and their cummulative effect on the results of the deuterium exchange experiments are small.

Where possible the exchange was monitored via the changes in integrals of peaks owing to multiple different protons on the DKPs. The k_{DO} values in these cases showed good agreement with the maximum variation between values being ±20 % and at lower end differences of ±5 %. R^2 values were ~0.99 for almost all fits of the reaction data to exponential first order decay.

S1.4.5 Hydrogen-deuterium exchange reactions of c(D-Pro-L-Pro)

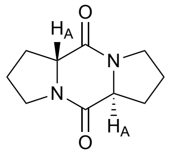

Pseudo first order rate constants for the deuteroxide catalysed exchange, k_{ex}, of the prolyl α-protons H_A of c(D-Pro-L-Pro) were determined in $KDCO_3$ buffered D_2O solutions (pD = 10.09 – 10.98) using ^{1}H NMR spectroscopy (400 and 500 MHz).

A representative set of spectra taken at three time points during the reaction at pD = 10.59 is shown in Figure S1.1. Deuterium exchange at the H_A position resulted in disappearance of the doublet of doublets due to H_A at 4.17 ppm over the course of the reaction. The level of exchange was monitored relative to a tetramethylammonium deuteriosulfate internal standard, whose methyl protons (3.14 ppm) are non-exchangeable. There are two possible products of exchange, *cis*-isomers and enantiomers c(D-Pro-D-Pro) or c(L-Pro-L-

[i] Ionic strength is calculated via $I = \Sigma$ (n c z^2), where n is amount of the ion in the chemical formula, c = concentration of the ion and z is the charge on the ion.

Pro), both of which are diastereomers of c(D-Pro-L-Pro) (Scheme S2). *Trans*-isomer, c(D-Pro-L-Pro), was found to be unstable relative to the two *cis*-diastereomers under our conditions in agreement with a previous literature study of the equilibrium distribution of c(Pro-Pro) DKP *cis*- and *trans*-isomers in aqueous solution.[3] Consequently, ^{1}H NMR spectra over the course of the reaction showed the disappearance of signals corresponding to the pyrrolidine ring protons (H_B, $H_{B'}$, H_C, $H_{C'}$ H_D and $H_{D'}$) on c(D-Pro-L-Pro) and the appearance of signals for analogous pyrrolidine ring protons (H_2, $H_{2'}$, H_3, $H_{3'}$ H_4 and $H_{4'}$) on c(D-Pro-D-Pro) / c(L-Pro-L-Pro) (Figure S1.1).

Figure S1.1: Representative ^{1}H NMR spectra at 500 MHz for the deuterium exchange reaction c(D-Pro-L-Pro) in $KDCO_3$ (50% free base, 0.185 M, p*D* = 10.59) buffered D_2O solution at 25 °C and ionic strength *I* = 1.0 M (KCl).

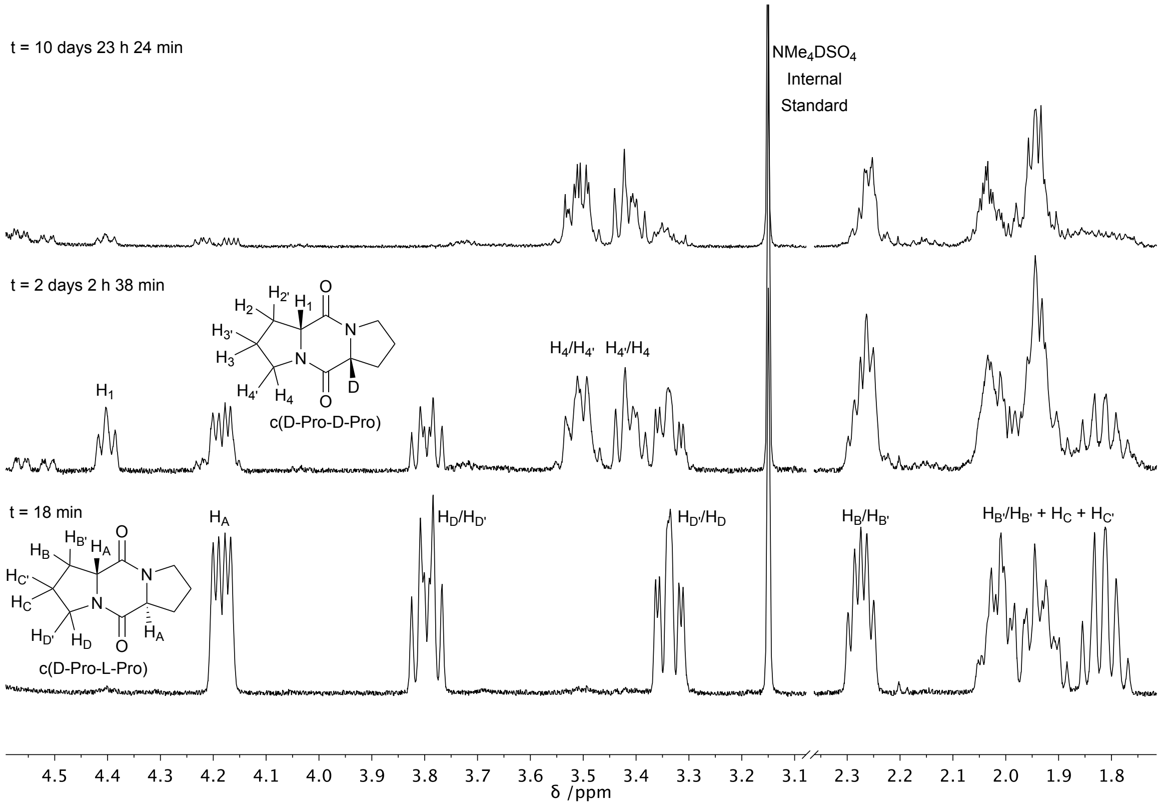

Scheme S2: Outcomes of the deuterium exchange reaction of c(D-Pro-L-Pro).

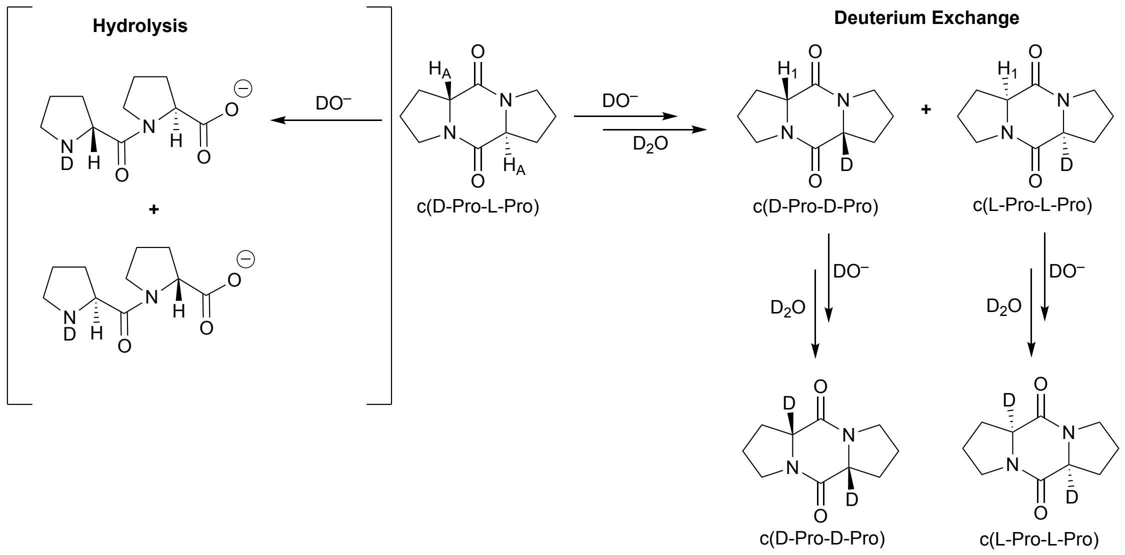

A minor amount of hydrolysis product (< 12 % after 10 days) was also observed in the ^{1}H NMR spectra. A proton on the hydrolysis product had a closely similar chemical shift to the H_A doublet of doublets. Consequently, deuterium exchange could not be precisely monitored via the H_A proton. Instead the exchange was followed via the disappearance of the multiplet for the pyrrolidine protons $H_D/H_{D'}$ (3.79 ppm) on c(D-Pro-L-Pro) and the appearance of the multiplet for the $H_4/H_{4'}$ (3.50 ppm) on c(D-Pro-D-Pro) / c(L-Pro-L-Pro). The rate limiting step for the deuterium exchange in Scheme S2 will be deprotonation of H_A on c(D-Pro-L-Pro) and therefore $H_D/H_{D'}$ and $H_4/H_{4'}$ provide a reliable alternative assessment for deuterium exchange.

Experimental first order rate constants for deuteroxide-catalysed deuterium exchange, k_{ex}, of prolyl α-protons H_A of c(D-Pro-L-Pro) were determined from the fit of the fraction of protonated substrate remaining, $f(s)$, for $H_D/H_{D'}$ c(D-Pro-L-Pro) and the fraction of deuterated product appearing, $f(p)$[ii], for $H_4/H_{4'}$ c(D-Pro-D-Pro) / c(L-Pro-L-Pro) to Eqn. S3 (Figures S1.2 and S1.3). Reaction data and k_{ex} values for $H_D/H_{D'}$ and $H_4/H_{4'}$ over the pD range pD = 10.09 – 10.98 are shown in Table S1.1 and Table S1.2, respectively.

[ii] Using $f(s) = 1 - f(p)$, where $f(p)$ is the fraction of product appearing.

Figure S1.2: Plot of the fraction, *f*(s), of $H_D/H_{D'}$ on c(D-Pro-L-Pro) converted to c(D-Pro-D-Pro) / c(L-Pro-L-Pro) against time for c(D-Pro-L-Pro) (5.0 mM) in $KDCO_3$ (50 % fb, 0.185 M, p*D* = 10.59) buffered D_2O solution at *I* = 1.0 (KCl) and 25 ºC.

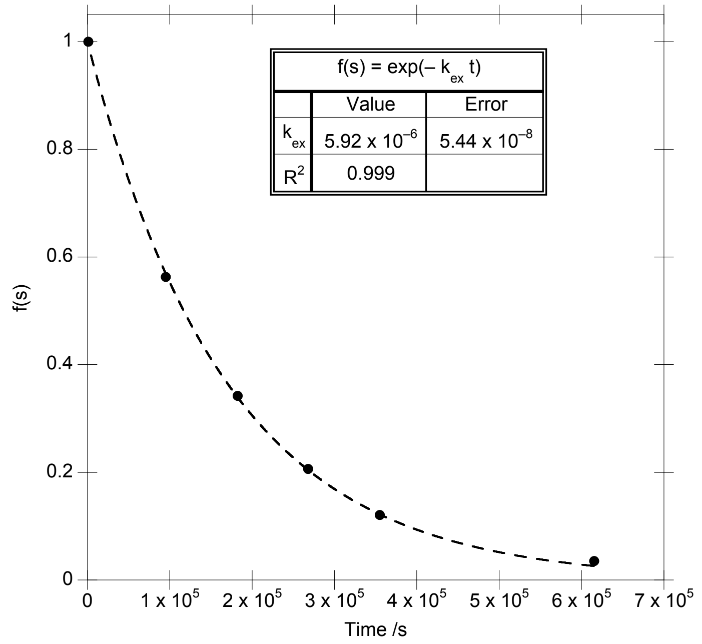

Figure S1.3: Plot of *f*(s) = 1 – *f*(p) for appearance of $H_4/H_{4'}$ on c(D-Pro-D-Pro) / c(L-Pro-L-Pro) over time for c(D-Pro-L-Pro) (5.0 mM) in $KDCO_3$ (50 % fb, 0.185 M, p*D* = 10.59) buffered D_2O solution at *I* = 1.0 (KCl) and 25 ºC.

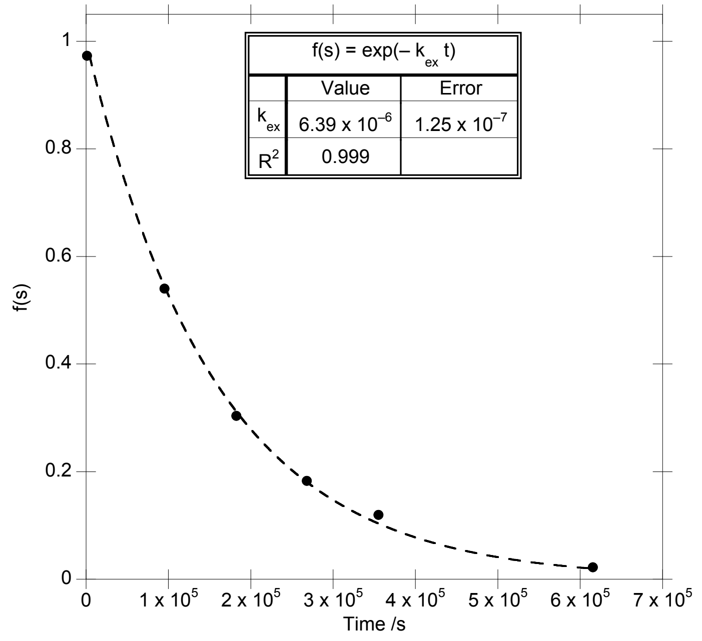

Table S1.1: Reaction data and observed first order rate constants (k_{ex}) for the deuterium exchange of the prolyl α-protons H_A in c(D-Pro-L-Pro) (5.0 mM) monitored via $H_D/H_{D'}$ pyrrolidine protons on c(D-Pro-L-Pro) in $KDCO_3$ (0.185 M, pD = 10.09 – 10.83) buffered D_2O solution, I = 1.0 (KCl) and 25 ºC.

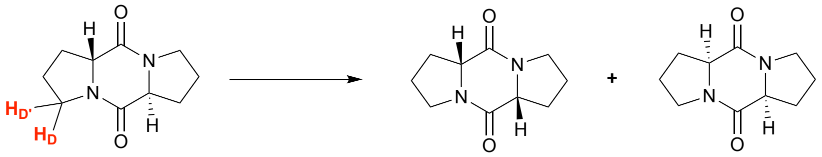

Experiment	Proton Chemical Shift /ppm	$[DO^-]$ /M	Time /s	$f(s)$[b]	k_{ex} /s^{-1}
70 % fb [a]	3.75 – 3.83	1.04×10^{-4}	1.00×10^{3}	1.00	1.05×10^{-5}
		(pD 10.83)	9.48×10^{4}	0.38	
			1.82×10^{5}	0.15	
			2.68×10^{5}	0.06	
			3.55×10^{5}	0.02	
60 % fb	3.75 – 3.83	1.02×10^{-4}	1.19×10^{3}	1.00	9.84×10^{-6}
		(pD 10.82)	1.25×10^{4}	0.89	
			1.93×10^{4}	0.83	
			7.93×10^{4}	0.45	
			9.33×10^{4}	0.40	
			1.06×10^{5}	0.35	
			1.64×10^{5}	0.19	
			1.82×10^{5}	0.17	
			1.92×10^{5}	0.15	
			2.52×10^{5}	0.09	
			2.67×10^{5}	0.07	
			3.38×10^{5}	0.03	
			3.66×10^{5}	0.02	
50 % fb	3.75 – 3.83	5.97×10^{-5}	1.07×10^{3}	1.00	5.92×10^{-6}
		(pD 10.59)	9.50×10^{4}	0.56	
			1.82×10^{5}	0.34	
			2.68×10^{5}	0.21	
			3.55×10^{5}	0.12	
			6.15×10^{5}	0.04	
35 % fb	3.75 – 3.83	4.27×10^{-5}	1.06×10^{3}	1.00	4.26×10^{-6}
		(pD 10.44)	9.75×10^{4}	0.65	
			1.85×10^{5}	0.45	

			2.76×10^5	0.31	
			3.68×10^5	0.21	
			6.23×10^5	0.08	
			7.08×10^5	0.06	
			7.86×10^5	0.04	
			8.75×10^5	0.05	
			9.72×10^5	0.02	
20 % fb	3.75 – 3.83	3.81×10^{-5}	1.06×10^3	1.00	3.86×10^{-6}
		(p*D* 10.39)	9.56×10^4	0.69	
			1.81×10^5	0.50	
			2.73×10^5	0.35	
			3.64×10^5	0.25	
			6.19×10^5	0.09	
			7.05×10^5	0.07	
			7.84×10^5	0.04	
			8.77×10^5	0.03	
			9.68×10^5	0.02	
22 % fb	3.75 – 3.83	1.91×10^{-5}	1.19×10^3	1.00	2.09×10^{-6}
		(p*D* 10.09)	1.81×10^4	0.98	
			7.80×10^4	0.85	
			1.63×10^5	0.58	
			2.56×10^5	0.71	
			3.37×10^5	0.49	
			6.01×10^5	0.28	
			6.83×10^5	0.24	
			7.76×10^5	0.19	
			8.56×10^5	0.16	
			9.42×10^5	0.13	
			1.22×10^6	0.08	
			1.40×10^6	0.06	

[a] fb = free base. [b] fraction of unreacted substrate remaining at each time point.

Table S1.2: Reaction data and observed first order rate constants (k_{ex}) for the deuterium exchange of the prolyl α-protons H_A in c(D-Pro-L-Pro) (5.0 mM) monitored via $H_4/H_{4'}$ pyrrolidine protons on c(D-Pro-D-Pro) / c(L-Pro-L-Pro) in $KDCO_3$ (0.185 M, p*D* = 10.09 – 10.83) buffered D_2O solution, *I* = 1.0 (KCl) and 25 ºC.

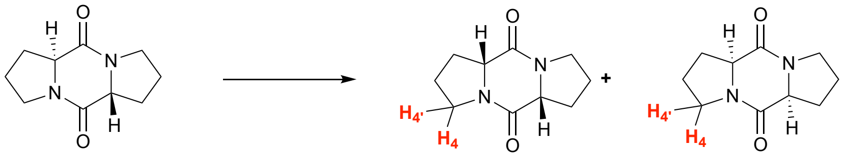

Experiment	Proton Chemical Shift /ppm	$[DO^-]$ /M	Time /s	*f*(s)[b]	k_{ex} /s^{-1}
70 % fb [a]	3.46 – 3.53	1.04×10^{-4}	1.00×10^{3}	0.96	1.09×10^{-5}
		(p*D* 10.83)	9.48×10^{4}	0.35	
			1.82×10^{5}	0.14	
			2.68×10^{5}	0.07	
			3.55×10^{5}	0.05	
60 % fb	3.46 – 3.53	1.02×10^{-4}	1.19×10^{3}	0.96	9.70×10^{-6}
		(p*D* 10.82)	1.25×10^{4}	0.87	
			1.93×10^{4}	0.82	
			7.93×10^{4}	0.46	
			9.33×10^{4}	0.39	
			1.06×10^{5}	0.36	
			1.64×10^{5}	0.21	
			1.82×10^{5}	0.17	
			1.92×10^{5}	0.16	
			2.52×10^{5}	0.08	
			2.67×10^{5}	0.08	
			3.38×10^{5}	0.07	
			3.66×10^{5}	0.06	
			6.03×10^{5}	0.00	
50 % fb	3.46 – 3.53	5.97×10^{-5}	1.07×10^{3}	0.97	6.39×10^{-6}
		(p*D* 10.59)	9.50×10^{4}	0.54	
			1.82×10^{5}	0.30	
			2.68×10^{5}	0.18	
			3.55×10^{5}	0.12	
			6.15×10^{5}	0.02	
35 % fb	3.46 – 3.53	4.27×10^{-5}	1.06×10^{3}	0.96	4.56×10^{-6}

		(p*D* 10.44)	9.75×10^{4}	0.64	
			1.85×10^{5}	0.44	
			2.76×10^{5}	0.28	
			3.68×10^{5}	0.19	
			6.23×10^{5}	0.05	
			7.08×10^{5}	0.04	
			7.86×10^{5}	0.03	
			8.75×10^{5}	0.01	
20 % fb	3.46 – 3.53	3.81×10^{-5}	1.06×10^{3}	0.96	4.44×10^{-6}
		(p*D* 10.39)	9.56×10^{4}	0.65	
			1.81×10^{5}	0.46	
			2.73×10^{5}	0.29	
			3.64×10^{5}	0.22	
			6.19×10^{5}	0.06	
			7.05×10^{5}	0.03	
22 % fb	3.46 – 3.53	1.91×10^{-5}	1.19×10^{3}	1.00	2.47×10^{-6}
		(p*D* 10.09)	1.81×10^{4}	0.92	
			7.80×10^{4}	0.82	
			1.63×10^{5}	0.55	
			2.56×10^{5}	0.68	
			3.37×10^{5}	0.43	
			6.01×10^{5}	0.22	
			6.83×10^{5}	0.18	
			7.76×10^{5}	0.14	
			8.56×10^{5}	0.12	
			9.42×10^{5}	0.10	
			1.22×10^{6}	0.02	

[a] fb = free base. [b] Calculated as $f(s) = 1 - f(p)$, where $f(p)$ is the fraction of product appearing.

Figures S1.4 and S1.5 show the dependence of k_{ex} on the concentration of deuteroxide for H_A in c(D-Pro-L-Pro). Second order rate constants for deuteroxide-catalysed-exchange of $k_{DO} = 9.69 \times 10^{-2}$ M^{-1} s^{-1} (*via* $H_D/H_{D'}$) and $k_{DO} = 9.31 \times 10^{-2}$ M^{-1} s^{-1} (*via* $H_4/H_{4'}$) are obtained from the slopes of linear fits of the reaction data to Eqn. S5. These k_{DO} values are in good agreement.

Figure S1.4: Plot of the dependence of k_{ex} upon [DO⁻] for the deuterium exchange of the prolyl α-protons H_A in c(D-Pro-L-Pro) (5.0 mM) monitored *via* $H_D/H_{D'}$ protons on c(D-Pro-L-Pro) in $KDCO_3$ (0.185 M, p*D* = 10.09 – 10.83) buffered D_2O solution, *I* = 1.0 (KCl) and 25 ºC.

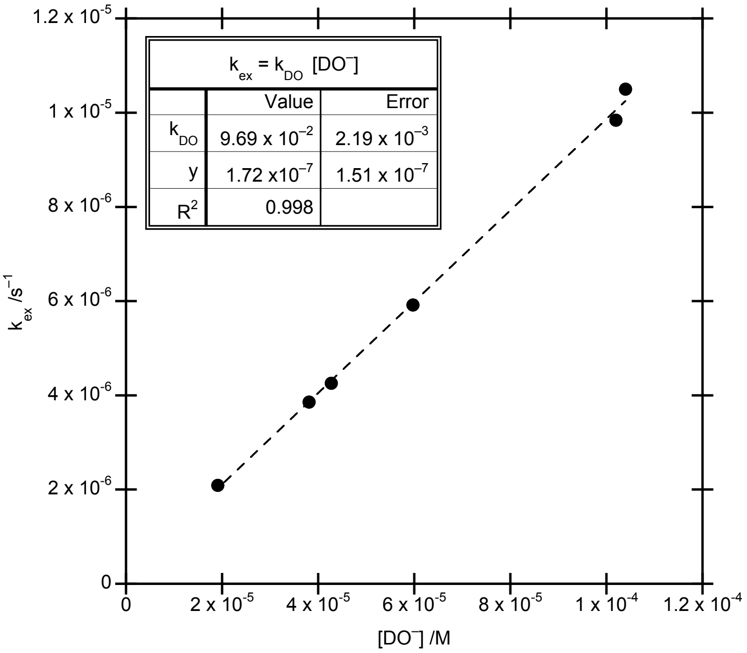

Figure S1.5: Plot of the dependence of k_{ex} upon [DO⁻] for the deuterium exchange of the prolyl α-protons H_A in c(D-Pro-L-Pro) (5.0 mM) monitored *via* $H_4/H_{4'}$ protons on c(D-Pro-D-Pro) / c(L-Pro-L-Pro) in $KDCO_3$ (0.185 M, p*D* = 10.09 – 10.83) buffered D_2O solution, *I* = 1.0 (KCl) and 25 ºC.

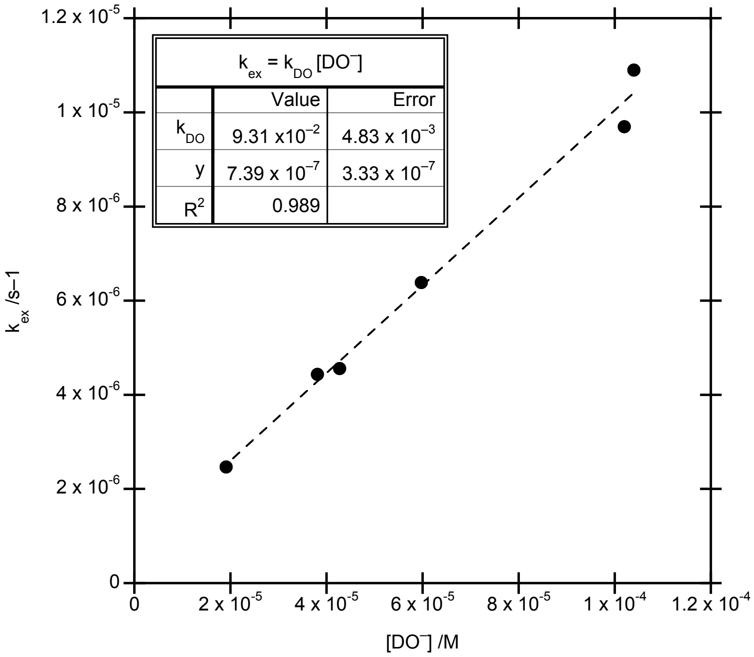

Figures S1.4 and S1.5 also indicate that buffer catalysis is insignificant as i) both fits intersect the y-axis close to the origin, and ii) no upwards curvature of reaction data is observed at higher concentrations of the free base form of the buffer [CO_3^{2-}].

For a more definitive assessment of the contribution of buffer catalysis, deuterium exchange reactions were undertaken in a series of solutions with an identical buffer ratio (to maintain a relatively constant p*D* and therefore a consistent contribution of k_{DO}[DO^-] to k_{ex}, Eqn. S6) and a varying total concentration of buffer, [$KDCO_3$].[iii] The exchange reactions were performed at 70 % free base $KDCO_3$ over the [$KDCO_3$] range 0.07 – 0.37 M. Ideally, the p*D* should remain constant as the concentration of the buffer changes, however, experimentally the p*D* varied unavoidably by 0.13 units. Therefore it was necessary to determine whether the relatively small differences in k_{ex} between solutions were due to changes in [CO_3^{2-}] or changes in [DO^-].

Figures S1.6 and S1.8 show the dependence of k_{ex} for $H_D/H_{D'}$ and $H_4/H_{4'}$ on CO_3^{2-} concentration (Tables S1.3 – S1.4) while Figures S1.7 and S1.9 show the dependence on deuteroxide concentration. In the former case, the fit of the data would give $k_{buff} = 1.64 \times 10^{-5} – 1.88 \times 10^{-5}$ M^{-1} s^{-1}. However, the fit of the data in the latter case gives $k_{DO} = 9.36 \times 10^{-2} – 1.05 \times 10^{-1}$ M^{-1} s^{-1} which are in good agreement with the k_{DO} values determined using Figures S1.4 and S1.5 above. Consequently, it appears more likely that the changes in k_{ex} observed in the experiments to assess buffer catalysis are due to the changes in deuteroxide concentration between the different concentrations of buffer rather than the buffer itself.

[iii] There are two possible bases in $KDCO_3$ buffer: DCO_3^- and CO_3^{2-}. Only CO_3^{2-} is considered in determining buffer catalysis because it is a significantly stronger base than DCO_3^- as pK_a (HCO_3^-) = 10.33 while pK_a (H_2CO_3) = 3.58.

Table S1.3: Reaction data and observed first order rate constants (k_{ex}) for the deuterium exchange of the prolyl α-protons H_A in c(D-Pro-L-Pro) (5.0 mM) monitored via $H_D/H_{D'}$ pyrrolidine protons on c(D-Pro-D-Pro) / c(L-Pro-L-Pro) in 70 % free base $KDCO_3$ (0.07 – 0.37 M, pD = 10.85 – 10.98) buffered D_2O solution, I = 1.0 (KCl) and 25 °C.

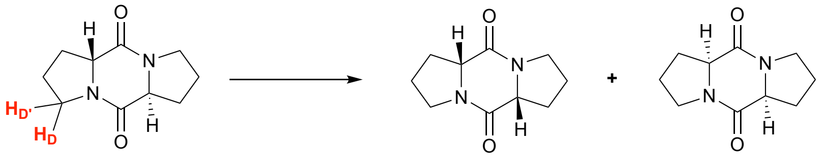

$[KDCO_3]$ /M	$[CO_3^{2-}]$ /M	$[DO^-]$ /M	Time /s	f(s)[a]	k_{ex} /s^{-1}
0.37	0.259	1.48×10^{-4}	1.06×10^{3}	1.00	1.39×10^{-5}
		(pD = 10.98)	7.38×10^{4}	0.43	
			8.85×10^{4}	0.24	
			1.68×10^{5}	0.11	
0.25	0.175	1.37×10^{-4}	1.06×10^{3}	1.00	1.32×10^{-5}
		(pD = 10.95)	5.02×10^{4}	0.53	
			8.91×10^{4}	0.32	
			1.43×10^{5}	0.16	
			2.62×10^{5}	0.04	
			3.07×10^{5}	0.03	
			5.66×10^{5}	0.00	
0.185	0.1295	1.29×10^{-4}	1.06×10^{3}	1.00	1.26×10^{-5}
		(pD = 10.92)	4.92×10^{4}	0.55	
			8.92×10^{4}	0.35	
			1.45×10^{5}	0.18	
			2.63×10^{5}	0.05	
			3.06×10^{5}	0.03	
			5.66×10^{5}	0.00	
0.13	0.091	1.22×10^{-4}	1.06×10^{3}	1.00	1.17×10^{-5}
		(pD = 10.90)	5.14×10^{4}	0.59	
			8.86×10^{4}	0.39	
			1.51×10^{5}	0.21	
			2.62×10^{5}	0.07	
			3.06×10^{5}	0.05	
			5.65×10^{5}	0.00	

0.07	0.049	1.09×10^{-4}	1.06×10^{3}	1.00	1.03×10^{-5}
		(pD = 10.85)	7.38×10^{4}	0.49	
			8.85×10^{4}	0.42	
			1.68×10^{5}	0.20	
			2.62×10^{5}	0.09	
			3.06×10^{5}	0.06	
			5.65×10^{5}	0.00	

[a] Calculated as f(s) = 1 – f(p), where f(p) is the fraction of product appearing.

Table S1.4: Reaction data and observed first order rate constants (k_{ex}) for the deuterium exchange of the prolyl α-protons H_A in c(D-Pro-L-Pro) (5.0 mM) monitored via H_4/$H_{4'}$ pyrrolidine protons on c(D-Pro-D-Pro) / c(L-Pro-L-Pro) in 70 % free base $KDCO_3$ (0.07 – 0.37 M, pD = 10.85 – 10.98) buffered D_2O solution, I = 1.0 (KCl) and 25 ºC.

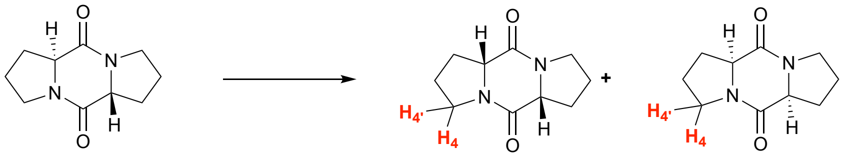

$[KDCO_3]$ /M	$[CO_3^{2-}]$ /M	$[DO^-]$ /M	Time /s	f(s)[a]	k_{ex} /s^{-1}
0.37	0.259	1.48×10^{-4}	1.06×10^{3}	0.97	1.39×10^{-5}
		(pD = 10.98)	7.38×10^{4}	0.41	
			8.85×10^{4}	0.23	
			1.68×10^{3}	0.10	
			2.62×10^{5}	0.02	
			3.06×10^{5}	0.02	
			5.65×10^{5}	0.00	
0.25	0.175	1.37×10^{-4}	1.06×10^{3}	0.97	1.32×10^{-5}
		(pD = 10.95)	5.02×10^{4}	0.51	
			8.91×10^{4}	0.31	
			1.43×10^{5}	0.15	
			2.62×10^{5}	0.02	
			3.07×10^{5}	0.03	
			5.66×10^{5}	0.00	
0.185	0.1295	1.29×10^{-4}	1.06×10^{3}	0.97	1.26×10^{-5}
		(pD = 10.92)	4.92×10^{4}	0.54	
			8.92×10^{4}	0.32	
			1.45×10^{5}	0.16	

			2.63×10^{5}	0.04	
			3.06×10^{5}	0.02	
			5.66×10^{5}	0.00	
0.13	0.091	1.22×10^{-4}	1.06×10^{3}	0.97	1.17×10^{-5}
		(pD = 10.90)	5.14×10^{4}	0.56	
			8.86×10^{4}	0.36	
			1.51×10^{5}	0.15	
			2.62×10^{5}	0.01	
			3.06×10^{5}	0.07	
			5.65×10^{5}	0.00	
0.07	0.049	1.09×10^{-4}	1.06×10^{3}	0.97	1.03×10^{-5}
		(pD = 10.85)	7.38×10^{4}	0.47	
			8.85×10^{4}	0.41	
			1.68×10^{5}	0.17	
			2.62×10^{5}	0.05	
			3.06×10^{5}	0.03	
			5.65×10^{5}	0.00	

[a] Calculated as f(s) = 1 – f(p), where f(p) is the fraction of product appearing.

Figure S1.6: Plot of the dependence of k_{ex} upon $[CO_3^{2-}]$ for the deuterium exchange of the prolyl α-protons H_A in c(D-Pro-L-Pro) (5.0 mM) monitored via $H_D/H_{D'}$ pyrrolidine protons on c(D-Pro-D-Pro) / c(L-Pro-L-Pro) in 70 % free base $KDCO_3$ (0.07 – 0.37 M, pD = 10.85 – 10.98) buffered D_2O solution, I = 1.0 (KCl) and 25 ºC.

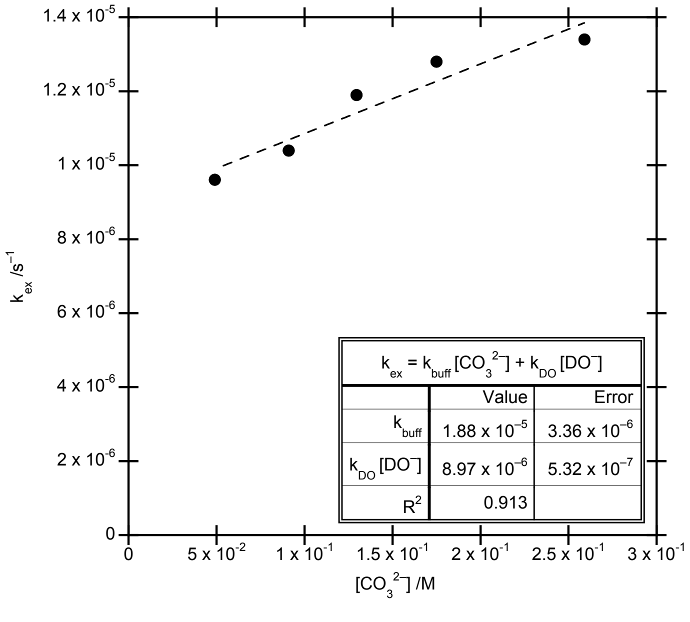

Figure S1.7: Plot of the dependence of k_{ex} upon $[DO^-]$ for the deuterium exchange of the prolyl α-protons H_A in c(D-Pro-L-Pro) (5.0 mM) monitored via $H_D/H_{D'}$ pyrrolidine protons on c(D-Pro-D-Pro) / c(L-Pro-L-Pro) in 70 % free base $KDCO_3$ (0.07 – 0.37 M, pD = 10.85 – 10.98) buffered D_2O solution, I = 1.0 (KCl) and 25 ºC.

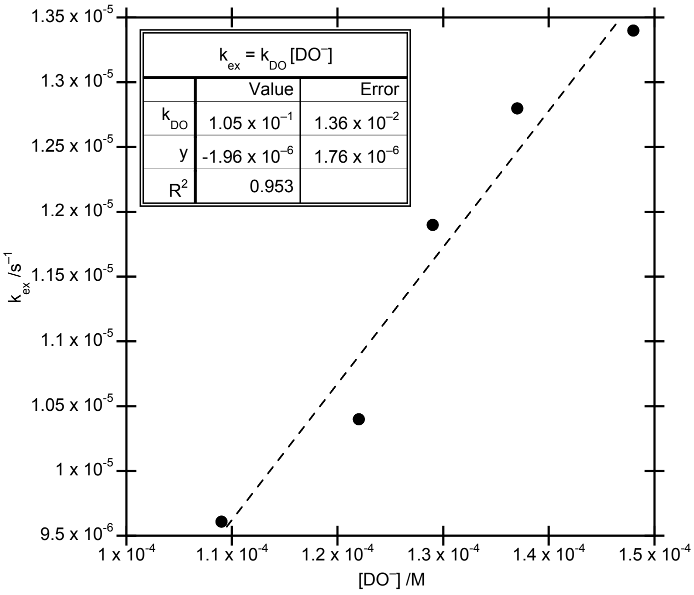

Figure S1.8: Plot of the dependence of k_{ex} upon $[CO_3^{2-}]$ for the deuterium exchange of the prolyl α-protons H_A in c(D-Pro-L-Pro) (5.0 mM) monitored via $H_4/H_{4'}$ pyrrolidine protons on c(D-Pro-D-Pro) / c(L-Pro-L-Pro) in 70 % free base $KDCO_3$ (0.07 – 0.37 M, pD = 10.85 – 10.98) buffered D_2O solution, I = 1.0 (KCl) and 25 ºC.

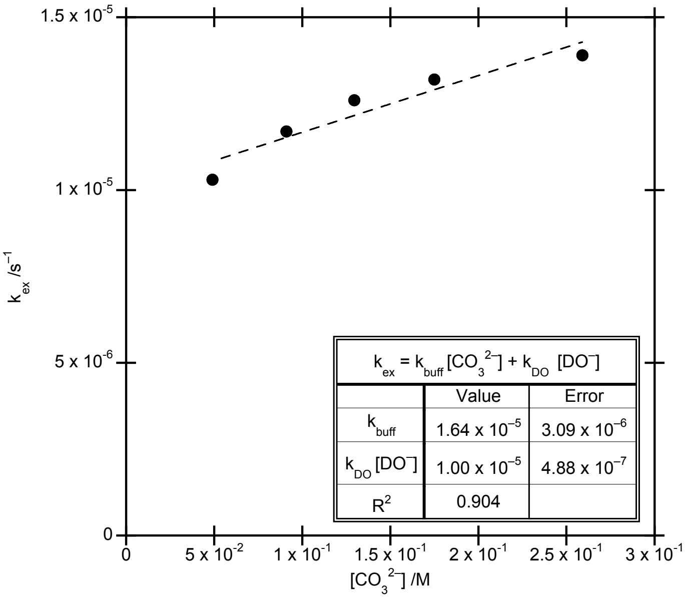

Figure S1.9: Plot of the dependence of k_{ex} upon $[DO^-]$ for the deuterium exchange of the prolyl α-protons H_A in c(D-Pro-L-Pro) (5.0 mM) monitored via $H_4/H_{4'}$ pyrrolidine protons on c(D-Pro-D-Pro) / c(L-Pro-L-Pro) in 70 % free base $KDCO_3$ (0.07 – 0.37 M, pD = 10.85 – 10.98) buffered D_2O solution, I = 1.0 (KCl) and 25 ºC.

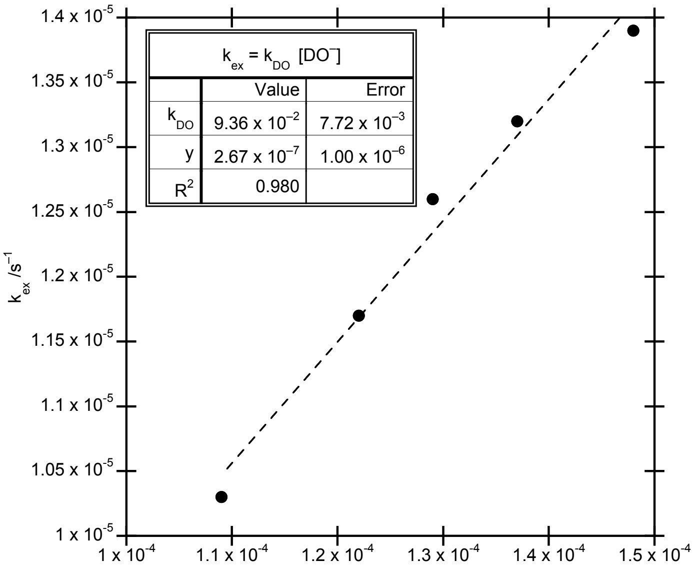

S1.4.6 Hydrogen-deuterium exchange reactions of c(D-Pro-D-Pro)

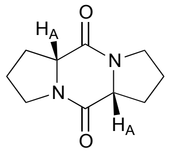

Pseudo first order rate constants for the deuteroxide catalysed exchange, k_{ex}, of the prolyl α-protons H_A of c(D-Pro-D-Pro) were determined in $KDCO_3$ buffered D_2O solutions (pD = 10.16 – 10.81) using ^{1}H NMR spectroscopy (400 MHz).

A representative set of spectra taken at three time points during the reaction at pD = 10.60 is shown in Figure S1.10. Deuterium exchange at the H_A position resulted in disappearance of the doublet of doublets due to H_A at 4.49 ppm over the course of the reaction. The level of exchange was monitored relative to a tetramethylammonium deuteriosulfate internal standard, whose methyl protons (3.21 ppm) are non-exchangeable. The disappearance of the doublet of doublets due to H_A was used to monitor the extent of exchange. No changes in the integrated areas from the pyrrolidine ring protons (H_B, $H_{B'}$, H_C, $H_{C'}$ H_D and $H_{D'}$) on c(D-Pro-D-Pro) were observed. The product of deuterium exchange will solely be deuterated c(D-Pro-D-Pro) (Scheme S3) as formation of c(D-Pro-L-Pro) is not favoured.[3] No hydrolysis of c(D-Pro-D-Pro) was observed.

Figure S1.10: Representative ^{1}H NMR spectra at 400 MHz for the deuterium exchange reaction c(D-Pro-D-Pro) (5.0 mM) in $KDCO_3$ (50% free base, pD = 10.60, 0.185 M), 25 °C and ionic strength I = 1.0 M (KCl).

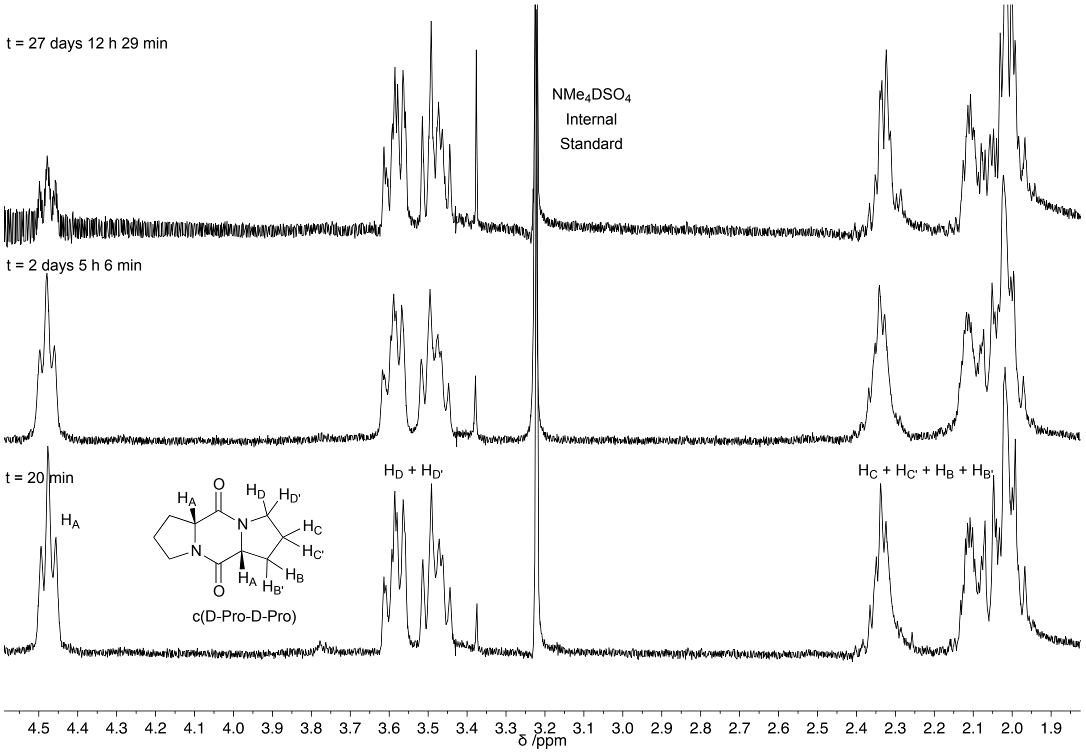

Scheme S3: Outcomes of the deuterium exchange reaction of c(D-Pro-D-Pro).

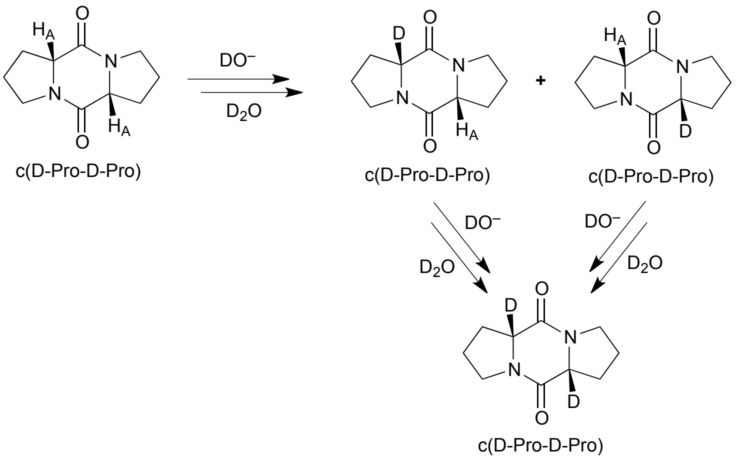

Experimentally observed first order rate constants for deuteroxide-catalysed deuterium exchange, k_{ex}, of prolyl α-protons H_A of c(D-Pro-D-Pro) were determined from the fit of the fraction of protonated substrate remaining, f(s), for H_A c(D-Pro-D-Pro) to Eqn. S3. Reaction data and k_{ex} values for H_A over the pD range pD = 10.16 – 10.81 are shown in Table S1.5.

Figure S1.11 shows the dependence of k_{ex} on the concentration of deuteroxide. A value of k_{DO} = 8.62 × 10^{-3} M^{-1} s^{-1} is obtained as the slope of a linear fit of the reaction data to Eqn. S5. Again, there was no indication of buffer catalysis of the deuterium exchange reaction of c(D-Pro-D-Pro) as the y-axis intercept is close to the origin, and no upwards curvature of reaction data is observed at higher concentrations of the free base form of the buffer [CO_3^{2-}].

Table S1.5: Reaction data and observed first order rate constants (k_{ex}) for the deuterium exchange of the prolyl α-protons H_A in c(D-Pro-D-Pro) (5.0 mM) in $KDCO_3$ (0.185 M, pD = 10.16 – 10.81) buffered D_2O solution, I = 1.0 (KCl) and 25 ºC.

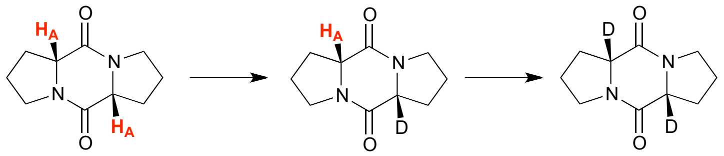

Experiment	Proton Chemical Shift /ppm	[DO⁻] /M	Time /s	f(s)[a]	k_{ex} /s^{-1}
70% fb	H_A	9.90 × 10^{-5}	1.19 × 10^{3}	1.00	8.80 × 10^{-7}
		(pD 10.81)	1.57 × 10^{4}	1.00	
			2.29 × 10^{4}	0.97	
			8.13 × 10^{4}	0.95	
			9.70 × 10^{4}	0.90	
			1.71 × 10^{5}	0.87	
			1.95 × 10^{5}	0.85	
			2.55 × 10^{5}	0.80	
			2.83 × 10^{5}	0.79	
			3.44 × 10^{5}	0.73	
			6.04 × 10^{5}	0.58	
50% fb	H_A	6.10 × 10^{-5}	1.19 × 10^{3}	1.00	5.67 × 10^{-7}
		(pD 10.60)	1.19 × 10^{4}	0.96	
			1.90 × 10^{4}	0.95	
			7.74 × 10^{4}	0.94	
			9.31 × 10^{4}	0.93	
			1.67 × 10^{5}	0.88	
			1.91 × 10^{5}	0.88	
			2.51 × 10^{5}	0.86	
			2.79 × 10^{5}	0.82	
			3.49 × 10^{5}	0.81	
			6.00 × 10^{5}	0.68	

			6.88×10^{5}	0.64	
			7.69×10^{5}	0.64	
			8.59×10^{5}	0.62	
			9.60×10^{5}	0.57	
			1.21×10^{6}	0.51	
			1.29×10^{6}	0.49	
			1.39×10^{6}	0.45	
			1.47×10^{6}	0.46	
			1.56×10^{6}	0.42	
			1.82×10^{6}	0.36	
			1.90×10^{6}	0.35	
			1.99×10^{6}	0.32	
			2.16×10^{6}	0.31	
			2.38×10^{6}	0.28	
20% fb	H_A	3.99×10^{-5}	1.19×10^{3}	1.00	4.13×10^{-7}
		(p*D* 10.41)	7.22×10^{4}	0.99	
			1.53×10^{5}	0.95	
			2.50×10^{5}	0.92	
			3.46×10^{5}	0.86	
			5.94×10^{5}	0.75	
			6.74×10^{5}	0.73	
			7.80×10^{5}	0.70	
			8.60×10^{5}	0.68	
			9.49×10^{5}	0.65	
			1.21×10^{6}	0.62	
			1.29×10^{6}	0.59	
			1.38×10^{6}	0.56	
			1.63×10^{6}	0.54	
			1.89×10^{6}	0.50	
22% fb	H_A	2.24×10^{-5}	1.19×10^{3}	1.00	2.60×10^{-7}
		(p*D* 10.16)	7.22×10^{4}	0.99	
			1.55×10^{5}	0.98	
			2.62×10^{5}	0.93	
			3.47×10^{5}	0.87	
			5.94×10^{5}	0.82	
			6.76×10^{5}	0.81	
			7.80×10^{5}	0.80	
			8.61×10^{5}	0.79	

1.21×10^6	0.75
1.29×10^6	0.71
1.38×10^6	0.69
1.54×10^6	0.68
1.81×10^6	0.66

[a] fraction of unreacted substrate remaining at each time point.

Figure S1.11: Plot of the dependence of k_{ex} upon $[DO^-]$ for the deuterium exchange of the prolyl α-protons H_A in c(D-Pro-D-Pro) (5.0 mM) in $KDCO_3$ (0.185 M, pD = 10.16 – 10.81) buffered D_2O solution, I = 1.0 (KCl) and 25 ºC.

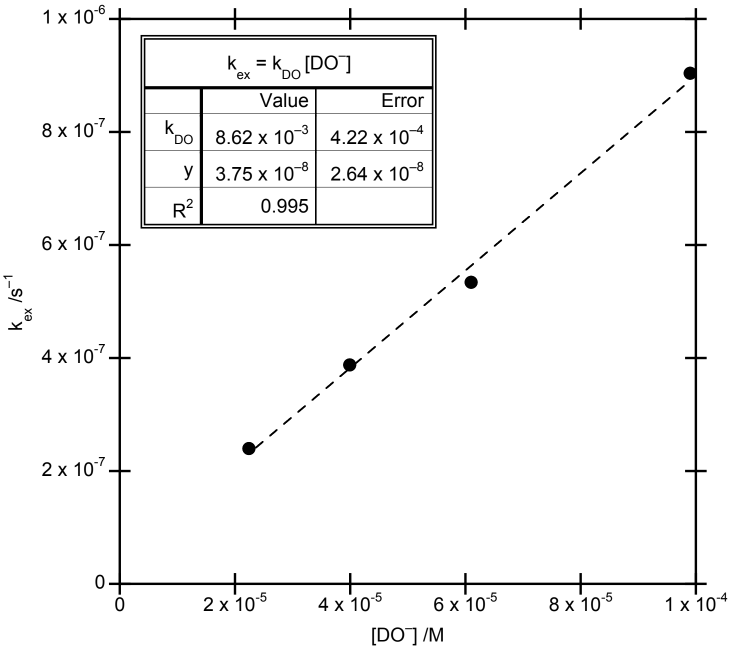

S1.4.7 Hydrogen-deuterium exchange reactions of c(D-Ala-L-Pro)

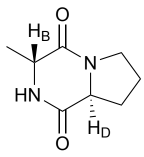

Pseudo first order rate constants for the deuteroxide catalysed exchange, k_{ex}, of the prolyl α-proton H_D and alanyl α-proton H_B of c(D-Ala-L-Pro) were determined in $KDCO_3$ buffered D_2O solutions (pD = 10.12 – 10.98) using ^{1}H NMR spectroscopy (400 MHz).

S1.4.7.1 c(D-Ala-L-Pro) Prolyl α-proton

A representative set of spectra taken at three time points during the reaction at p*D* = 10.58 is shown in Figure S1.12. Deuterium exchange at the Pro H_D position on c(D-Ala-L-Pro) resulted in disappearance of the doublet of doublets due to H_D at 4.30 ppm over the course of the reaction. The level of exchange was monitored relative to a tetramethylammonium deuteriosulfate internal standard, whose methyl protons (singlet, 3.14 ppm) are non-exchangeable. The products of deuterium exchange at the H_D position on c(D-Ala-L-Pro) are deuterated *trans*-c(D-Ala-L-Pro) and *cis*-c(D-Ala-D-Pro) (Scheme S4). Previous literature studies report an almost equal distribution (52% *trans* : 48% *cis*) at equilibrium in aqueous solution.[3] Consequently, ^{1}H NMR spectra over the course of the reaction showed the disappearance of the doublet corresponding to the alanyl methyl group (H_A) and the multiplet owing to prolyl pyrrolidine ring protons (H_E, $H_{E'}$, H_F, $H_{F'}$ H_G and $H_{G'}$) on c(D-Ala-L-Pro), and the appearance of the doublet for the analogous methyl (H_1) and the multiplets for the analogous pyrrolidine ring protons (H_5, $H_{5'}$, H_6, $H_{6'}$ H_7 and $H_{7'}$) on c(D-Ala-D-Pro) (Figure S1.12). A minor amount of hydrolysis product (< 10 % after 11 days) was also observed in the ^{1}H NMR spectra.

Figure S1.12: Representative ^{1}H NMR spectra at 400 MHz for the deuterium exchange reaction of c(D-Ala-L-Pro) (5.0 mM) in $KDCO_3$ (50% free base, pD = 10.58, 0.185 M), 25 °C and ionic strength I = 1.0 M (KCl).

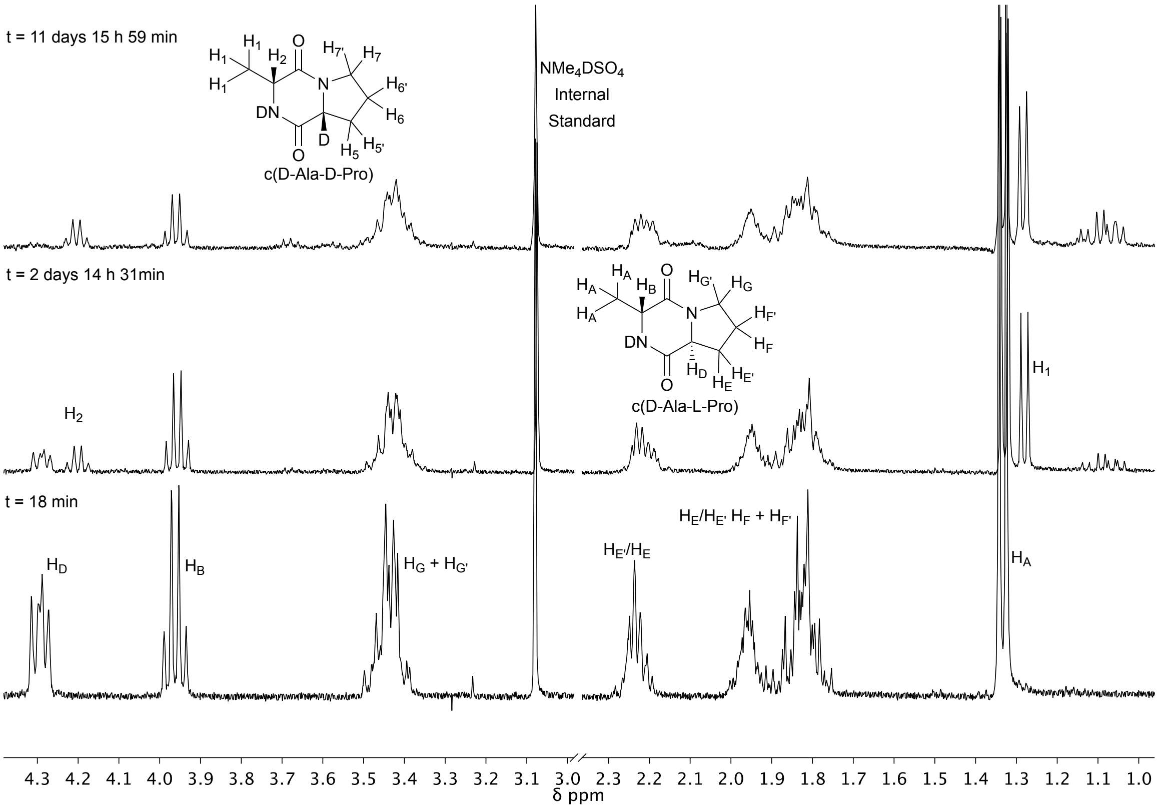

Scheme S4: Outcomes of the deuterium exchange reactions of c(D-Ala-L-Pro).

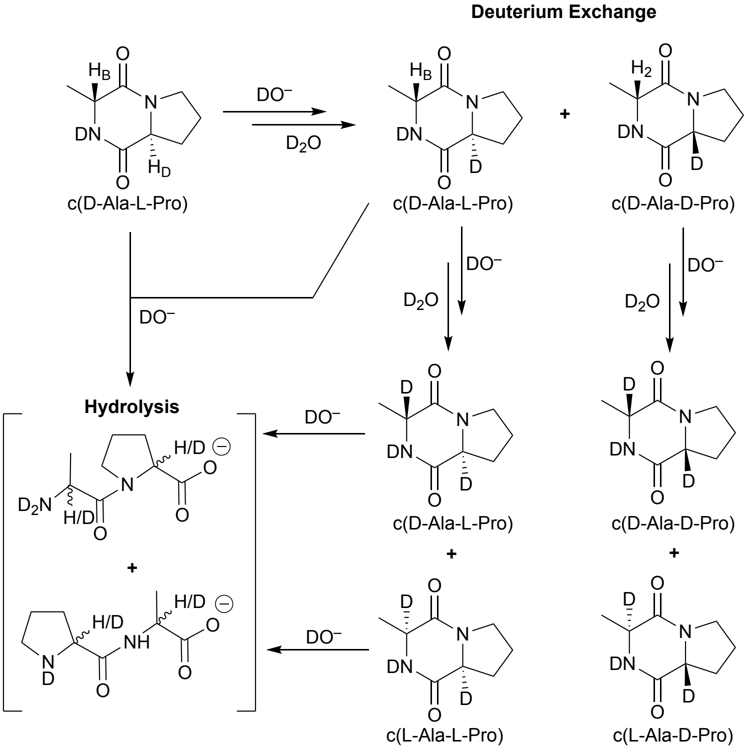

Pseudo first order rate constants for exchange were determined by monitoring the disappearance of signal from the prolyl proton H_D (4.30 ppm) on c(D-Ala-L-Pro) and the appearance of the methyl protons H_1 (1.29 ppm) on the product c(D-Ala-D-Pro) (Figure S1.12)[iv]. The rate limiting step for formation of c(D-Ala-D-Pro) will be deprotonation of c(D-Ala-L-Pro) and therefore H_1 provides an alternative assessment for k_{ex}. Experimentally observed first order rate constants for deuteroxide catalysed deuterium exchange, k_{ex}, of prolyl α-protons H_A of c(D-Ala-L-Pro) were determined from the fit of the fraction of protonated substrate remaining, f(s), for H_D c(D-Ala-L-Pro) and the fit of the fraction of deuterated product appearing, f(p) for H_1 c(D-Ala-D-Pro) to Eqn. S3. Reaction data and k_{ex} values for H_D and H_1 over the pD range pD = 10.12 – 10.98 are shown below in Table S1.6 and Table S1.7, respectively.

Figures S1.13 and S1.14 show the dependence of k_{ex} on the concentration of deuteroxide for H_D and H_1 (Table S1.6 and Table S1.7). Values of $k_{DO} = 9.45 \times 10^{-2}$ M^{-1} s^{-1} for H_D and $k_{DO} = 1.18 \times 10^{-1}$ M^{-1} s^{-1} for H_1 are obtained as the slope of a linear fit of the reaction data to Eqn. S5, which are in good agreement.

[iv] Note that as the while the H_1 signal represent three protons there is no need to divide the k_{DO} value by three as these protons do not undergo deuterium exchange.

Table S1.6: Reaction data and observed first order rate constants (k_{ex}) for the deuterium exchange of the prolyl α-protons H_D in c(D-Ala-L-Pro) (5.0 mM) in $KDCO_3$ (0.185 M, pD = 10.12 – 10.98) buffered D_2O solution, I = 1.0 (KCl) and 25 ºC.

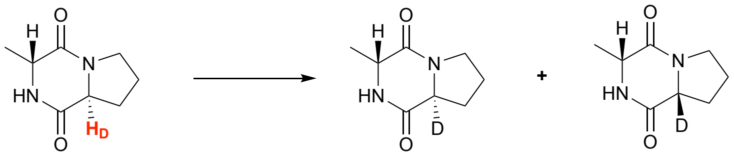

Experiment	Proton Chemical Shift /ppm	[DO⁻] /M	Time /s	f(s)[a]	k_{ex} /s^{-1}
70%	4.26 – 4.33	9.90×10^{-5}	5.40×10^{2}	1.00	8.57×10^{-6}
	H_D	(pD 10.81)	1.31×10^{4}	0.88	
			2.38×10^{4}	0.83	
			8.09×10^{4}	0.49	
			9.32×10^{4}	0.44	
			1.07×10^{5}	0.41	
			1.71×10^{5}	0.23	
			1.94×10^{5}	0.18	
			3.63×10^{5}	0.06	
			4.44×10^{5}	0.04	
60%	4.26 – 4.33	9.79×10^{-5}	1.06×10^{3}	1.00	9.46×10^{-6}
	H_D	(pD 10.80)	1.46×10^{4}	0.86	
			7.75×10^{4}	0.46	
			1.01×10^{5}	0.36	
			1.64×10^{5}	0.21	
			2.52×10^{5}	0.12	
			2.72×10^{5}	0.08	
			3.35×10^{5}	0.09	
			3.58×10^{5}	0.08	
			5.97×10^{5}	0.05	
			7.68×10^{5}	0.08	
50%fb	4.26 – 4.33	5.90×10^{-5}	1.07×10^{3}	1.00	4.62×10^{-6}
	H_D	(pD 10.58)	5.10×10^{4}	0.77	
			1.38×10^{5}	0.51	
			2.25×10^{5}	0.32	
			4.01×10^{5}	0.15	
			4.85×10^{5}	0.13	
			5.73×10^{5}	0.08	
			6.59×10^{5}	0.09	

			7.45×10^5	0.08	
			8.40×10^5	0.05	
			1.01×10^6	0.06	
			1.09×10^6	0.05	
			1.19×10^6	0.04	
20%	4.26 – 4.33	4.27×10^{-5}	1.06×10^3	1.00	3.42×10^{-6}
	H_D	(p*D* 10.44)	7.49×10^4	0.77	
			3.29×10^5	0.33	
			4.04×10^5	0.22	
			4.89×10^5	0.19	
			5.81×10^5	0.14	
			6.70×10^5	0.12	
			9.44×10^5	0.07	
			1.02×10^6	0.04	
			1.11×10^6	0.03	
			1.19×10^6	0.03	
35%	4.26 – 4.33	3.24×10^{-5}	1.06×10^3	1.00	2.70×10^{-6}
	H_D	(p*D* 10.32)	8.42×10^4	0.80	
			1.53×10^5	0.68	
			4.07×10^5	0.34	
			4.89×10^5	0.24	
			5.73×10^5	0.19	
			6.65×10^5	0.16	
			7.51×10^5	0.14	
			1.02×10^6	0.08	
			1.10×10^6	0.09	
			1.18×10^6	0.03	
			1.27×10^6	0.08	
22%fb	4.26 – 4.33	2.04×10^{-5}	1.19×10^3	1.00	1.59×10^{-6}
	H_D	(p*D* 10.12)	1.60×10^4	0.98	
			1.01×10^5	0.87	
			1.57×10^5	0.80	
			2.44×10^5	0.68	
			3.30×10^5	0.57	
			5.91×10^5	0.38	
			6.77×10^5	0.33	
			8.50×10^5	0.26	

9.35×10^{5}	0.20
1.19×10^{6}	0.15
1.29×10^{6}	0.13
1.37×10^{6}	0.12
1.46×10^{6}	0.10
1.54×10^{6}	0.09
1.81×10^{6}	0.08
1.89×10^{6}	0.09
1.98×10^{6}	0.09

[a] fraction of unreacted substrate remaining at each time point.

Table S1.7: Reaction data and observed first order rate constants (k_{ex}) for the deuterium exchange of the prolyl α-protons H_D in c(D-Ala-L-Pro) (5.0 mM) monitored via H_1 methyl protons on c(D-Ala-D-Pro) in $KDCO_3$ (0.185 M, pD = 10.12 – 10.98) buffered D_2O solution, I = 1.0 (KCl) and 25 ºC.

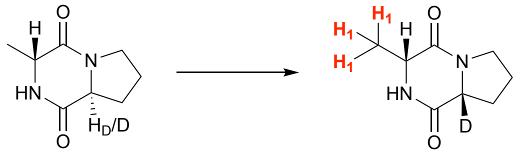

Experiment	Proton Chemical Shift /ppm	$[DO^{-}]$ /M	Time /s	f(s)[a]	k_{ex} /s^{-1}
70%	1.27 – 1.31	9.90×10^{-5}	5.40×10^{2}	1.00	1.07×10^{-5}
	H_1	(pD 10.81)	1.31×10^{4}	0.86	
			2.38×10^{4}	0.75	
			8.09×10^{4}	0.43	
			9.32×10^{4}	0.39	
			1.07×10^{5}	0.32	
			1.71×10^{5}	0.15	
			1.94×10^{5}	0.13	
			3.63×10^{5}	0.01	
			4.44×10^{5}	0.00	
60%	1.27 – 1.31	9.79×10^{-5}	1.06×10^{3}	1.00	1.24×10^{-5}
	H_1	(pD 10.80)	1.46×10^{4}	0.82	
			7.75×10^{4}	0.38	
			1.01×10^{5}	0.29	
			1.64×10^{5}	0.14	
			2.52×10^{5}	0.03	
			2.72×10^{5}	0.02	

			3.35×10^{5}	0.00	
50%fb	1.27 – 1.31	5.90×10^{-5}	1.07×10^{3}	1.00	6.26×10^{-6}
	H_1	(pD 10.58)	5.10×10^{4}	0.71	
			1.38×10^{5}	0.44	
			2.25×10^{5}	0.25	
			4.01×10^{5}	0.08	
			4.85×10^{5}	0.02	
			5.73×10^{5}	0.00	
			6.59×10^{5}	0.00	
			7.45×10^{5}	0.01	
			8.40×10^{5}	-0.02	
			1.01×10^{6}	0.02	
			1.09×10^{6}	0.03	
			1.19×10^{6}	0.04	
20%	1.27 – 1.31	4.27×10^{-5}	1.06×10^{3}	1.00	4.37×10^{-6}
	H_1	(pD 10.44)	7.49×10^{4}	0.71	
			3.29×10^{5}	0.24	
			4.04×10^{5}	0.18	
			4.89×10^{5}	0.11	
			5.81×10^{5}	0.08	
			6.70×10^{5}	0.04	
			9.44×10^{5}	0.02	
			1.02×10^{6}	0.01	
			1.11×10^{6}	0.02	
			1.19×10^{6}	0.00	
35%	1.27 – 1.31	3.24×10^{-5}	1.06×10^{3}	1.00	3.60×10^{-6}
	H_1	(pD 10.32)	8.42×10^{4}	0.72	
			1.53×10^{5}	0.58	
			4.07×10^{5}	0.23	
			4.89×10^{5}	0.19	
			5.73×10^{5}	0.13	
			6.65×10^{5}	0.09	
			7.51×10^{5}	0.05	
			1.02×10^{6}	0.04	
			1.10×10^{6}	0.02	
			1.18×10^{6}	0.01	
			1.27×10^{6}	0.00	

22%fb	1.27 – 1.31	2.04×10^{-5}	1.19×10^{3}	1.00	2.23×10^{-6}
	H_1	(p*D* 10.12)	1.60×10^{4}	0.94	
			1.01×10^{5}	0.76	
			1.57×10^{5}	0.66	
			2.44×10^{5}	0.58	
			3.30×10^{5}	0.48	
			5.91×10^{5}	0.28	
			6.77×10^{5}	0.23	
			8.50×10^{5}	0.14	
			9.35×10^{5}	0.16	
			1.19×10^{6}	0.07	
			1.29×10^{6}	0.07	
			1.37×10^{6}	0.06	
			1.46×10^{6}	0.04	
			1.54×10^{6}	0.03	
			1.81×10^{6}	0.01	
			1.89×10^{6}	0.03	
			1.98×10^{6}	0.00	

[a] Calculated as $f(s) = 1 - f(p)$, where $f(p)$ is the fraction of product appearing.

Figure S1.13: Plot of the dependence of k_{ex} upon $[DO^-]$ for the deuterium exchange of the prolyl α-proton H_D in c(D-Ala-L-Pro) (5.0 mM) in $KDCO_3$ (0.185 M, pD = 10.12 – 10.98) buffered D_2O solution, I = 1.0 (KCl) and 25 ºC.

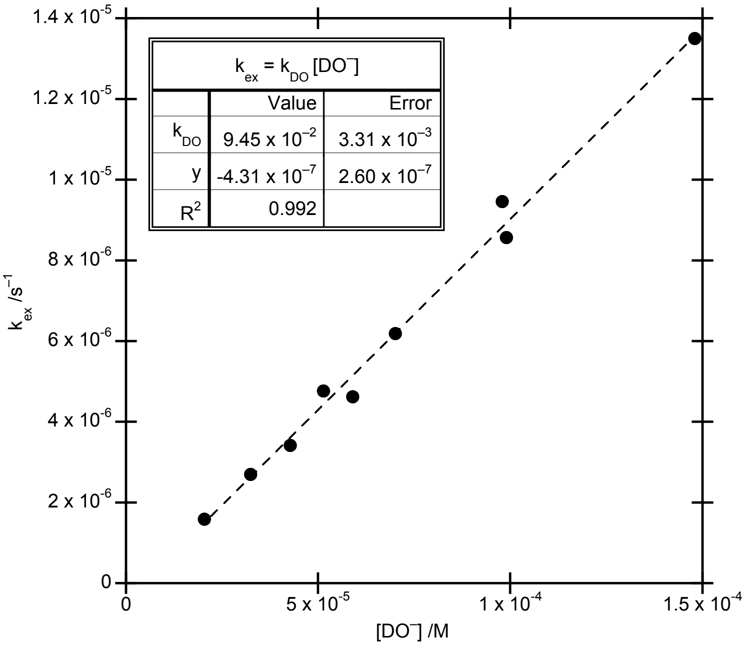

Figure S1.14: Plot of the dependence of k_{ex} upon $[DO^-]$ for the deuterium exchange of the prolyl α-proton H_D in c(D-Ala-L-Pro) (5.0 mM) monitored via H_1 methyl protons on c(D-Ala-D-Pro) in $KDCO_3$ (0.185 M, pD = 10.12 – 10.98) buffered D_2O solution, I = 1.0 (KCl) and 25 ºC.

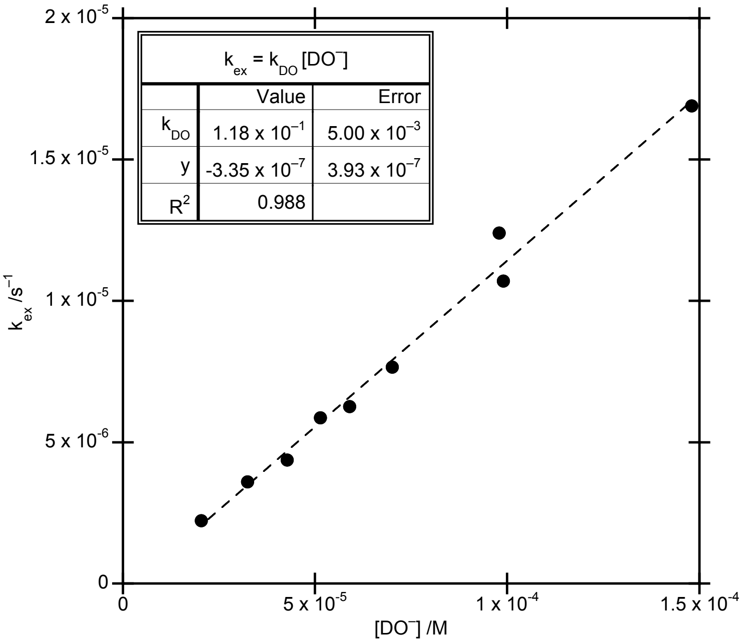

Analysis of Figures S1.13 and S1.14 indicates that there is insignificant buffer catalysis of hydrogen-deuterium exchange of the prolyl α-proton of c(D-Ala-L-Pro) as i) both fits intersect the y-axis close to the origin, and ii) no upwards curvature at higher concentrations of free base form of the buffer $[CO_3^{2-}]$ is observed. To further probe the role of buffer species, exchange reactions were undertaken in a series of solutions with an identical buffer ratio and a varying total concentration of buffer, $[KDCO_3]$. The H / D exchange reactions were performed at both 20 % free base and 70 % free base $KDCO_3$ over the $[KDCO_3]$ range 0.07 – 0.37 M (Tables S1.8 – S1.11). Experimentally, the pD varied by 0.07 – 0.14 units upon dilution at constant ionic strength. As before, it was necessary to ascertain whether the differences in k_{ex} between solutions were due to changes in $[CO_3^{2-}]$ or changes in $[DO^-]$. Figure S1.15, Figure S1.17, Figure S1.19 and Figure S1.21 show the dependence of k_{ex} on the $[CO_3^{2-}]$ while Figure S1.16, Figure S1.18, Figure S1.20 and Figure S1.22 show the dependence of k_{ex} on the $[DO^-]$. In the former four Figures the fits of the data would give $k_{buff} = 1.97 \times 10^{-5} – 2.93 \times 10^{-5}$ M^{-1} s^{-1}. However, the fits of the data in the latter four Figures gives $k_{DO} = 1.02 \times 10^{-1} – 1.54 \times 10^{-1}$ M^{-1} s^{-1} which are in good agreement with the k_{DO} values determined in Figures S1.13 and

S1.14 above. Consequently, it appears more likely that the observed changes in k_{ex} are due to the changes in [DO$^-$] rather than [CO_3^{2-}].

Table S1.8: Reaction data and observed first order rate constants (k_{ex}) for the deuterium exchange of the prolyl α-protons H_D in c(D-Ala-L-Pro) (5.0 mM) in 70 % free base $KDCO_3$ (0.07 – 0.37 M, pD = 10.85 – 10.98) buffered D_2O solutions, I = 1.0 (KCl) and 25 ºC.

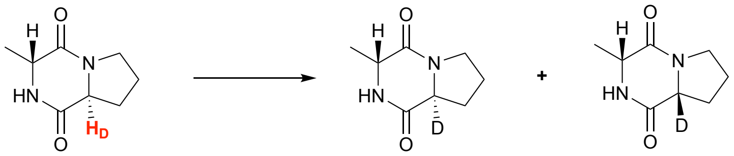

[$KDCO_3$] /M	[CO_3^{2-}] /M	[DO$^-$] /M	Time /s	f(s)[a]	k_{ex} /s^{-1}
0.37	0.259	1.48×10^{-4}	1.06×10^{3}	1.00	1.35×10^{-5}
		(pD 10.98)	2.43×10^{4}	0.72	
			8.10×10^{4}	0.33	
			1.11×10^{5}	0.23	
			2.68×10^{5}	0.04	
			3.41×10^{5}	0.00	
0.25	0.175	1.37×10^{-4}	1.06×10^{3}	1.00	1.23×10^{-5}
		(pD 10.95)	2.01×10^{4}	0.78	
			7.29×10^{4}	0.38	
			1.03×10^{5}	0.28	
			2.60×10^{5}	0.05	
			3.33×10^{5}	0.06	
			3.67×10^{5}	0.10	
			4.43×10^{5}	0.05	
			5.19×10^{5}	0.05	
			6.07×10^{5}	0.08	
			6.91×10^{5}	0.04	
			8.68×10^{5}	0.06	
0.185	0.1295	1.29×10^{-4}	1.06×10^{3}	1.00	9.68×10^{-6}
		(pD 10.92)	2.44×10^{4}	0.77	
			9.00×10^{4}	0.42	
			1.20×10^{5}	0.30	
			2.77×10^{5}	0.08	
			3.57×10^{5}	0.06	
			3.84×10^{5}	0.04	

			5.19×10^5	0.04	
			6.30×10^5	0.05	
			7.09×10^5	0.03	
			8.86×10^5	0.04	
0.13	0.091	1.22×10^{-4}	1.06×10^3	1.00	9.79×10^{-6}
		(p*D* 10.90)	2.30×10^4	0.81	
			8.33×10^4	0.41	
			1.12×10^5	0.32	
			2.69×10^5	0.10	
			3.49×10^5	0.08	
			4.22×10^5	0.03	
			5.11×10^5	0.04	
			6.28×10^5	0.06	
			7.01×10^5	0.04	
			8.77×10^5	0.04	
0.07	0.049	1.09×10^{-4}	1.06×10^3	1.00	8.94×10^{-6}
		(p*D* 10.85)	1.89×10^4	0.84	
			7.94×10^4	0.48	
			1.06×10^5	0.39	
			2.62×10^5	0.11	
			3.58×10^5	0.05	
			4.16×10^5	0.03	
			5.05×10^5	0.02	
			6.23×10^5	0.02	
			6.96×10^5	0.04	
			8.71×10^5	0.02	

[a] fraction of unreacted substrate remaining at each time point.

Table S1.9: Reaction data and observed first order rate constants (k_{ex}) for the deuterium exchange of the prolyl α-protons H_D in c(D-Ala-L-Pro) (5.0 mM) in 20 % free base $KDCO_3$ (0.07 – 0.37 M, pD = 10.59 – 10.66) buffered D_2O solutions, I = 1.0 (KCl) and 25 °C.

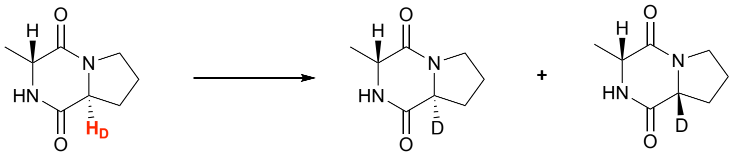

[$KDCO_3$] /M	[CO_3^{2-}] /M	[DO^-] /M	Time /s	f(s)[a]	k_{ex} /s^{-1}
0.37	0.074	7.01×10^{-5}	1.06×10^{3}	1.00	6.19×10^{-6}
		(pD 10.66)	7.13×10^{4}	0.63	
			1.57×10^{5}	0.38	
			2.41×10^{5}	0.22	
			3.28×10^{5}	0.14	
			6.75×10^{5}	0.04	
			7.62×10^{5}	0.05	
			8.48×10^{5}	0.05	
0.25	0.05	6.85×10^{-5}	1.06×10^{3}	1.00	5.67×10^{-6}
		(pD 10.65)	7.11×10^{4}	0.67	
			1.56×10^{5}	0.40	
			2.41×10^{5}	0.24	
			3.28×10^{5}	0.16	
			6.94×10^{5}	0.04	
			7.62×10^{5}	0.05	
			8.50×10^{5}	0.06	
0.185	0.037	6.25×10^{-5}	1.06×10^{3}	1.00	5.25×10^{-6}
		(pD 10.61)	7.10×10^{4}	0.67	
			1.59×10^{5}	0.44	
			2.41×10^{5}	0.26	
			3.28×10^{5}	0.18	
			6.94×10^{5}	0.05	
			7.64×10^{5}	0.06	
			8.50×10^{5}	0.05	
0.13	0.026	6.25×10^{-5}	1.06×10^{3}	1.00	5.39×10^{-6}
		(pD 10.61)	7.10×10^{4}	0.67	
			1.62×10^{5}	0.41	
			2.42×10^{5}	0.27	

			3.28×10^5	0.16	
			6.94×10^5	0.06	
			7.65×10^5	0.06	
			8.50×10^5	0.05	
0.07	0.014	5.97×10^{-5}	1.06×10^3	1.00	4.94×10^{-6}
		(p*D* 10.59)	7.02×10^4	0.70	
			1.60×10^5	0.44	
			2.39×10^5	0.30	
			3.25×10^5	0.20	
			6.91×10^5	0.05	
			7.62×10^5	0.05	
			8.49×10^5	0.06	

[a] fraction of unreacted substrate remaining at each time point.

Table S1.10: Reaction data and observed first order rate constants (k_{ex}) for the deuterium exchange of the prolyl α-protons H_D in c(D-Ala-L-Pro) (5.0 mM) monitored via H_1 methyl protons on c(D-Ala-D-Pro) in 70 % free base $KDCO_3$ (0.07 – 0.37 M, p*D* = 10.85 – 10.98) buffered D_2O solution, *I* = 1.0 (KCl) and 25 ºC.

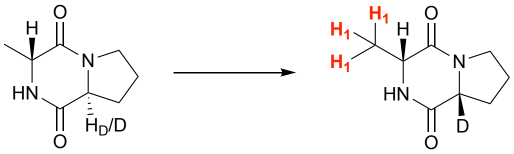

$[KDCO_3]$ /M	$[CO_3^{2-}]$ /M	$[DO^-]$ /M	Time /s	*f*(s)[a]	k_{ex} /s^{-1}
0.37	0.259	1.48×10^{-4}	1.06×10^3	1.00	1.69×10^{-5}
		(p*D* 10.98)	2.43×10^4	0.64	
			8.10×10^4	0.27	
			1.11×10^5	0.15	
			2.68×10^5	0.00	
0.25	0.175	1.37×10^{-4}	1.06×10^3	1.00	1.43×10^{-5}
		(p*D* 10.95)	2.01×10^4	0.72	
			7.29×10^4	0.37	
			1.03×10^5	0.23	
			2.60×10^5	0.00	
0.185	0.1295	1.29×10^{-4}	1.06×10^3	1.00	1.22×10^{-5}
		(p*D* 10.92)	2.44×10^4	0.72	
			9.00×10^4	0.34	

			1.20×10^5	0.25	
			2.77×10^5	0.01	
			3.57×10^5	0.02	
			3.84×10^5	0.00	
0.13	0.091	1.22×10^{-4}	1.06×10^3	1.00	1.18×10^{-5}
		(p*D* 10.90)	2.30×10^4	0.74	
			8.33×10^4	0.38	
			1.12×10^5	0.28	
			2.69×10^5	0.03	
			3.49×10^5	0.00	
			4.22×10^5	0.00	
0.07	0.049	1.09×10^{-4}	1.06×10^3	1.00	1.09×10^{-5}
		(p*D* 10.85)	1.89×10^4	0.81	
			7.94×10^4	0.43	
			1.06×10^5	0.33	
			2.62×10^5	0.04	
			3.58×10^5	0.01	
			4.16×10^5	0.00	
			5.05×10^5	0.00	

[a] Calculated as $f(s) = 1 - f(p)$, where $f(p)$ is the fraction of product appearing.

Table S1.11: Reaction data and observed first order rate constants (k_{ex}) for the deuterium exchange of the prolyl α-protons H_D in c(D-Ala-L-Pro) (5.0 mM) monitored via H_1 methyl protons on c(D-Ala-D-Pro) in 20 % free base $KDCO_3$ (0.07 – 0.37 M, p*D* = 10.59 – 10.66) buffered D_2O solution, *I* = 1.0 (KCl) and 25 ºC.

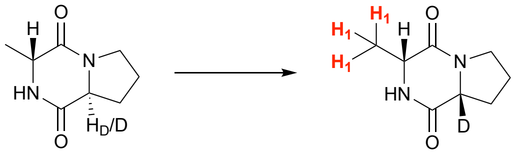

$[KDCO_3]$ /M	$[CO_3^{2-}]$ /M	$[DO^-]$ /M	Time /s	$f(s)^a$	k_{ex} /s^{-1}
0.37	0.074	7.01×10^{-5}	1.06×10^3	1.00	7.66×10^{-6}
		(p*D* 10.66)	7.13×10^4	0.57	
			1.57×10^5	0.31	
			2.41×10^5	0.15	
			3.28×10^5	0.08	
			6.75×10^5	0.00	

0.25	0.05	6.85×10^{-5}	1.06×10^{3}	1.00	7.20×10^{-6}
		(p*D* 10.65)	7.11×10^{4}	0.58	
			1.56×10^{5}	0.34	
			2.41×10^{5}	0.18	
			3.28×10^{5}	0.09	
			6.94×10^{5}	0.01	
			7.62×10^{5}	0.00	
0.185	0.037	6.25×10^{-5}	1.06×10^{3}	1.00	6.90×10^{-6}
		(p*D* 10.61)	7.10×10^{4}	0.57	
			1.59×10^{5}	0.36	
			2.41×10^{5}	0.20	
			3.28×10^{5}	0.11	
			6.94×10^{5}	0.02	
			7.64×10^{5}	0.01	
			8.50×10^{5}	0.00	
0.13	0.026	6.25×10^{-5}	1.06×10^{3}	1.00	6.35×10^{-6}
		(p*D* 10.61)	7.10×10^{4}	0.62	
			1.62×10^{5}	0.35	
			2.42×10^{5}	0.22	
			3.28×10^{5}	0.15	
			6.94×10^{5}	0.00	
0.07	0.014	5.97×10^{-5}	1.06×10^{3}	1.00	6.10×10^{-6}
		(p*D* 10.59)	7.02×10^{4}	0.64	
			1.60×10^{5}	0.38	
			2.39×10^{5}	0.23	
			3.25×10^{5}	0.14	
			6.91×10^{5}	0.02	
			7.62×10^{5}	0.01	
			8.49×10^{5}	0.00	

[a] Calculated as $f(s) = 1 - f(p)$, where $f(p)$ is the fraction of product appearing.

Figure S1.15: Plot of the dependence of k_{ex} upon $[CO_3^{2-}]$ for the deuterium exchange of the prolyl α-proton H_D in c(D-Ala-L-Pro) (5.0 mM) in $KDCO_3$ (70 % free base, 0.07 – 0.37 M, pD = 10.85 – 10.98) buffered D_2O solution, I = 1.0 (KCl) and 25 ºC.

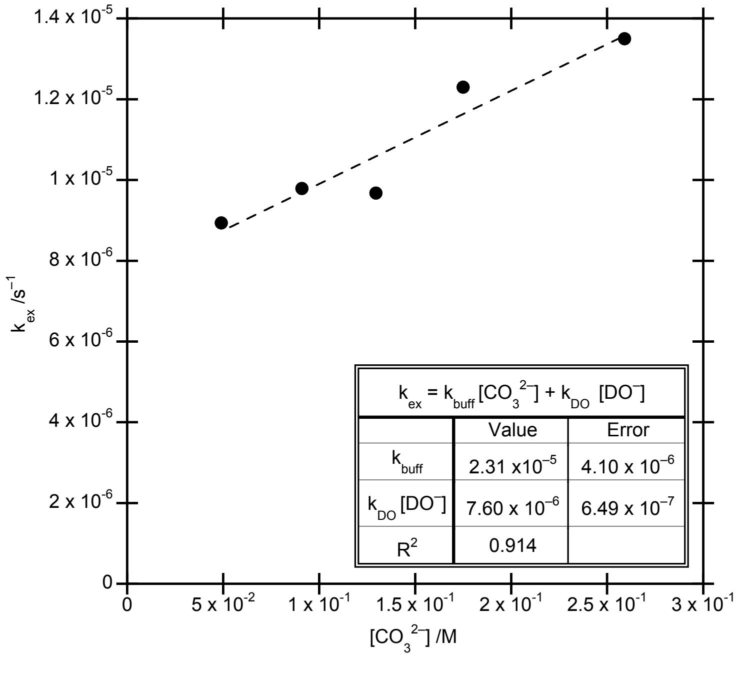

Figure S1.16: Plot of the dependence of k_{ex} upon $[DO^-]$ for the deuterium exchange of the prolyl α-proton H_D in c(D-Ala-L-Pro) (5.0 mM) in $KDCO_3$ (70 % free base, 0.07 – 0.37 M, pD = 10.85 – 10.98) buffered D_2O solution, I = 1.0 (KCl) and 25 ºC.

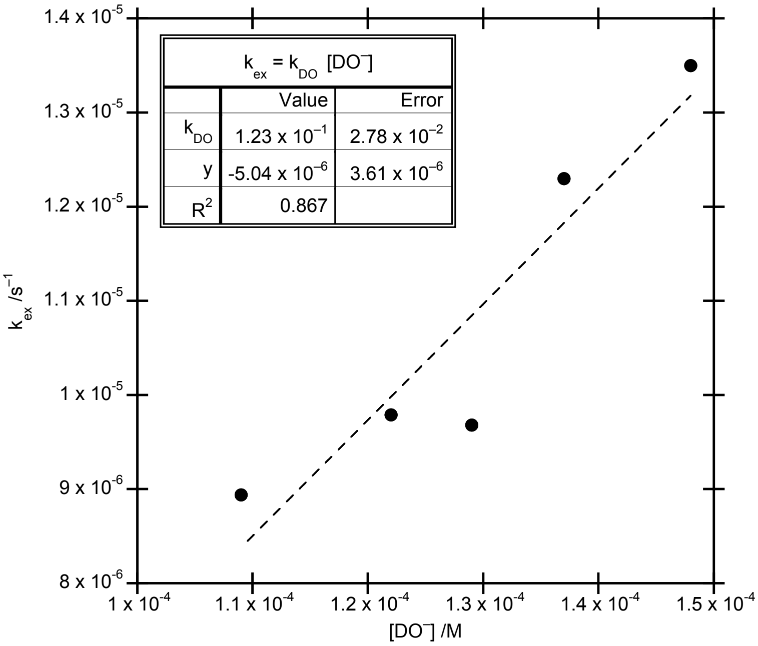

Figure S1.17: Plot of the dependence of k_{ex} upon $[CO_3^{2-}]$ for the deuterium exchange of the prolyl α-proton H_D in c(D-Ala-L-Pro) (5.0 mM) in $KDCO_3$ (20 % free base, 0.07 – 0.37 M, pD = 10.59 – 10.66) buffered D_2O solution, I = 1.0 (KCl) and 25 ºC.

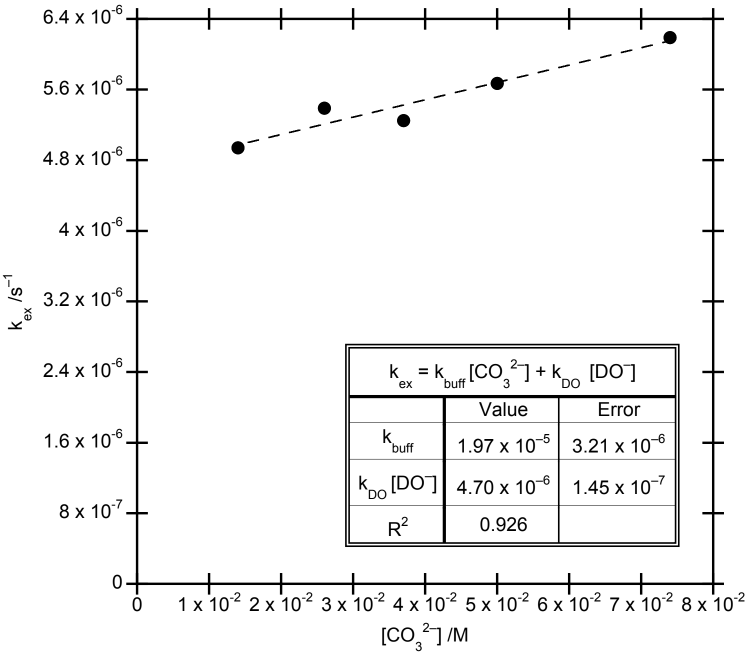

Figure S1.18: Plot of the dependence of k_{ex} upon $[DO^-]$ for the deuterium exchange of the prolyl α-proton H_D in c(D-Ala-L-Pro) (5.0 mM) in $KDCO_3$ (20 % free base, 0.07 – 0.37 M, pD = 10.59 – 10.66) buffered D_2O solution, I = 1.0 (KCl) and 25 ºC.

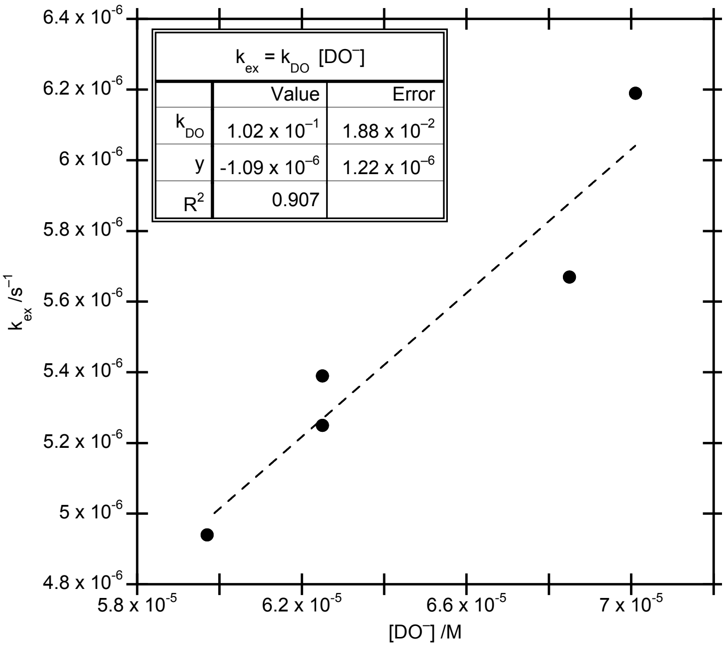

Figure S1.19: Plot of the dependence of k_{ex} upon $[CO_3^{2-}]$ for the deuterium exchange of the prolyl α-proton H_D in c(D-Ala-L-Pro) (5.0 mM) monitored via H_1 methyl protons on c(D-Ala-D-Pro) in $KDCO_3$ (70 % free base, 0.07 – 0.37 M, pD = 10.85 – 10.98) buffered D_2O solution, I = 1.0 (KCl) and 25 ºC.

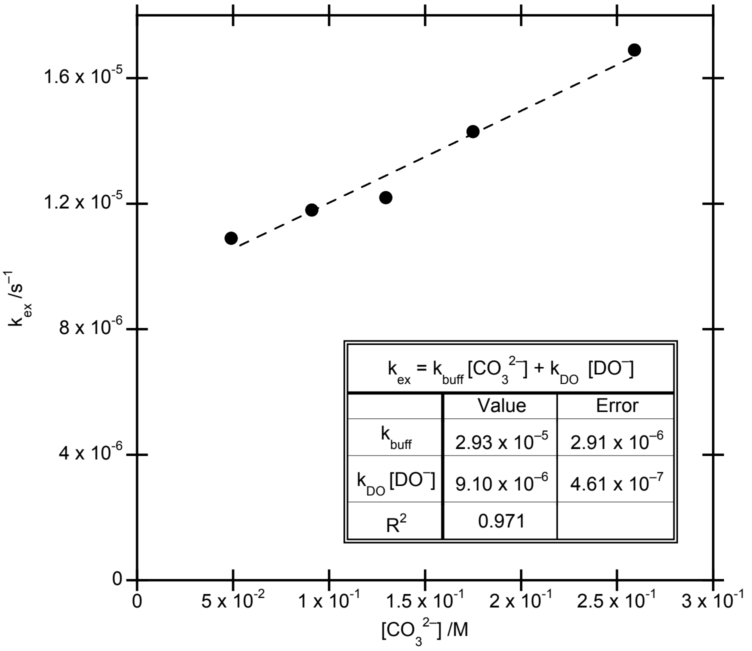

Figure S1.20: Plot of the dependence of k_{ex} upon $[DO^-]$ for the deuterium exchange of the prolyl α-proton H_D in c(D-Ala-L-Pro) (5.0 mM) monitored via H_1 methyl protons on c(D-Ala-D-Pro) in $KDCO_3$ (70 % free base, 0.07 – 0.37 M, pD = 10.85 – 10.98) buffered D_2O solution, I = 1.0 (KCl) and 25 ºC.

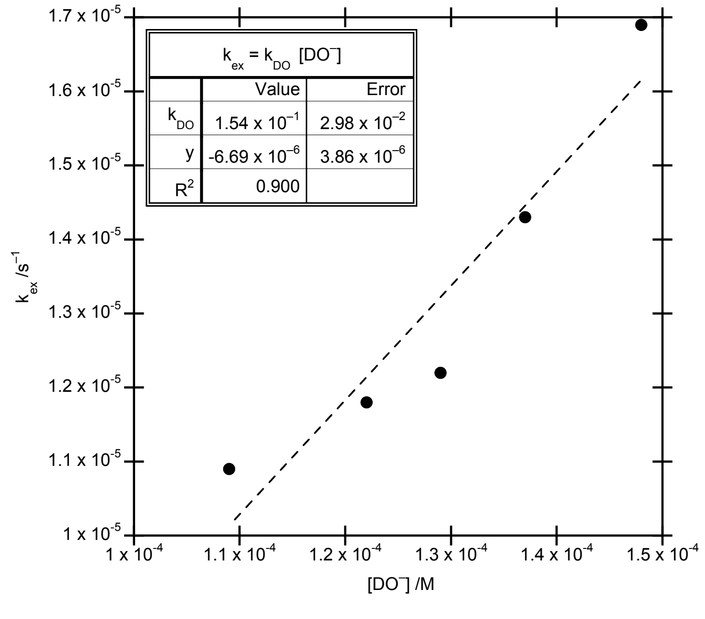

Figure S1.21: Plot of the dependence of k_{ex} upon $[CO_3^{2-}]$ for the deuterium exchange of the prolyl α-proton H_D in c(D-Ala-L-Pro) (5.0 mM) monitored via H_1 methyl protons on c(D-Ala-D-Pro) in $KDCO_3$ (20 % free base, 0.07 – 0.37 M, pD = 10.59 – 10.66) buffered D_2O solution, I = 1.0 (KCl) and 25 ºC.

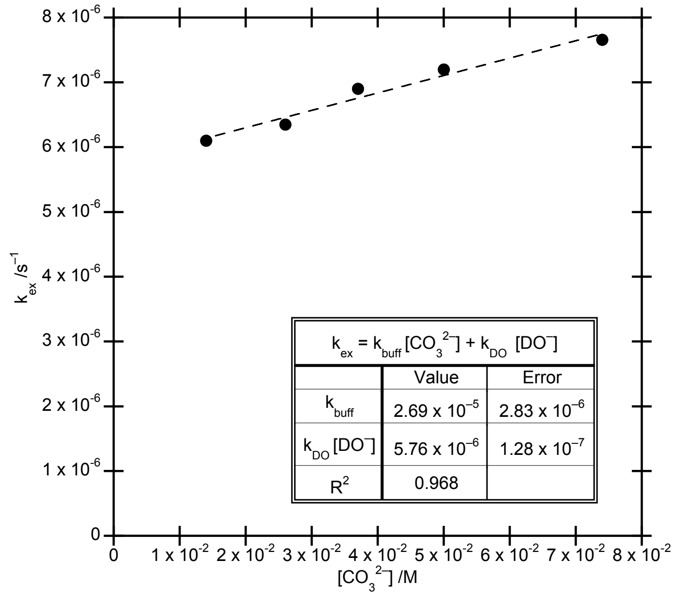

Figure S1.22: Plot of the dependence of k_{ex} upon $[DO^-]$ for the deuterium exchange of the prolyl α-proton H_D in c(D-Ala-L-Pro) (5.0 mM) monitored via H_1 methyl protons on c(D-Ala-D-Pro) in $KDCO_3$ (20 % free base, 0.07 – 0.37 M, pD = 10.59 – 10.66) buffered D_2O solution, I = 1.0 (KCl) and 25 ºC.

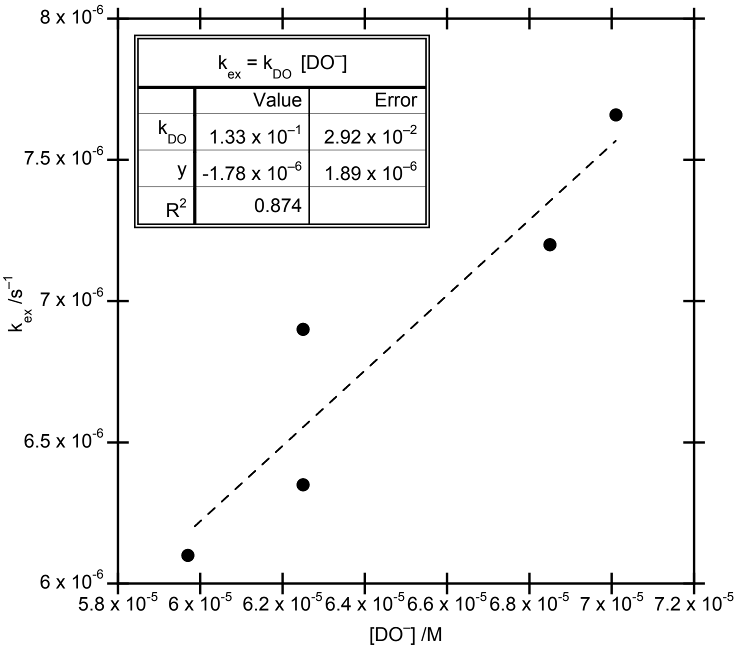

S1.4.7.2 c(D-Ala-L-Pro) Alanyl α-proton

The second order rate constant for the deuteroxide catalysed exchange, k_{DO}, for the alanyl α-proton of c(D-Ala-L-Pro) H_B may also be estimated from the deuterium exchange reaction data. This cannot be done by only monitoring the decrease in the integration from H_B as the interconversion to the c(D-Ala-D-Pro) diastereomer (via the faster deprotonation of the prolyl α-proton in c(D-Ala-L-Pro) and subsequent reprotonation upon the opposite face) also decreases the magnitude of the H_B peak. By summating the integral areas for the multiplets of both H_B (3.96 ppm, Figure S1.12) on c(D-Ala-L-Pro) and H_2 (4.21 ppm, Figure S1.12) on c(D-Ala-D-Pro) and assuming that $k_{DO}(H_B) \sim k_{DO}(H_2)$ an estimate for k_{DO} may be found for the alanyl α-proton in c(D-Ala-L-Pro).

The reaction data and k_{ex} values are shown in Table S1.12. Figure S1.23 shows the dependence of k_{ex} upon the concentration of deuteroxide and the fit of these data gives $k_{DO} = 5.51 \times 10^{-3}$ M^{-1} s^{-1}.

Table S1.12: Reaction data and observed first order rate constants (k_{ex}) for the deuterium exchange of the alanyl α-protons H_B in c(D-Ala-L-Pro) (5.0 mM) in $KDCO_3$ (0.185 M, pD = 10.12 – 10.98) buffered D_2O solution, I = 1.0 (KCl) and 25 ºC.

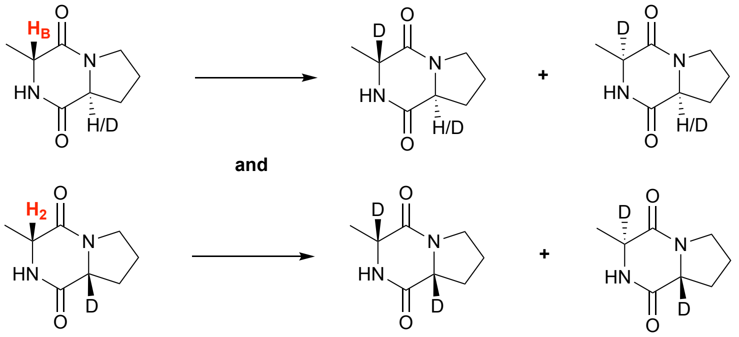

Experiment	Proton Chemical Shift /ppm	$[DO^-]$ /M	Time /s	$f(s)$[a]	k_{ex} /s^{-1}
70% fb BC	3.92–4.00	1.48×10^{-4}	1.06×10^{3}	1.00	7.81×10^{-7}
	+ 4.18–4.24	(pD 10.98)	2.43×10^{4}	0.80	
	H_B+H_2		8.10×10^{4}	0.51	
			1.11×10^{5}	0.44	
			2.68×10^{5}	0.27	
			3.41×10^{5}	0.23	
			3.73×10^{5}	0.22	
			4.46×10^{5}	0.20	
			5.19×10^{5}	0.20	
			6.15×10^{5}	0.18	
			6.87×10^{5}	0.17	
			8.76×10^{5}	0.16	
70%	3.92–4.00	9.90×10^{-5}	5.40×10^{2}	1.00	4.94×10^{-7}
	+ 4.18–4.24	(pD 10.81)	1.31×10^{4}	0.86	
	H_B+H_2		2.38×10^{4}	0.75	
			8.09×10^{4}	0.43	
			9.32×10^{4}	0.38	
			1.07×10^{5}	0.33	
			1.71×10^{5}	0.13	
			1.94×10^{5}	0.16	
			3.63×10^{5}	0.08	
			4.44×10^{5}	0.05	
			5.14×10^{5}	0.06	
			6.02×10^{5}	0.00	
60%	3.92–4.00	9.79×10^{-5}	1.06×10^{3}	1.00	6.06×10^{-7}

	+ 4.18–4.24	(p*D* 10.80)	1.46×10^{4}	0.97	
	H_B+H_2		7.75×10^{4}	0.94	
			1.01×10^{5}	0.91	
			1.64×10^{5}	0.89	
			2.52×10^{5}	0.85	
			2.72×10^{5}	0.83	
			3.35×10^{5}	0.82	
			3.58×10^{5}	0.79	
			5.97×10^{5}	0.68	
			7.68×10^{5}	0.65	
20% BC	3.92–4.00	7.01×10^{-5}	1.06×10^{3}	1.00	4.34×10^{-7}
	+ 4.18–4.24	(p*D* 10.66)	7.13×10^{4}	0.95	
	H_B+H_2		1.57×10^{5}	0.93	
			2.41×10^{5}	0.88	
			3.28×10^{5}	0.86	
			6.75×10^{5}	0.73	
			7.62×10^{5}	0.74	
			8.48×10^{5}	0.70	
50%fb	3.92–4.00	5.90×10^{-5}	1.07×10^{3}	1.00	2.53×10^{-7}
	+ 4.18–4.24	(p*D* 10.58)	5.10×10^{4}	0.99	
	H_B+H_2		1.38×10^{5}	0.98	
			2.25×10^{5}	0.91	
			4.01×10^{5}	0.88	
			4.85×10^{5}	0.89	
			5.73×10^{5}	0.87	
			6.59×10^{5}	0.84	
			7.45×10^{5}	0.82	
			8.40×10^{5}	0.82	
			1.01×10^{6}	0.76	
			1.09×10^{6}	0.77	
			1.19×10^{6}	0.74	
35% 0.37M	3.92–4.00	5.14×10^{-5}	1.19×10^{3}	1.00	3.25×10^{-7}
	+ 4.18–4.24	(p*D* 10.52)	6.70×10^{4}	0.96	
	H_B+H_2		1.50×10^{5}	0.93	
			2.35×10^{5}	0.89	
			4.96×10^{5}	0.82	
			5.84×10^{5}	0.81	

			6.73×10^{5}	0.82	
			7.76×10^{5}	0.79	
			8.43×10^{5}	0.74	
			1.10×10^{6}	0.73	
20%	3.92–4.00	4.27×10^{-5}	1.06×10^{3}	1.00	2.22×10^{-7}
	+ 4.18–4.24	(p*D* 10.44)	7.49×10^{4}	0.98	
	H_B+H_2		3.29×10^{5}	0.97	
			4.04×10^{5}	0.87	
			4.89×10^{5}	0.81	
			5.81×10^{5}	0.87	
			6.70×10^{5}	0.88	
			9.44×10^{5}	0.82	
			1.02×10^{6}	0.80	
			1.11×10^{6}	0.77	
			1.19×10^{6}	0.81	
35%	3.92–4.00	3.24×10^{-5}	1.06×10^{3}	1.00	1.63×10^{-7}
	+ 4.18–4.24	(p*D* 10.32)	8.42×10^{4}	1.02	
	H_B+H_2		1.53×10^{5}	0.96	
			4.07×10^{5}	0.93	
			4.89×10^{5}	0.91	
			5.73×10^{5}	0.89	
			6.65×10^{5}	0.90	
			7.51×10^{5}	0.87	
			1.02×10^{6}	0.80	
			1.10×10^{6}	0.84	
			1.18×10^{6}	0.88	
			1.27×10^{6}	0.82	
22%fb	3.92–4.00	2.04×10^{-5}	1.19×10^{3}	1.00	1.03×10^{-7}
	+ 4.18–4.24	(p*D* 10.12)	1.60×10^{4}	1.00	
	H_B+H_2		1.01×10^{5}	1.00	
			1.57×10^{5}	1.03	
			2.44×10^{5}	0.99	
			3.30×10^{5}	0.96	
			5.91×10^{5}	0.95	
			6.77×10^{5}	0.93	
			8.50×10^{5}	0.92	
			9.35×10^{5}	0.88	

1.19×10^6	0.89
1.29×10^6	0.86
1.37×10^6	0.86
1.46×10^6	0.84
1.54×10^6	0.86
1.81×10^6	0.85
1.89×10^6	0.83
1.98×10^6	0.83

[a] fraction of unreacted substrate remaining at each time point.

Figure S1.23: Plot of the dependence of k_{ex} upon [DO⁻] for the deuterium exchange of the alanyl α-proton H_B in c(D-Ala-L-Pro) (5.0 mM) in $KDCO_3$ (0.185 M, pD = 10.12 – 10.98) buffered D_2O solution, I = 1.0 (KCl) and 25 ºC.

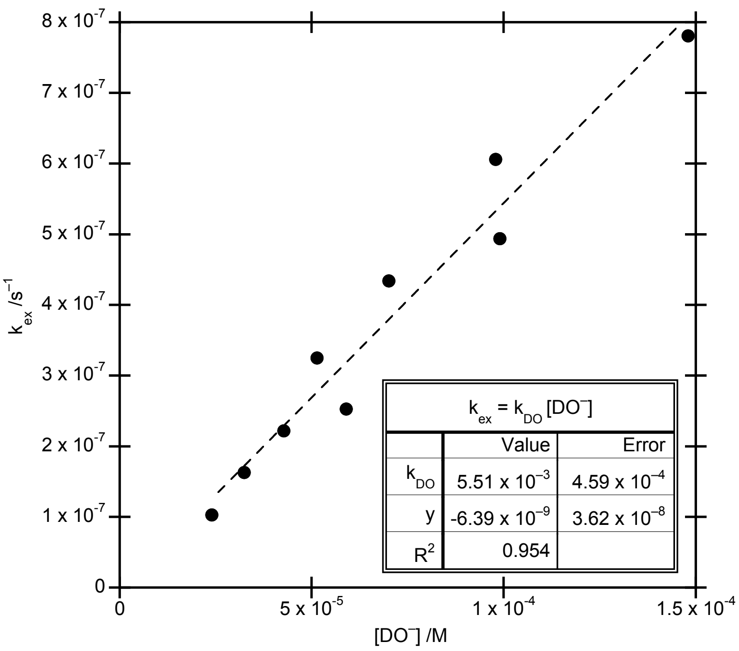

S1.4.8 Hydrogen-deuterium exchange reactions of c(L-Pro-L-Tyr)

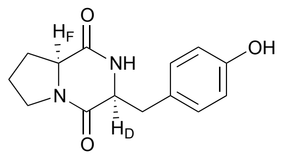

Pseudo first order rate constants for the deuteroxide catalysed exchange, k_{ex}, of the prolyl α-proton H_F and tyrosyl α-proton H_D of c(L-Pro-L-Tyr) were determined in $KDCO_3$ buffered D_2O solutions (pD = 10.30 – 10.94) using ^{1}H NMR spectroscopy (400 MHz).

S1.4.8.1 c(L-Pro-L-Tyr) Prolyl α-proton

A representative set of spectra taken at three time points during the reaction at p*D* = 10.57 is shown in Figure S1.24. Deuterium exchange at the H_D position on c(L-Pro-L-Tyr) resulted in disappearance of the doublet of doublets due to H_D at 4.30 ppm over the course of the reaction. The level of exchange was monitored relative to a tetramethylammonium deuteriosulfate internal standard, whose methyl protons (singlet, 3.14 ppm) are non-exchangeable. The products of deuterium exchange at H_D position on c(L-Pro-L-Tyr) are deuterated c(L-Pro-L-Tyr) and c(D-Pro-L-Tyr) (Scheme S5). Over the course of the reaction the equilibrium distribution of the diastereomers c(L-Pro-L-Tyr) and c(D-Pro-L-Tyr) is approached. Consequently, ^{1}H NMR spectra over the course of the reaction showed the disappearance of the doublet of doublets corresponding to the tyrosyl methylene group (H_C and $H_{C'}$) and the owing to the multiplet prolyl pyrrolidine ring protons (H_G, $H_{G'}$, H_I, $H_{I'}$ H_J and $H_{J'}$) on c(L-Pro-L-Tyr) and the appearance of the doublet of doublets for the analogous methylene (H_3 and $H_{3'}$) and the multiplets for the analogous pyrrolidine ring protons (H_7, $H_{7'}$, H_8, $H_{8'}$ H_9 and $H_{9'}$) on c(D-Pro-L-Tyr) (Figure S1.24).

Figure S1.24: Representative ^{1}H NMR spectra at 400 MHz for the deuterium exchange reaction of c(L-Pro-L-Tyr) (5.0 mM) in $KDCO_3$ (pD = 10.57, 50% free base, 0.185 M), 25 °C and ionic strength I = 1.0 M (KCl).

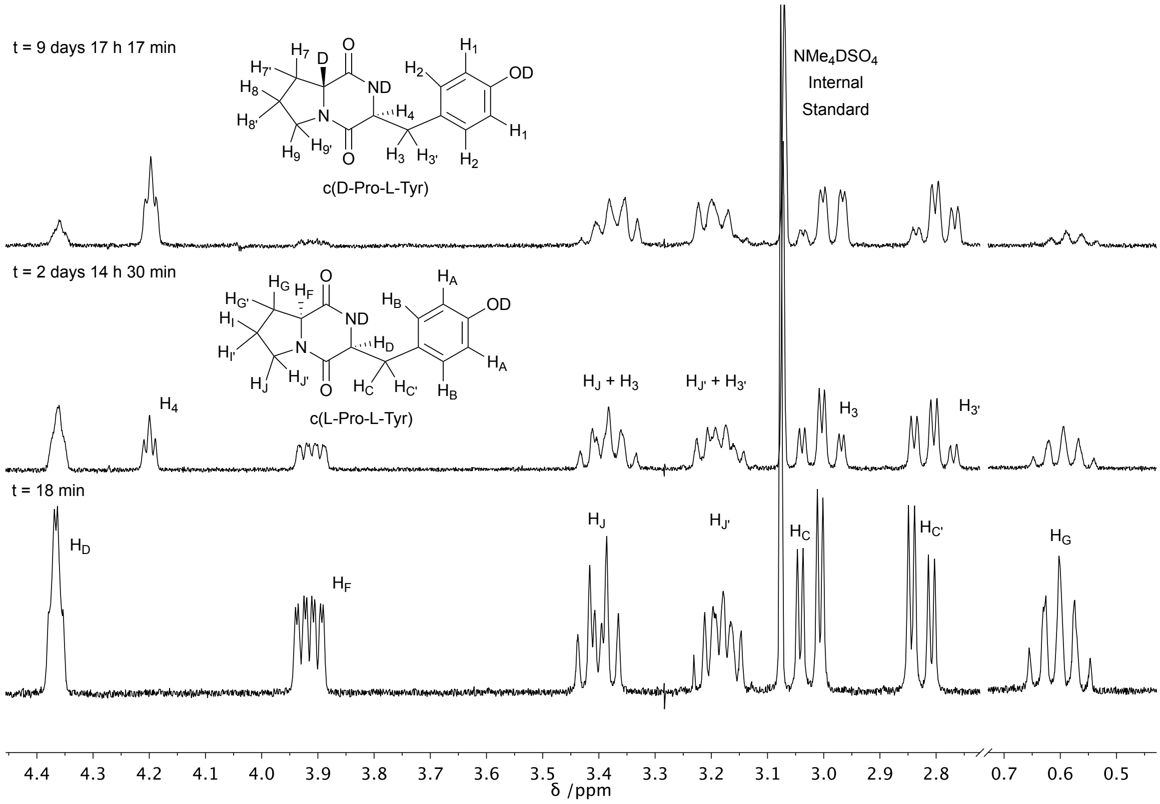

Scheme S5: Outcomes of the deuterium exchange reactions of c(L-Pro-L-Tyr).

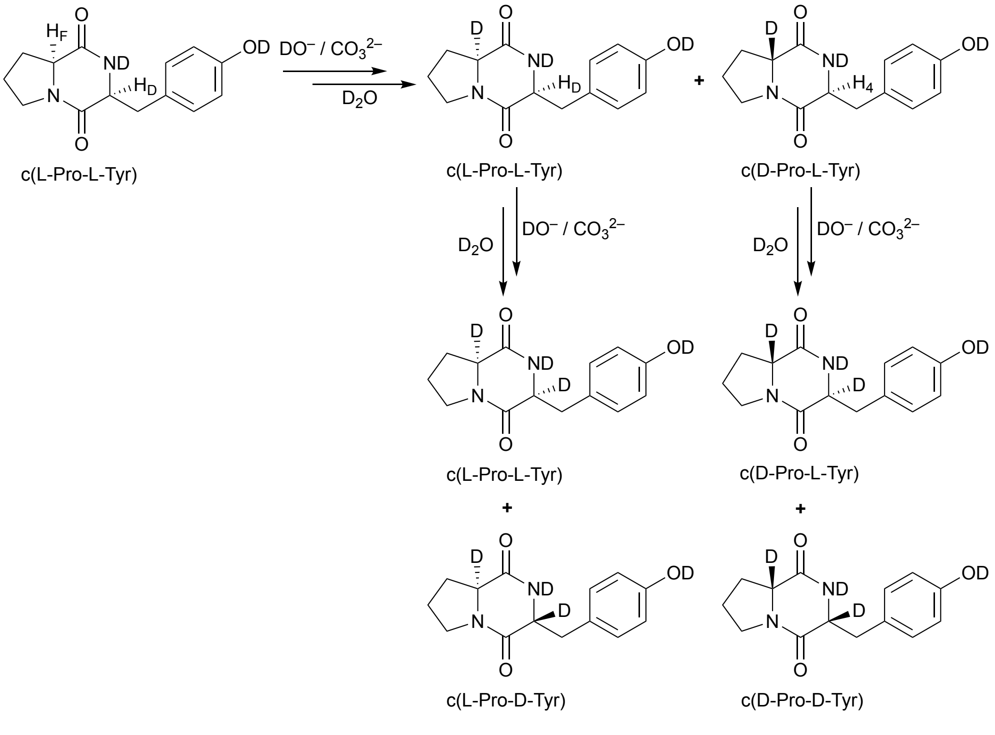

The phenol group on the parent amino acid tyrosine has a pK_a = 10.07 which is within 2 pK_a units of the pD range (pD = 10.30 – 10.94) used in the exchange experiments. Thus, both neutral and anionic c(L-Pro-L-Tyr) are present in solution. A negative charge on the DKP molecule would reduce the kinetic acidity (protofugality) of the α-protons towards proton transfer to deuteroxide, particularly for the tyrosyl α-proton. We were interested in obtaining the kinetic acidity of the α-protons solely from neutral c(L-Pro-L-Tyr). The concentration of neutral c(L-Pro-L-Tyr) decreases as the pD of the solution is increased and therefore first order observed rate constant for exchange, k_{ex}, will not be directly comparable. To correct for this phenolic acid dissociation, the k_{ex} values must be divided by the fraction of the neutral form of c(L-Pro-L-Tyr) present in solution, f_{OH}, to give the corrected first order observed rate constant for exchange, k_{ex}' (Eqn. S7).

$$k'_{ex} = \frac{k_{ex}}{f_{OH}} \qquad \text{(Eqn. S7)}$$

The fraction of neutral c(L-Pro-L-Tyr) present in solution can be found from the pK_a of the tyrosyl phenol group in c(L-Pro-L-Tyr). A UV-Visible spectrophotometric experiment was performed to determine the pK_a of the tyrosyl phenol group in c(L-Pro-L-Tyr). The changes in absorption from the anionic phenol group (λ_{max} = 293 nm) over the pD range pD = 4.89 – 14.01 were measured (Figure S1.25). In order to solubilise c(L-Pro-L-Tyr) a 9 : 1 D_2O : d_3-MeCN solution was required.

S1.4.8.2 UV-Vis Spectrophotometric determination of the tyrosyl phenolic pK_a on c(L-Pro-L-Tyr)

A 20 mM stock solution of c(L-Pro-L-Tyr) was prepared by dissolving 26.09 mg (106.8 mmol) of c(L-Pro-L-Tyr) in 5.00 mL of 9 : 1 D_2O : d_3-MeCN. Buffer solutions (KAcO/AcOH, $N(EtOD)_3/N(EtOD)_3D^+$, $KDCO_3/K_2CO_3$ and KOD) with a 9 : 1 D_2O : d_3-MeCN solvent composition were prepared over the pH range pH = 5.29 – 14.41. A solution of 2 M potassium chloride in 9 : 1 D_2O : d_3-MeCN was used to adjust the ionic strength to I = 1.0 M.

All absorbance spectra were obtained on a Cary 100 UV-Vis spectrophotometer. A 1.0 mL cuvette was filled with a buffer solution and placed in the temperature regulated cuvette holder of the UV-Vis spectrophotometer. The solution was allowed to equilibrate to 25 °C prior to recording the absorbance spectrum over the wavelength range 190 – 400 nm (the UV-Vis spectrophotometer had been zeroed against air). 41 μL of the 20 mM c(L-Pro-L-Tyr) stock

solution was added to the cuvette to give a 0.8 mM solution of c(L-Pro-L-Tyr) and the cuvette inverted several times to ensure good mixing. The absorbance spectrum over the wavelength range 190 – 400 nm was once again taken and the previous absorbance spectrum of the solution was manually subtracted from this to leave just the absorbance from c(L-Pro-L-Tyr). This procedure was repeated for every buffer prepared in the pH range pH = 5.29 – 14.41. The absorbance from the phenolate group was located at λ = 293 nm. The plot of the changes in absorbance at 293 nm with pH is shown in Figure S1.25.

Figure S1.25: Plot of the dependence of absorption from phenol group in c(L-Pro-L-Tyr) (0.8 mM)[v] upon the p*D* of solution (p*D* = 4.89 – 14.01) with λ_{max} = 293 nm in a 9 : 1 D_2O : d_3-MeCN solution, *I* = 1.0 M, at 25 °C.

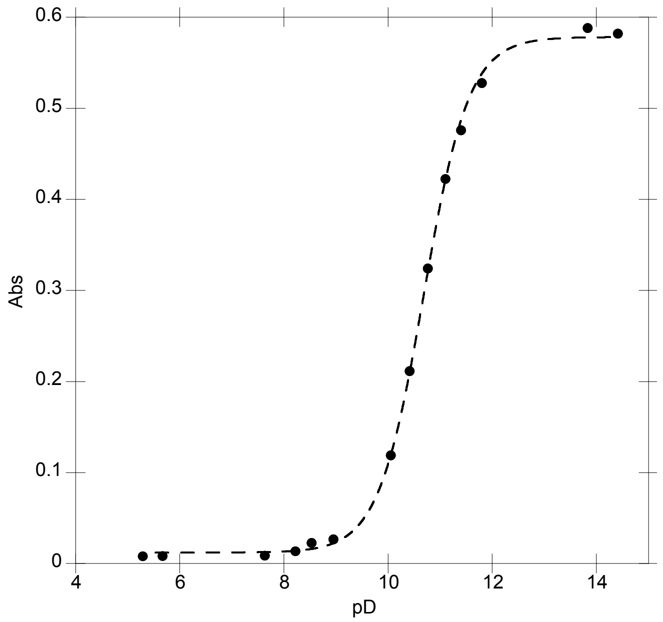

$A_{obs} = (K_a*A_{max} + A_{min}*10^{-pD}) / (10^{-pD} + K_a)$		
	Value	Error
K_a	2.07×10^{-11}	7.29×10^{-13}
A_{max}	5.78×10^{-1}	3.88×10^{-3}
A_{min}	1.23×10^{-2}	2.70×10^{-3}
R^2	0.999	

The data in Figure S1.25 was fitted to Eqn. S8 to determine the K_a, where A_{max} is the absorbance when the tyrosyl phenol in c(L-Pro-L-Tyr) is fully deprotonated and A_{min} is the

[v] This c(L-Pro-L-Tyr) concentration of 0.8 mM is lower than that used in the deuterium exchange experiments. The d_3-MeCN co-solvent was required in order to prepare a 20.0 mM c(L-Pro-L-Tyr) stock solution. It was also necessary to prepare the buffer solutions with 9 : 1 D_2O : d_3-MeCN in order to able to correct for the contribution of d_3-MeCN to the observed absorbance.

absorbance when fully protonated. The fit gives a $K_a = 2.07 \times 10^{-11}$ corresponding to pK_a = 10.68 for the tyrosyl phenol group in c(L-Pro-L-Tyr).

$$A_{obs} = \frac{K_a\,A_{max} + A_{min}\,10^{-pD}}{10^{-pD} + K_a} \qquad \text{(Eqn. S8)}$$

This pK_a is for 9 : 1 D_2O : d_3-MeCN solution but the pK_a in a 100 % D_2O is expected to be very similar. Acid dissociation constants are typically not substantially affected by the addition of a co-solvent until the composition of the co-solvent increases above 40 % v/v. In all subsequent Figures in this section the corrected first order observed rate constant for exchange, k_{ex}', are plotted rather than k_{ex}.

In order to determine the second order rate constant for deuteroxide catalysed exchange, k_{DO}, for the prolyl α-proton of c(L-Pro-L-Tyr) H_F the hydrogen deuterium exchange reactions were performed in $KDCO_3$ buffers over the pD range 10.30 – 10.94. The corrected first order rate constant for exchange, k_{ex}', was determined by monitoring exchange of the prolyl proton H_F on c(L-Pro-L-Tyr). Figure S1.26 shows the dependence of k_{ex}' on the concentration of deuteroxide for H_F (Table S1.13). The fit of the data gives a value of $k_{DO} = 1.19 \times 10^{-1}$ M^{-1} s^{-1} for H_F.

Table S1.13: Reaction data and observed first order rate constants (k_{ex}) for the deuterium exchange of the prolyl α-proton H_F in c(L-Pro-L-Tyr) (5.0 mM) in $KDCO_3$ (0.185 M, pD = 10.30 – 10.81) buffered D_2O solution, I = 1.0 (KCl) and 25 ºC.

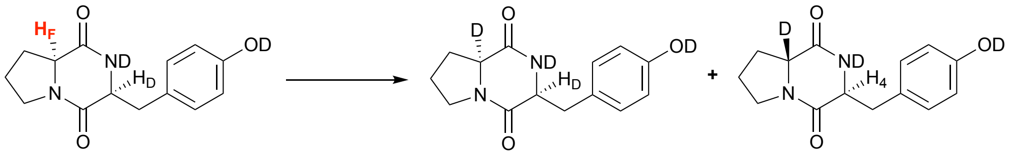

Experiment	Proton Chemical Shift /ppm	$[DO^-]$ /M	Time /s	$f(s)^a$	k_{ex} /s^{-1}	k_{ex}/f_{OH} /s^{-1}
70% fb	3.88 – 3.96	9.90×10^{-5}	5.40×10^{2}	1.00	4.47×10^{-6}	1.04×10^{-5}
	H_F	(pD 10.81)	1.23×10^{4}	0.96		
			2.30×10^{4}	0.92		
			7.94×10^{4}	0.70		
			9.18×10^{4}	0.66		
			1.06×10^{5}	0.61		
			1.71×10^{5}	0.47		

			1.92×10^{5}	0.43		
			3.61×10^{5}	0.20		
			4.42×10^{5}	0.13		
			5.13×10^{5}	0.10		
			5.97×10^{5}	0.07		
			6.85×10^{5}	0.05		
			7.71×10^{5}	0.04		
			1.03×10^{6}	0.00		
50% fb	3.88 – 3.96	5.76×10^{-5}	1.07×10^{3}	1.00	2.85×10^{-6}	5.05×10^{-6}
	H_F	(p*D* 10.57)	5.09×10^{4}	0.86		
			1.38×10^{5}	0.65		
			2.25×10^{5}	0.53		
			4.01×10^{5}	0.31		
			4.86×10^{5}	0.25		
			5.73×10^{5}	0.19		
			6.58×10^{5}	0.16		
			7.44×10^{5}	0.13		
			8.40×10^{5}	0.09		
			1.01×10^{6}	0.06		
			1.09×10^{6}	0.04		
			1.19×10^{6}	0.04		
20% fb	3.88 – 3.96	3.99×10^{-5}	1.06×10^{3}	1.00	2.05×10^{-6}	3.13×10^{-6}
	H_F	(p*D* 10.41)	7.60×10^{4}	0.86		
			3.40×10^{5}	0.50		
			4.12×10^{5}	0.43		
			4.96×10^{5}	0.36		
			5.88×10^{5}	0.32		
			6.79×10^{5}	0.24		
			9.52×10^{5}	0.15		
			1.02×10^{6}	0.12		
			1.11×10^{6}	0.11		
			1.19×10^{6}	0.08		
35% fb	3.88 – 3.96	3.10×10^{-5}	1.06×10^{3}	1.00	1.75×10^{-6}	2.48×10^{-6}
	H_F	(p*D* 10.30)	8.40×10^{4}	0.87		
			1.57×10^{5}	0.75		
			4.10×10^{5}	0.48		
			4.88×10^{5}	0.42		

5.73×10^5	0.37
6.65×10^5	0.30
7.51×10^5	0.28
1.02×10^6	0.17
1.10×10^6	0.15
1.19×10^6	0.13
1.27×10^6	0.12

[a] fraction of unreacted substrate remaining at each time point.

Figure S1.26: Plot of the dependence of k_{ex}' upon [DO⁻] for the deuterium exchange of the prolyl α-proton H_F in c(L-Pro-L-Tyr) (5.0 mM) in $KDCO_3$ (0.185 M, pD = 10.30 – 10.81) buffered D_2O solution, I = 1.0 (KCl) and 25 ºC.

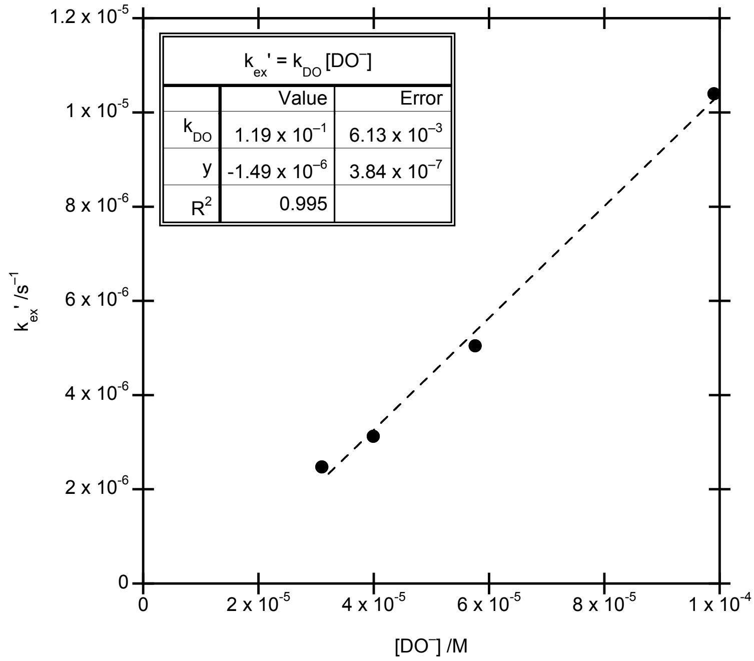

As for other DKP substrates, H / D exchange reactions were also undertaken in a series of solutions with an identical buffer ratio and a varying total concentration of buffer, [$KDCO_3$]. The H / D exchange reactions were performed at both 70 % free base and 20 % free base $KDCO_3$ over the [$KDCO_3$] range 0.12 – 0.37 M (experimentally, the pD varied by 0.09 – 0.11). Figures 2.27 and 2.28 show the dependence of k_{ex}' on the [CO_3^{2-}] (Table S1.14 – S1.15) while Figure S1.29 and Figure S1.30 show the dependence of k_{ex}' on the [DO⁻]. In the former Figures the fit of the data would give $k_{buff} = 1.79 \times 10^{-5} - 4.51 \times 10^{-5}$ M^{-1} s^{-1}. However, the fit of the data in the latter Figures gives $k_{DO} = 1.65 \times 10^{-1} - 2.20 \times 10^{-1}$ M^{-1} s^{-1} which are in reasonable

agreement with the $k_{DO} = 1.19 \times 10^{-1}$ M^{-1} s^{-1} determined in Figure S1.26 above. Thus, as for the other DKP substrates, the changes in k_{ex} observed in the above experiments are most likely due changes in [DO$^-$] rather than buffer concentration.

Table S1.14: Reaction data and observed first order rate constants (k_{ex}) for the deuterium exchange of the prolyl α-proton H_F in c(L-Pro-L-Tyr) (5.0 mM) in 70 % free base $KDCO_3$ (0.13 – 0.37 M, pD = 10.83 – 10.94) buffered D_2O solution, I = 1.0 (KCl) and 25 °C.

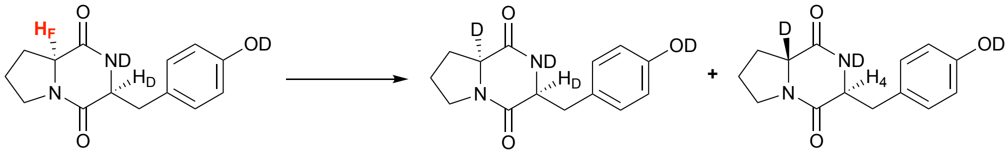

$[KDCO_3]$ /M	$[CO_3^{2-}]$ /M	[DO$^-$] /M	Time /s	f(s)[a]	k_{ex} /s^{-1}	k_{ex}/f_{OH} /s^{-1}
0.37	0.26	1.35×10^{-4}	1.06×10^3	1.00	6.74×10^{-6}	1.89×10^{-5}
		(pD 10.94)	5.90×10^4	0.68		
			9.03×10^4	0.55		
			1.48×10^5	0.37		
			2.38×10^5	0.20		
			2.64×10^5	0.16		
			3.38×10^5	0.10		
			4.97×10^5	0.03		
			5.23×10^5	0.02		
			5.82×10^5	0.02		
			6.80×10^5	0.01		
0.25	0.18	1.28×10^{-4}	1.26×10^3	1.00	5.83×10^{-6}	1.58×10^{-5}
		(pD 10.92)	5.91×10^4	0.71		
			8.63×10^4	0.60		
			1.48×10^5	0.42		
			1.75×10^5	0.36		
			2.49×10^5	0.24		
			4.05×10^5	0.09		
			4.32×10^5	0.08		
			4.93×10^5	0.06		
			5.85×10^5	0.04		
			6.66×10^5	0.02		
0.185	0.13	1.10×10^{-4}	1.06×10^3	1.00	5.23×10^{-6}	1.29×10^{-5}
		(pD 10.85)	5.90×10^4	0.75		

			9.02×10^{4}	0.63		
			1.48×10^{5}	0.45		
			2.40×10^{5}	0.28		
			2.64×10^{5}	0.25		
			3.38×10^{5}	0.17		
			5.00×10^{5}	0.07		
			5.23×10^{5}	0.06		
			5.82×10^{5}	0.04		
			6.72×10^{5}	0.03		
			7.53×10^{5}	0.02		
0.13	0.09	1.04×10^{-4}	1.06×10^{3}	1.00	4.81×10^{-6}	1.15×10^{-5}
		(pD 10.83)	5.88×10^{4}	0.76		
			9.02×10^{4}	0.64		
			1.48×10^{5}	0.49		
			2.47×10^{5}	0.30		
			2.64×10^{5}	0.29		
			3.38×10^{5}	0.20		
			5.01×10^{5}	0.09		
			5.23×10^{5}	0.08		
			5.82×10^{5}	0.05		
			6.73×10^{5}	0.05		
			7.53×10^{5}	0.03		

[a] fraction of unreacted substrate remaining at each time point.

Table S1.15: Reaction data and observed first order rate constants (k_{ex}) for the deuterium exchange of the prolyl α-proton H_F in c(L-Pro-L-Tyr) (5.0 mM) in 20 % free base $KDCO_3$ (0.13 – 0.37 M, pD = 10.19 – 10.28) buffered D_2O solution, I = 1.0 (KCl) and 25 ºC.

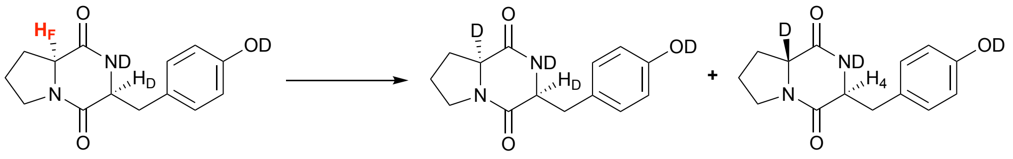

[$KDCO_3$] /M	[CO_3^{2-}] /M	[DO^-] /M	Time /s	f(s)[a]	k_{ex} /s^{-1}	k_{ex}/f_{OH} /s^{-1}
0.370	0.074	7.42×10^{-5}	1.06×10^{3}	1.00	3.59×10^{-6}	5.00×10^{-6}
		(pD 10.28)	7.47×10^{4}	0.76		
			1.61×10^{5}	0.56		
			2.47×10^{5}	0.41		
			5.22×10^{5}	0.15		

			5.95×10^{5}	0.12		
			6.87×10^{5}	0.09		
			7.67×10^{5}	0.06		
			8.53×10^{5}	0.05		
0.250	0.050	6.85×10^{-5}	1.06×10^{3}	1.00	3.18×10^{-6}	4.33×10^{-6}
		(p*D* 10.25)	7.65×10^{4}	0.78		
			1.74×10^{5}	0.58		
			2.47×10^{5}	0.47		
			5.21×10^{5}	0.18		
			5.94×10^{5}	0.15		
			6.87×10^{5}	0.12		
			7.66×10^{5}	0.08		
			8.52×10^{5}	0.06		
0.185	0.037	6.62×10^{-5}	1.06×10^{3}	1.00	2.99×10^{-6}	4.05×10^{-6}
		(p*D* 10.23)	8.24×10^{4}	0.79		
			1.72×10^{5}	0.61		
			2.44×10^{5}	0.47		
			5.18×10^{5}	0.21		
			5.91×10^{5}	0.17		
			6.85×10^{5}	0.13		
			7.63×10^{5}	0.10		
			8.49×10^{5}	0.08		
0.120	0.024	6.04×10^{-5}	1.06×10^{3}	1.00	2.67×10^{-6}	3.52×10^{-6}
		(p*D* 10.19)	8.02×10^{4}	0.82		
			1.71×10^{5}	0.63		
			2.56×10^{5}	0.50		
			5.17×10^{5}	0.25		
			5.89×10^{5}	0.21		
			6.83×10^{5}	0.17		
			7.63×10^{5}	0.13		
			8.47×10^{5}	0.10		

[a] fraction of unreacted substrate remaining at each time point.

Figure S1.27: Plot of the dependence of k_{ex} upon $[CO_3^{2-}]$ for the deuterium exchange of the prolyl α-proton H_F in c(L-Pro-L-Tyr) (5.0 mM) in $KDCO_3$ (70 % free base, 0.13 – 0.37 M, p*D* = 10.83 – 10.94) buffered D_2O solution, *I* = 1.0 (KCl) and 25 ºC.

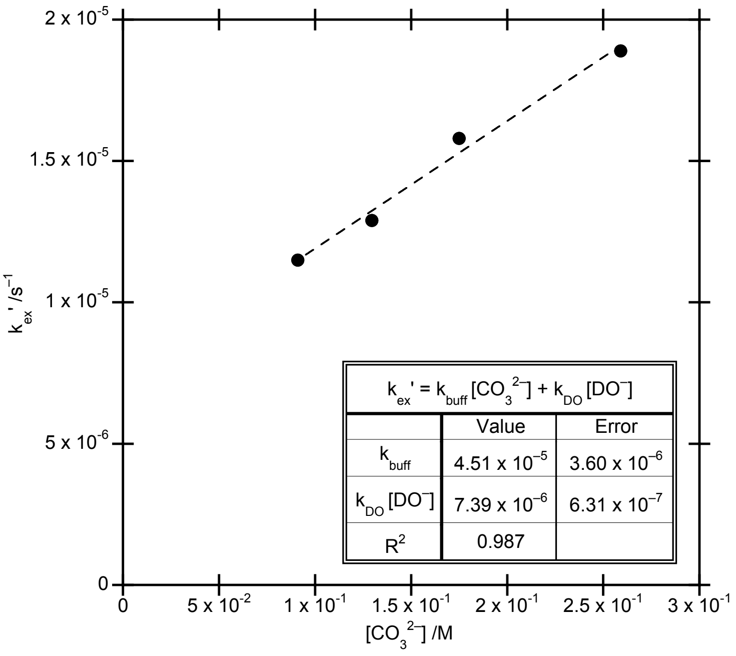
2 x 10^-5
1.5 x 10^-5
1 x 10^-5
5 x 10^-6
0
k_ex ' /s^-1
0
5 x 10^-2
1 x 10^-1
1.5 x 10^-1
2 x 10^-1
2.5 x 10^-1
3 x 10^-1
[CO_3^2−] /M
k_ex ' = k_buff [CO_3^2−] + k_DO [DO^−]
Value
Error
k_buff
4.51 x 10^−5
3.60 x 10^−6
k_DO [DO^−]
7.39 x 10^−6
6.31 x 10^−7
R^2
0.987

Figure S1.28: Plot of the dependence of k_{ex} upon $[CO_3^{2-}]$ for the deuterium exchange of the prolyl α-proton H_F in c(L-Pro-L-Tyr) (5.0 mM) in $KDCO_3$ (20 % free base, 0.12 – 0.37 M, pD = 10.19 – 10.28) buffered D_2O solution, I = 1.0 (KCl) and 25 ºC.

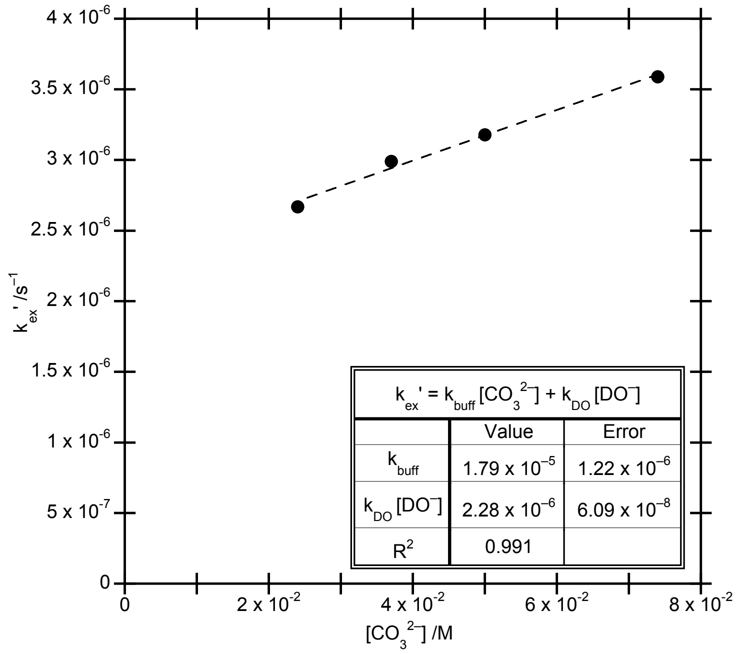

Figure S1.29: Plot of the dependence of k_{ex} upon $[DO^-]$ for the deuterium exchange of the prolyl α-proton H_F in c(L-Pro-L-Tyr) (5.0 mM) in $KDCO_3$ (70% free base, 0.13 – 0.37 M, pD = 10.83 – 10.94) buffered D_2O solution, I = 1.0 (KCl) and 25 ºC.

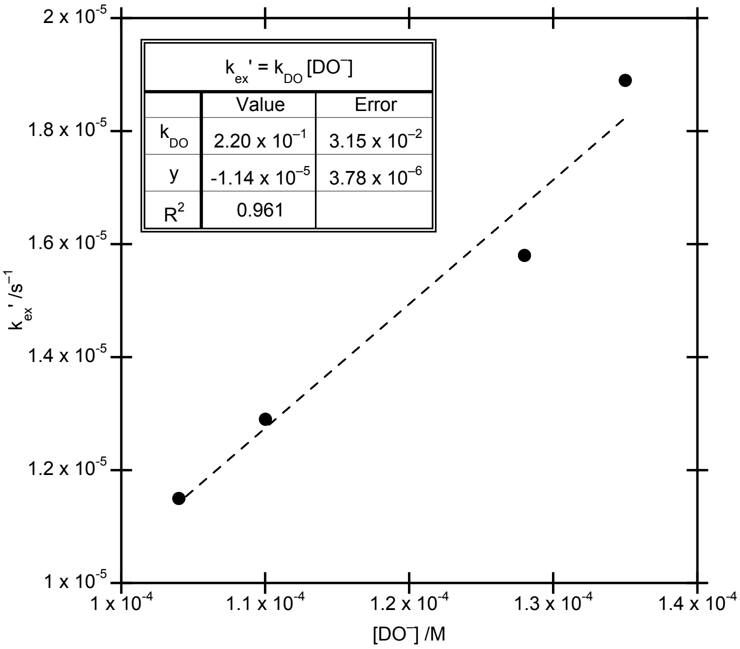

Figure S1.30: Plot of the dependence of k_{ex} upon [DO⁻] for the deuterium exchange of the prolyl α-proton H_F in c(L-Pro-L-Tyr) (5.0 mM) in $KDCO_3$ (20% free base, 0.12 – 0.37 M, pD = 10.19 – 10.28) buffered D_2O solution, I = 1.0 (KCl) and 25 ºC.

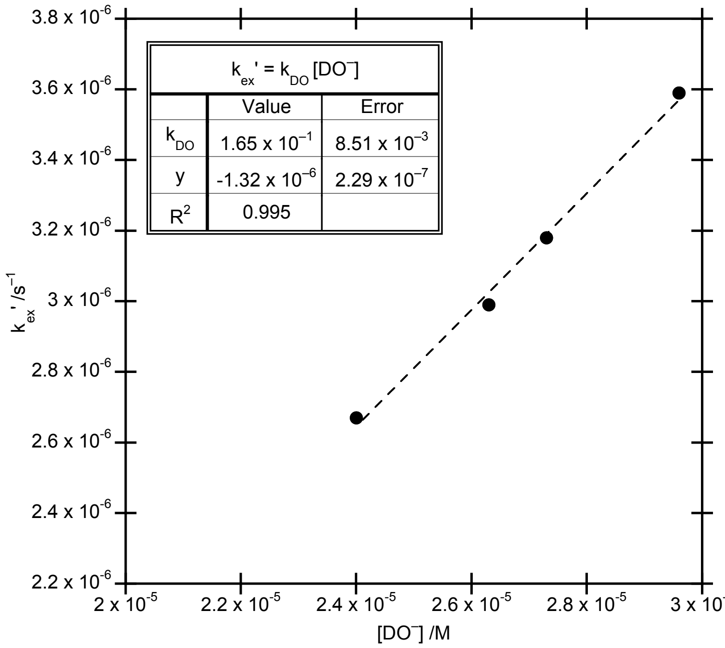

S1.4.8.3 c(L-Pro-L-Tyr) Tyrosyl α-proton

In order to determine the second order rate constant for deuteroxide catalysed exchange, k_{DO}, for the tyrosyl α-proton of c(L-Pro-L-Tyr), H_D, the hydrogen deuterium exchange reactions were performed in $KDCO_3$ buffers over the pD range 10.23 – 10.85. The corrected pseudo-first order rate constant for exchange, k_{ex}', was determined by monitoring exchange of the summated integral area from prolyl proton H_D on c(L-Pro-L-Tyr) (δ 4.37 ppm) and H_4 on c(D-Pro-L-Tyr) (δ 4.19 ppm) (Table S1.16). This approach assumes that k_{ex}' values for H_D on c(L-Pro-L-Tyr) and H_4 on c(D-Pro-L-Tyr) are equivalent. Figure S1.31 shows the dependence of k_{ex}' on the concentration of deuteroxide for H_D and the fit of the reaction data to Eqn. S5 gives $k_{DO} = 2.09 \times 10^{-3}$ M^{-1} s^{-1}.

Table S1.16: Reaction data and observed first order rate constants (k_{ex}) for the deuterium exchange of the tyrosyl α-protons H_D in c(L-Pro-L-Tyr) and H_4 in c(D-Pro-L-Tyr) (5.0 mM) in $KDCO_3$ (0.185 M, pD = 10.23 – 10.85) buffered D_2O solution, I = 1.0 (KCl) and 25 °C.

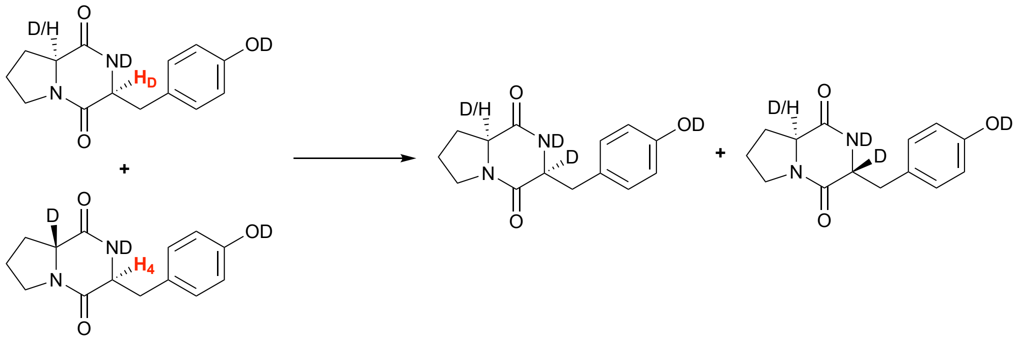

Experiment	[DO⁻] /M	Time /s	f(s)[a]	k_{ex} /s^{-1}	k_{ex}/f_{OH} /s^{-1}
70%	9.90×10^{-5}	5.40×10^{2}	1.00	1.08×10^{-7}	4.66×10^{-7}
	(pD 10.81)	1.23×10^{4}	1.01		
		2.30×10^{4}	1.02		
		7.94×10^{4}	0.99		
		9.18×10^{4}	0.99		
		1.06×10^{5}	1.00		
		1.71×10^{5}	0.99		
		1.92×10^{5}	0.98		
		3.61×10^{5}	0.98		
		4.42×10^{5}	0.94		
		5.13×10^{5}	0.94		
		5.97×10^{5}	0.94		
		6.85×10^{5}	0.93		
		7.71×10^{5}	0.93		
		1.03×10^{5}	0.88		
70% BC	1.10×10^{-4}	1.06×10^{3}	1.00	1.28×10^{-7}	5.98×10^{-7}
	(pD 10.85)	5.90×10^{4}	1.01		
		9.02×10^{4}	0.96		
		1.48×10^{5}	0.96		
		2.40×10^{5}	0.97		
		2.64×10^{5}	0.97		
		3.38×10^{5}	0.94		
		5.00×10^{5}	0.93		
		5.23×10^{5}	0.93		
		5.82×10^{5}	0.93		

		6.72×10^{5}	0.92		
		7.53×10^{5}	0.89		
		1.06×10^{6}	0.87		
		1.14×10^{6}	0.86		
		1.23×10^{6}	0.88		
50%	5.76×10^{-5}	1.07×10^{3}	1.00	1.17×10^{-7}	3.43×10^{-7}
	(p*D* 10.57)	5.09×10^{4}	0.98		
		1.38×10^{5}	0.97		
		2.25×10^{5}	0.96		
		4.01×10^{5}	0.95		
		4.86×10^{5}	0.92		
		5.73×10^{5}	0.96		
		6.58×10^{5}	0.96		
		7.44×10^{5}	0.90		
		8.40×10^{5}	0.89		
		1.01×10^{6}	0.90		
		1.09×10^{6}	0.88		
		1.19×10^{6}	0.87		
20%	3.99×10^{-5}	1.06×10^{3}	1.00	8.01×10^{-8}	1.87×10^{-7}
	(p*D* 10.41)	7.60×10^{4}	1.01		
		3.40×10^{5}	1.00		
		4.12×10^{5}	0.96		
		4.96×10^{5}	0.95		
		5.88×10^{5}	1.01		
		6.79×10^{5}	0.94		
		9.52×10^{5}	0.93		
		1.02×10^{6}	0.92		
		1.11×10^{6}	1.00		
		1.19×10^{6}	0.91		
35%	3.10×10^{-5}	1.06×10^{3}	1.00	8.44×10^{-8}	1.72×10^{-7}
	(p*D* 10.30)	8.40×10^{4}	0.97		
		1.57×10^{5}	0.96		
		4.10×10^{5}	0.96		
		4.88×10^{5}	0.94		
		5.73×10^{5}	0.96		
		6.65×10^{5}	0.94		
		7.51×10^{5}	0.94		

		1.02×10^{6}	0.94		
		1.10×10^{6}	0.91		
		1.19×10^{6}	0.90		
		1.27×10^{6}	0.91		
20% BC	2.63×10^{-5}	1.06×10^{3}	1.00	1.09×10^{-7}	2.06×10^{-7}
	(p*D* 10.23)	8.24×10^{4}	0.98		
		1.72×10^{5}	0.96		
		2.44×10^{5}	0.97		
		5.18×10^{5}	0.94		
		5.91×10^{5}	0.96		
		6.85×10^{5}	0.93		
		7.63×10^{5}	0.92		
		8.49×10^{5}	0.90		

[a] fraction of unreacted substrate remaining at each time point.

Figure S1.31: Plot of the dependence of k_{ex}' upon [DO⁻] for the deuterium exchange of the prolyl α-protons H_D in c(L-Pro-L-Tyr) and H_4 in c(D-Pro-L-Tyr) (5.0 mM) in $KDCO_3$ (0.185 M, pD = 10.23 – 10.85) buffered D_2O solution, at I = 1.0 (KCl) and 25 ºC.

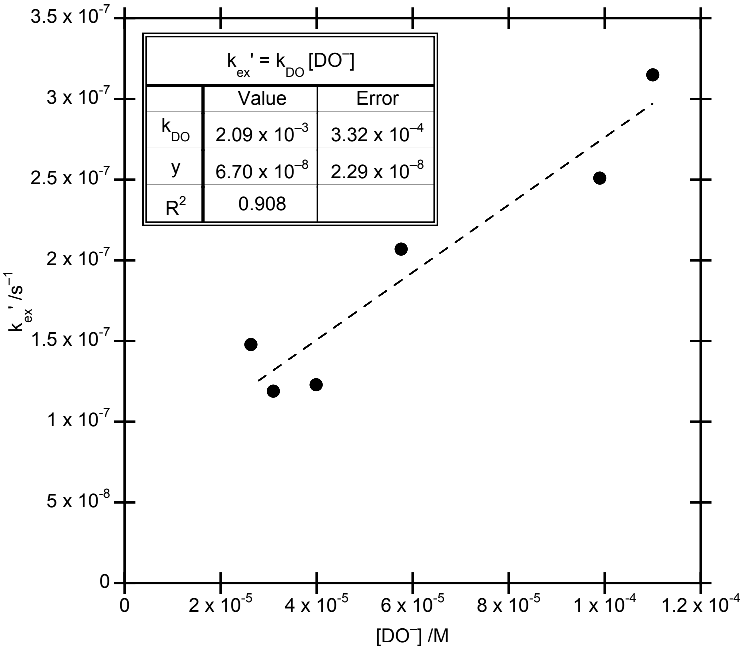

S1.4.9 Hydrogen-deuterium exchange reactions of c(Gly-L-Pro)

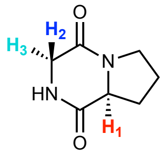

Pseudo first order rate constants for the deuteroxide catalysed exchange, k_{ex}, of the prolyl α-proton H_1 and glycyl α-protons H_2 and H_3 of c(Gly-L-Pro) were determined in $KDCO_3$ buffered D_2O solutions (pD = 10.39 – 10.83) using ^{1}H NMR spectroscopy (400 MHz).

S1.4.9.1 c(Gly-L-Pro) Prolyl α-proton

A representative set of spectra taken at three time points during the reaction at pD = 10.12 is shown in Figure S1.32. Deuterium exchange at the H_1 position on c(Gly-L-Pro) resulted in disappearance of the doublet of doublets due to H_1 at 4.25 ppm over the course of the reaction. The level of exchange was monitored relative to a tetramethylammonium deuteriosulfate internal standard, whose methyl protons (singlet, 3.14 ppm) are non-

exchangeable. The products of deuterium exchange at H_1 position on c(Gly-L-Pro) are deuterated c(Gly-L-Pro) and deuterated c(Gly-D-Pro), which are enantiomers and therefore have an equivalent chemical shift (Scheme S6). It is expected that the equilibrium concentration of each enantiomer will be identical. No changes in the integrated areas from the pyrrolidine ring protons (H_4, $H_{4'}$, H_5, $H_{5'}$ H_6 and $H_{6'}$) on c(Gly-L-Pro) and no hydrolysis were observed.

Figure S1.32: Representative ^{1}H NMR spectra at 400 MHz for the deuterium exchange reaction of c(Gly-L-Pro) (5.0 mM) in $KDCO_3$ (50% free base, 0.185 M, pD = 10.12) at 25 °C and ionic strength I = 1.0 M (KCl).

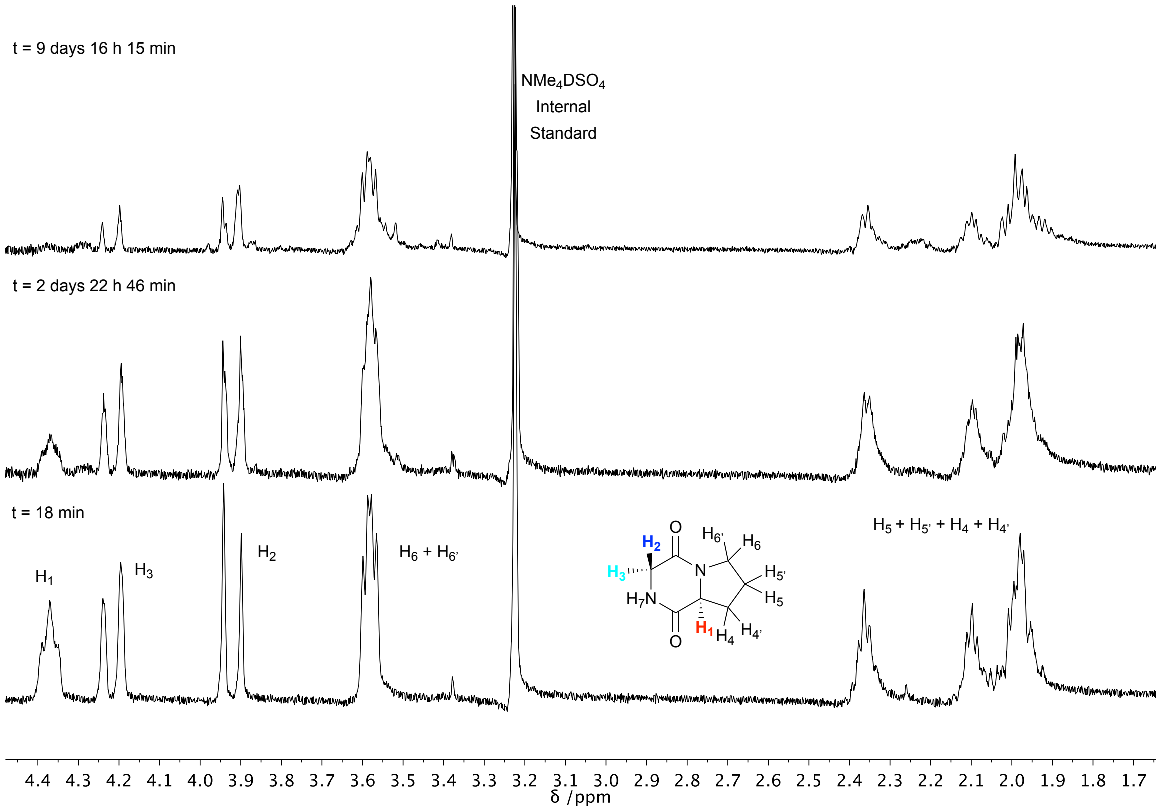

Scheme S6: Outcomes of the deuterium exchange reactions of c(Gly-L-Pro).

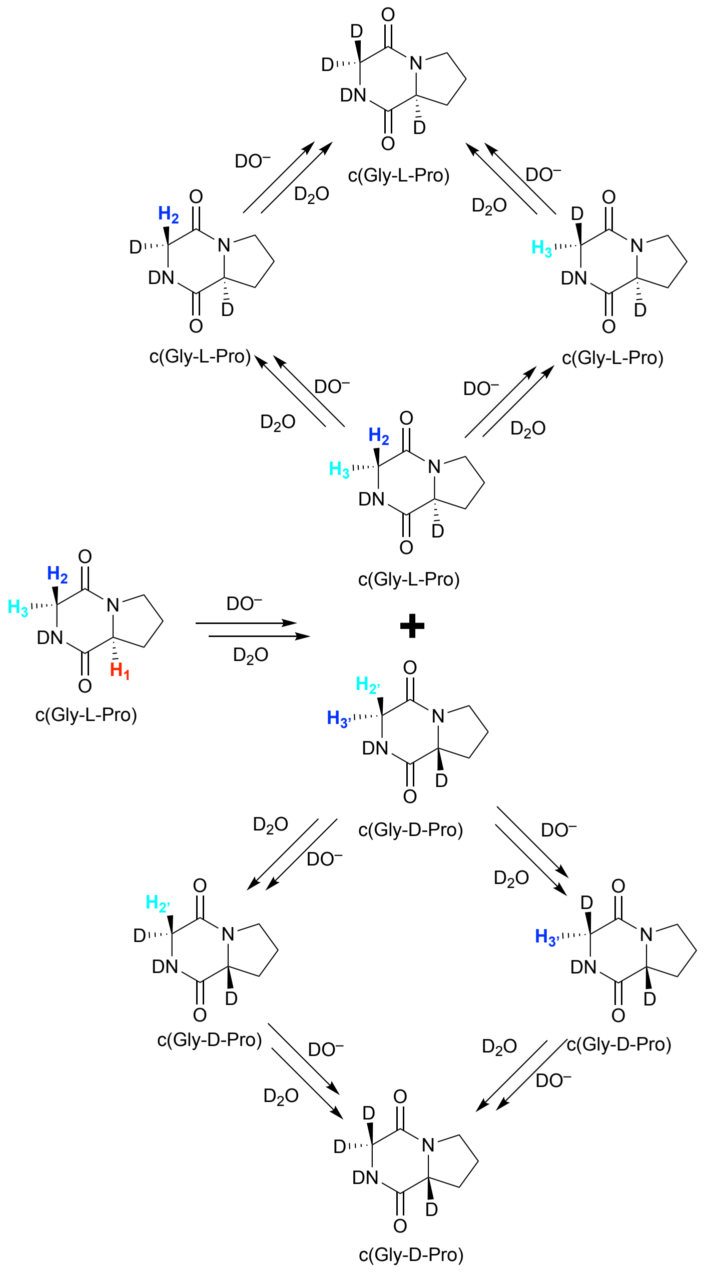

Experimentally observed first order rate constants for deuteroxide catalysed deuterium exchange, k_{ex}, of the prolyl α-proton H_1 of c(Gly-L-Pro) were determined from the fit of the fraction of protonated substrate remaining, *f*(s), for H_1 c(Gly-L-Pro) to Equation S3. Reaction data and k_{ex} values for H_1 over the p*D* range p*D* = 9.99 – 10.43 are shown in Table S1.16.

Figure S1.33 shows the dependence of k_{ex} on the concentration of deuteroxide for H_1. The fit of the reaction data to Eqn. S5 gives a value of $k_{DO} = 1.87 \times 10^{-1}$ M^{-1} s^{-1} for H_1. The fit of the data in Figure S1.33 crosses close to the origin and the lack of upward curvature at higher

concentrations of deuteroxide indicate that insignificant buffer catalysis is present for exchange of H_1.

Table S1.16: Reaction data and observed first order rate constants (k_{ex}) for the deuterium exchange of the prolyl α-proton H_1 in c(Gly-L-Pro) (5.0 mM) in $KDCO_3$ (0.185 M, pD = 10.39 – 10.83) buffered D_2O solution, I = 1.0 (KCl) and 25 ºC.

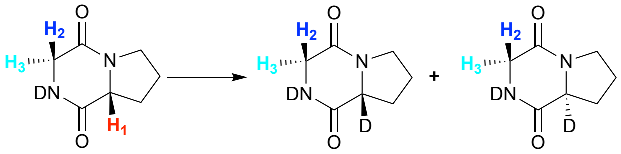

Experiment	Proton Chemical Shift /ppm	$[DO^-]$ /M	Time /s	$f(s)^a$	k_{ex} /s^{-1}
70% fb	H_1	4.13×10^{-5}	1.75×10^{3}	1.00	7.63×10^{-6}
	4.21-4.28	(pD 10.83)	7.47×10^{4}	0.57	
			1.61×10^{5}	0.28	
			2.55×10^{5}	0.15	
			6.91×10^{5}	0.03	
			7.57×10^{5}	0.03	
			8.34×10^{5}	0.03	
60% fb	H_1	2.66×10^{-5}	1.06×10^{3}	1.00	4.40×10^{-6}
	4.21-4.28	(pD 10.64)	7.54×10^{4}	0.70	
			1.67×10^{5}	0.48	
			2.55×10^{5}	0.32	
			6.90×10^{5}	0.08	
			7.57×10^{5}	0.06	
			8.36×10^{5}	0.06	
50% fb	H_1	2.04×10^{-5}	1.06×10^{3}	1.00	3.64×10^{-6}
	4.21-4.28	(pD 10.52)	8.34×10^{4}	0.73	
			1.67×10^{5}	0.52	
			2.55×10^{5}	0.37	
			6.90×10^{5}	0.13	
			7.57×10^{5}	0.09	
			8.36×10^{5}	0.08	
48% fb	H_1	1.82×10^{-5}	1.06×10^{3}	1.00	3.23×10^{-6}
	4.21-4.28	(pD 10.47)	8.34×10^{4}	0.75	
			1.67×10^{5}	0.56	

			2.55×10^5	0.43	
			6.90×10^5	0.11	
			7.58×10^5	0.11	
			8.36×10^5	0.11	
20% fb	H_1	1.59×10^{-5}	1.06×10^3	1.00	2.74×10^{-6}
	4.21-4.28	(p*D* 10.41)	8.43×10^4	0.79	
			1.67×10^5	0.63	
			2.55×10^5	0.48	
			7.39×10^5	0.14	
			8.36×10^5	0.12	
22% fb	H_1	1.52×10^{-5}	1.06×10^3	1.00	2.70×10^{-6}
	4.21-4.28	(p*D* 10.39)	8.38×10^4	0.80	
			1.67×10^5	0.64	
			2.55×10^5	0.49	
			8.36×10^5	0.12	

[a] fraction of unreacted substrate remaining at each time point.

Figure S1.33: Plot of the dependence of k_{ex} upon $[DO^-]$ for the deuterium exchange of the prolyl α-proton H_1 in c(Gly-L-Pro) (5.0 mM) in $KDCO_3$ (0.185 M, p*D* = 10.39 – 10.83) buffered D_2O solution, *I* = 1.0 (KCl) and 25 ºC.

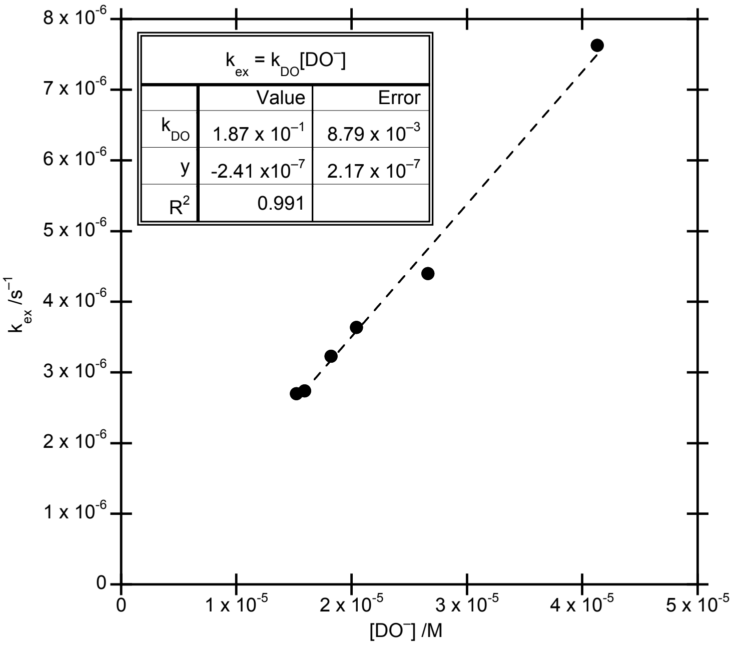

S1.4.9.2 c(Gly-L-Pro) Glycyl α-protons

Deuterium exchange at the H_2 and H_3 position on c(Gly-L-Pro) resulted in disappearance of the doublets at 3.77 ppm and 4.06 ppm, respectively, over the course of the reaction (Figure S1.32). The signals are also due to the $H_{3'}$ and $H_{2'}$ protons on c(Gly-D-Pro)[vi], respectively. As c(Gly-L-Pro) and c(Gly-D-Pro) are enantiomers, deuterium exchange at the H_2 and $H_{3'}$, and, H_3 and $H_{2'}$ protons should be equivalent. The level of exchange was monitored relative to a tetramethylammonium deuteriosulfate internal standard, whose methyl protons (singlet, 3.14 ppm) are non-exchangeable. The final products of deuterium exchange at both the H_2 and H_3 positions on c(Gly-L-Pro) are tri-deuterated c(Gly-L-Pro) (Scheme S6, top half) and tri-deuterated c(Gly-D-Pro) (Scheme S6, bottom half), which are enantiomers possessing equivalent ^{1}H NMR chemical shift.

Experimentally observed first order rate constants for deuteroxide catalysed deuterium exchange, k_{ex}, of glycyl α-proton H_2 and H_3 of c(Gly-L-Pro) were determined from the fit of the fraction of protonated substrate remaining, f(s), for H_2 c(Gly-L-Pro) to Equation S3. Reaction data and k_{ex} values for H_2 over the pD range pD = 9.99 – 10.43 are shown in Table S1.17 and Table S1.18. Figure S1.34 and 2.35 shows the dependence of k_{ex} on the concentration of deuteroxide for H_2 and H_3. The fit of the reaction data to Eqn. S5 gives values of $k_{DO} = 2.09 \times 10^{-2}$ M^{-1} s^{-1} for H_2 and $k_{DO} = 6.59 \times 10^{-2}$ M^{-1} s^{-1} for H_3.

[vi] c(Gly-D-Pro) in solution arises from interconversion of c(Gly-L-Pro) to c(Gly-D-Pro), via the faster exchange at the prolyl α-proton H_1.

Table S1.17: Reaction data and observed first order rate constants (k_{ex}) for the deuterium exchange of the glycyl α-proton H_2 in c(Gly-L-Pro) and $H_{3'}$ in c(Gly-D-Pro) (5.0 mM) in $KDCO_3$ (0.185 M, pD = 10.39 – 10.83) buffered D_2O solution, I = 1.0 (KCl) and 25 °C.

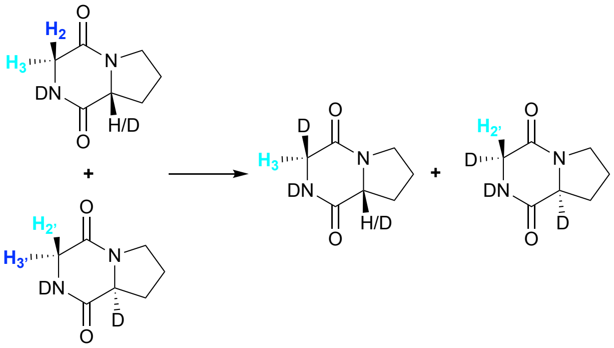

Experiment	Proton Chemical Shift /ppm	[DO⁻] /M	Time /s	f(s)[a]	k_{ex} /s^{-1}
70% fb	$H_2 + H_{3'}$	4.13×10^{-5}	1.75×10^{3}	1.00	7.91×10^{-7}
	3.75-3.79+3.80-3.83	(pD 10.43)	7.47×10^{4}	0.95	
			1.61×10^{5}	0.84	
			2.55×10^{5}	0.79	
			6.91×10^{5}	0.57	
			7.57×10^{5}	0.56	
			8.34×10^{5}	0.54	
60% fb	$H_2 + H_{3'}$	2.66×10^{-5}	1.06×10^{3}	1.00	4.36×10^{-7}
	3.75-3.79+3.80-3.83	(pD 10.24)	7.54×10^{4}	0.94	
			1.67×10^{5}	0.93	
			2.55×10^{5}	0.88	
			6.90×10^{5}	0.74	
			7.57×10^{5}	0.72	
			8.36×10^{5}	0.71	
50% fb	$H_2 + H_{3'}$	2.04×10^{-5}	1.06×10^{3}	1.00	3.28×10^{-7}
	3.75-3.79+3.80-3.83	(pD 10.12)	8.34×10^{4}	0.99	
			1.67×10^{5}	0.93	
			2.55×10^{5}	0.91	
			6.90×10^{5}	0.80	
			7.57×10^{5}	0.78	
			8.36×10^{5}	0.76	
48% fb	$H_2 + H_{3'}$	1.82×10^{-5}	1.06×10^{3}	1.00	2.91×10^{-7}

	3.75-3.79+3.80-3.83	(p*D* 10.07)	8.34×10^{4}	0.98	
			1.67×10^{5}	0.94	
			2.55×10^{5}	0.95	
			6.90×10^{5}	0.81	
			7.58×10^{5}	0.80	
			8.36×10^{5}	0.79	
20% fb	$H_2 + H_{3'}$	1.59×10^{-5}	1.06×10^{3}	1.00	2.77×10^{-7}
	3.75-3.79+3.80-3.83	(p*D* 10.01)	8.43×10^{4}	0.98	
			1.67×10^{5}	0.94	
			2.55×10^{5}	0.95	
			7.39×10^{5}	0.81	
			8.36×10^{5}	0.80	
22% fb	$H_2 + H_{3'}$	1.52×10^{-5}	1.06×10^{3}	1.00	2.24×10^{-7}
	3.75-3.79+3.80-3.83	(p*D* 9.99)	8.38×10^{4}	1.00	
			1.67×10^{5}	0.98	
			2.55×10^{5}	0.93	
			8.36×10^{5}	0.83	

[a] fraction of unreacted substrate remaining at each time point.

Table S1.18: Reaction data and observed first order rate constants (k_{ex}) for the deuterium exchange of the glycyl α-proton H_3 in c(Gly-L-Pro) and $H_{2'}$ in c(Gly-D-Pro) (5.0 mM) in $KDCO_3$ (0.185 M, pD = 10.39 – 10.83) buffered D_2O solution, I = 1.0 (KCl) and 25 ºC.

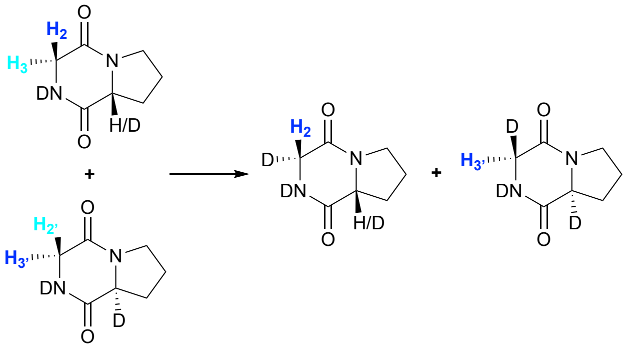

Experiment	Proton Chemical Shift /ppm	[DO⁻] /M	Time /s	f(s)[a]	k_{ex} /s^{-1}
70% fb	$H_3 + H_{2'}$	4.13×10^{-5}	1.75×10^{3}	1.00	2.75×10^{-6}
	4.05-4.09+4.09-4.13	(pD 10.43)	7.47×10^{4}	0.81	
			1.61×10^{5}	0.62	
			2.55×10^{5}	0.48	
			6.91×10^{5}	0.16	
			7.57×10^{5}	0.14	
			8.34×10^{5}	0.12	
60% fb	$H_3 + H_{2'}$	2.66×10^{-5}	1.06×10^{3}	1.00	1.68×10^{-6}
	4.05-4.09+4.09-4.13	(pD 10.24)	7.54×10^{4}	0.84	
			1.67×10^{5}	0.74	
			2.55×10^{5}	0.64	
			6.90×10^{5}	0.31	
			7.57×10^{5}	0.29	
			8.36×10^{5}	0.27	
50% fb	$H_3 + H_{2'}$	2.04×10^{-5}	1.06×10^{3}	1.00	1.38×10^{-6}
	4.05-4.09+4.09-4.13	(pD 10.12)	8.34×10^{4}	0.89	
			1.67×10^{5}	0.77	
			2.55×10^{5}	0.68	
			6.90×10^{5}	0.39	
			7.57×10^{5}	0.37	
			8.36×10^{5}	0.32	
48% fb	$H_3 + H_{2'}$	1.82×10^{-5}	1.06×10^{3}	1.00	1.22×10^{-6}

	4.05-4.09+4.09-4.13	(p*D* 10.07)	8.34×10^{4}	0.90	
			1.67×10^{5}	0.80	
			2.55×10^{5}	0.73	
			6.90×10^{5}	0.42	
			7.58×10^{5}	0.41	
			8.36×10^{5}	0.38	
20% fb	$H_3 + H_{2'}$	1.59×10^{-5}	1.06×10^{3}	1.00	1.06×10^{-6}
	4.05-4.09+4.09-4.13	(p*D* 10.01)	8.43×10^{4}	0.90	
			1.67×10^{5}	0.83	
			2.55×10^{5}	0.76	
			7.39×10^{5}	0.46	
			8.36×10^{5}	0.42	
22% fb	$H_3 + H_{2'}$	1.52×10^{-5}	1.06×10^{3}	1.00	1.00×10^{-6}
	4.05-4.09+4.09-4.13	(p*D* 9.99)	8.38×10^{4}	0.93	
			1.67×10^{5}	0.85	
			2.55×10^{5}	0.77	
			8.36×10^{5}	0.43	

[a] fraction of unreacted substrate remaining at each time point.

Figure S1.34: Plot of the dependence of k_{ex} upon [DO^-] for the deuterium exchange of the glycyl α-proton H_2 in c(Gly-L-Pro) (5.0 mM) in $KDCO_3$ (0.185 M, p*D* = 10.39 – 10.83) buffered D_2O solution, *I* = 1.0 (KCl) and 25 ºC.

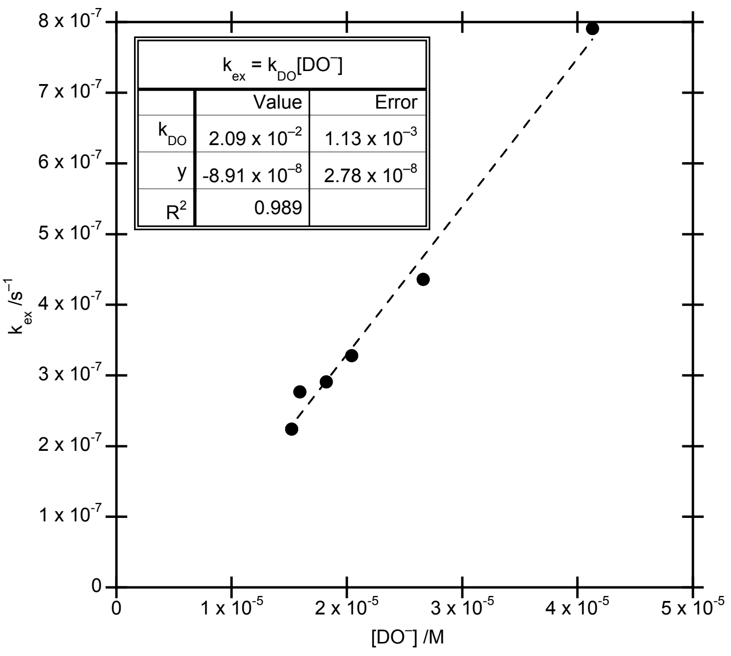

Figure S1.35: Plot of the dependence of k_{ex} upon $[DO^-]$ for the deuterium exchange of the glycyl α-proton H_3 in c(Gly-L-Pro) (5.0 mM) in $KDCO_3$ (0.185 M, pD = 10.39 – 10.83) buffered D_2O solution, I = 1.0 (KCl) and 25 ºC.

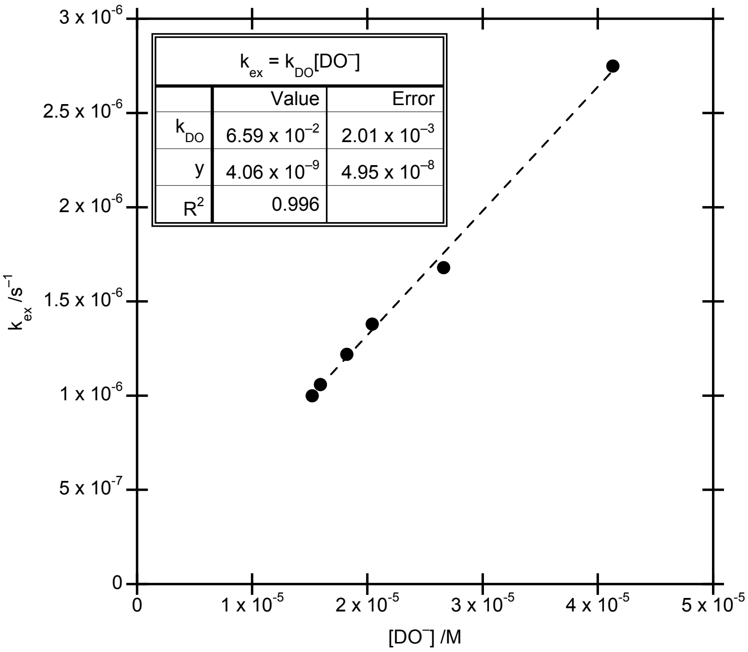

Figure S1.36: NOESY spectrum for c(Gly-L-Pro) in D_2O. The prolyl α-proton (H_1, Main Text Figure 3) is at 4.17 ppm, one glycyl α-proton (H_3, Main Text Figure 3) is at 4.03 ppm and one glycyl α-proton (H_2, Main Text Figure 3) is at 3.74 ppm.

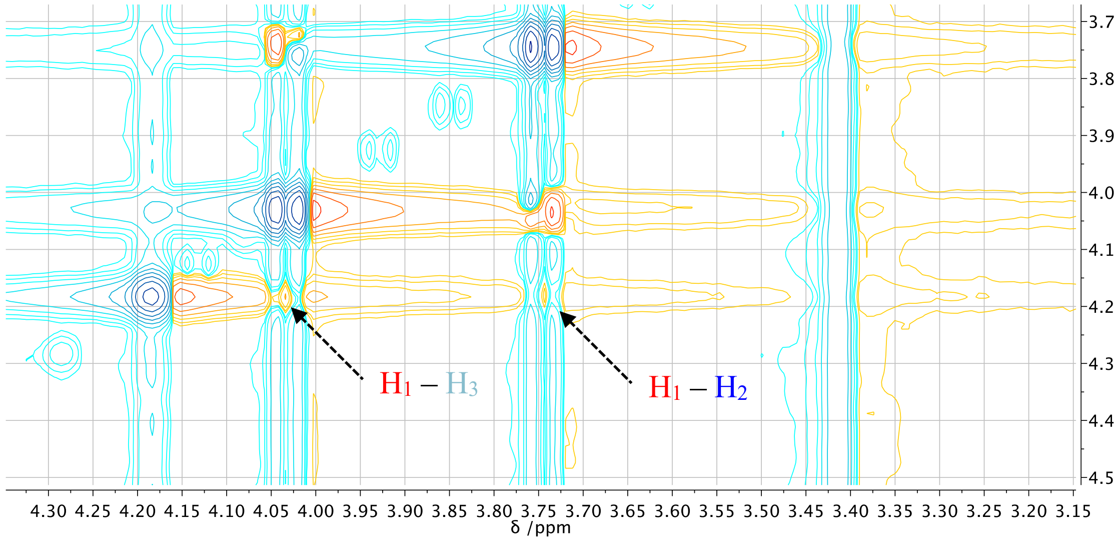

S1.4.10 Hydrogen-deuterium exchange reactions of c(Gly-Gly)

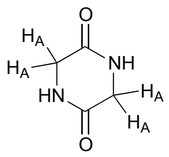

Pseudo first order rate constants for the deuteroxide catalysed exchange, k_{ex}, of the glycyl α-proton H_A of c(Gly-Gly) were determined in 3-chloroquinuclidine buffered D_2O solutions (pD = 9.35 – 10.82) using ^{1}H NMR spectroscopy (500 MHz).

A representative set of spectra taken at three time points during the reaction at pD = 10.19 is shown in Figure S1.37. Deuterium exchange at the H_A position on c(Gly-Gly) resulted in disappearance of the H_A singlet at 4.15 ppm over the course of the reaction. The level of exchange was monitored relative to a sodium trimethylsilylpropyl sulfonate internal standard, whose methyl protons (singlet, 0.11 ppm) are non-exchangeable. The product of deuterium exchange at the H_A position on c(Gly-Gly) is deuterated c(Gly-Gly) (Scheme S7). As deuterium exchange progresses, an additional peak from the H_A protons appears at 4.62 ppm. This upfield peak is a triplet (albeit poorly resolved in Figure S1.37) due to the coupling between the D and H in the singly exchanged α-CHD product.[4] It can be assumed the rate constants for H/D exchange of $C(H_A)_2$ and CH_AD are equivalent. A small but appreciable amount of the hydrolysis product, dipeptide Gly-Gly, was also observed at 3.89 ppm.

Figure S1.37: Representative ^{1}H NMR spectra at 500 MHz for the deuterium exchange reaction of c(Gly-Gly) (20.0 mM) in 3-chloroquinuclidine (0.15 M, 70% fb, pD = 10.19) at 25 °C and I = 1.0 M (KCl).

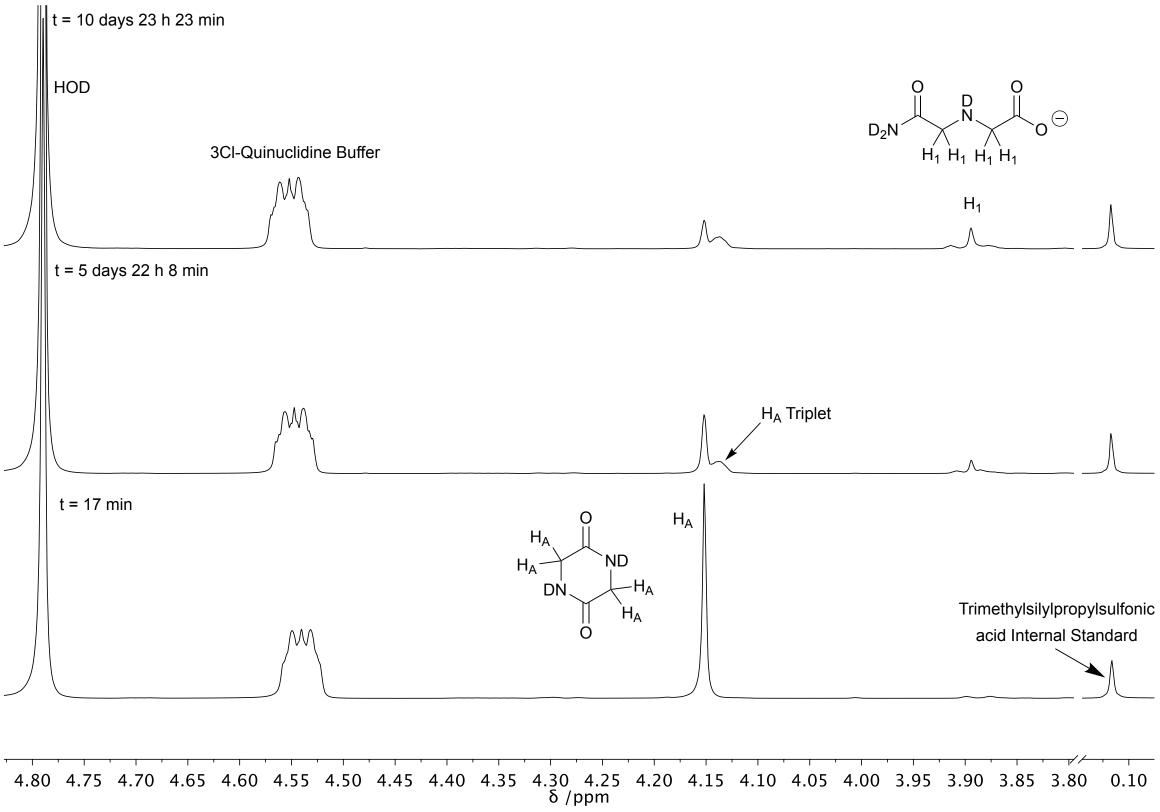

Scheme S7: Outcomes of the deuterium exchange reactions of c(Gly-Gly).

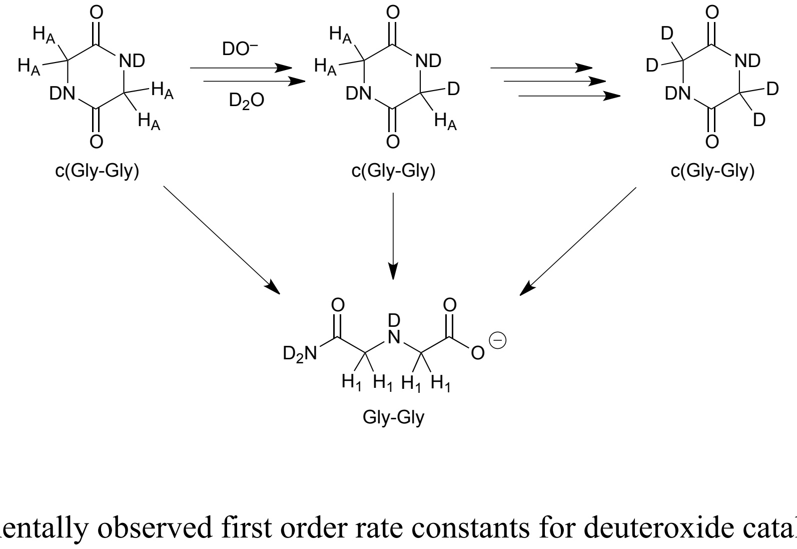

Experimentally observed first order rate constants for deuteroxide catalysed deuterium exchange, k_{ex}, of glycyl α-proton H_A c(Gly-Gly) were determined from the fit of Eqn. S3 to the fraction of protonated substrate remaining, f(s), for H_A c(Gly-Gly). Reaction data and k_{ex} values

for H_A over the p*D* range p*D* = 9.35 – 10.82 are shown in Table S1.19. Figure S1.38 shows the dependence of k_{ex} on the concentration of deuteroxide for H_A. The fit of the reaction data to Equation S5 to the data gives a value of $k_{DO} = 6.33 \times 10^{-2}$ M^{-1} s^{-1} for H_A.

Table S1.19: Reaction data and observed first order rate constants (k_{ex}) for the deuterium exchange of the glycyl α-proton H_A in c(Gly-Gly) (20.0 mM) in 3-chloroquinuclidine (0.15 M, p*D* = 10.19 – 10.82) at 25 °C and *I* = 1.0 M (KCl).

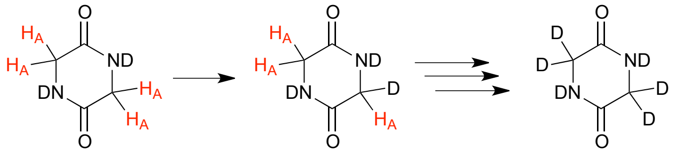

Experiment	Proton Chemical Shift /ppm	[DO⁻] /M	Time /s	*f*(s)[a]	k_{ex} /s^{-1}
90%fb	4.01-4.05	1.02×10^{-4}	1.06×10^{3}	1.00	6.81×10^{-6}
	H_A	(p*D* 10.82)	1.08×10^{5}	0.44	
			1.69×10^{5}	0.31	
			2.79×10^{5}	0.16	
			3.59×10^{5}	0.11	
			4.26×10^{5}	0.08	
			5.11×10^{5}	0.05	
			5.93×10^{5}	0.03	
			6.85×10^{5}	0.03	
70%fb	4.01-4.05	2.29×10^{-5}	1.06×10^{3}	1.00	2.04×10^{-6}
	H_A	(p*D* 10.17)	7.02×10^{4}	0.87	
			3.43×10^{5}	0.49	
			5.12×10^{5}	0.35	
			5.91×10^{5}	0.30	
			6.76×10^{5}	0.25	
			8.67×10^{5}	0.18	
			9.48×10^{5}	0.15	
50%fb	4.01-4.05	6.04×10^{-6}	1.06×10^{3}	1.00	7.68×10^{-7}
	H_A	(p*D* 9.59)	7.48×10^{4}	0.96	
			1.84×10^{5}	0.87	
			4.36×10^{5}	0.70	
			5.90×10^{5}	0.63	
			6.75×10^{5}	0.60	

			7.66×10^{5}	0.55	
			1.14×10^{6}	0.43	
30%fb	4.01-4.05	3.47×10^{-6}	1.06×10^{3}	1.00	4.59×10^{-7}
	H_A	(p*D* 9.35)	8.29×10^{4}	0.96	
			2.72×10^{5}	0.88	
			3.38×10^{5}	0.86	
			4.21×10^{5}	0.83	
			5.07×10^{5}	0.79	
			6.75×10^{5}	0.73	
			9.40×10^{5}	0.65	

[a] fraction of unreacted substrate remaining at each time point.

Figure S1.38: Plot of the dependence of k_{ex} upon [DO⁻] for the deuterium exchange of the glycyl α-proton H_A in c(Gly-Gly) (20.0 mM) in 3-chloroquinuclidine (0.15 M, p*D* = 10.19 – 10.82) at 25 °C and *I* = 1.0 M (KCl).

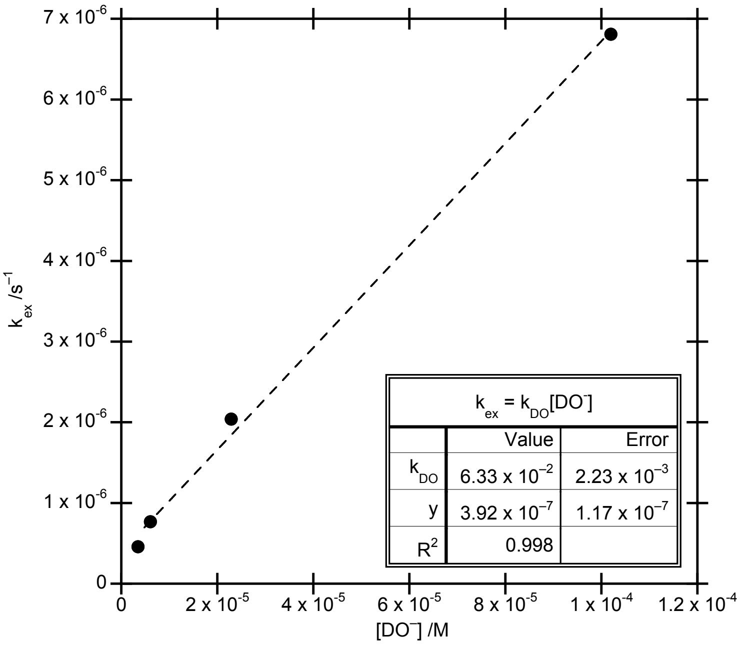

S1.4.11 Hydrogen-deuterium exchange reactions of c(L-Ala-L-Ala)

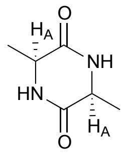

Pseudo first order rate constants for the deuteroxide catalysed exchange, k_{ex}, of the alanyl α-proton H_A of c(L-Ala-L-Ala) were determined in 3-chloroquinuclidine buffered D_2O solutions (pD = 9.35 – 10.81) using ^{1}H NMR spectroscopy (500 MHz).

A representative set of spectra taken at three time points during the reaction at pD = 10.17 is shown in Figure S1.39. Deuterium exchange at the H_A position on c(L-Ala-L-Ala) resulted in disappearance of the H_A quartet at 4.27 ppm over the course of the reaction. The level of exchange was monitored relative to a trimethylsilylpropyl sulfonate internal standard, whose methyl protons (singlet, 0.11 ppm) are non-exchangeable. The products of deuterium exchange at the H_A position on c(L-Ala-L-Ala) are deuterated c(L-Ala-L-Ala) and c(D-Ala-L-Ala) (Scheme S8). Consequently, ^{1}H NMR spectra over the course of the reaction showed the disappearance of the doublet corresponding to the methyl protons H_B at 1.58 ppm and the appearance of a broad singlet for the analogous methyl H_2 at 1.56 ppm (Figure S1.39).

Figure S1.39: Representative ^{1}H NMR spectra at 500 MHz for the deuterium exchange reaction of c(L-Ala-L-Ala) (20.0 mM) in 3-chloroquinuclidine (0.15 M, 70% fb, pD = 10.17) at 25 °C and I = 1.0 M (KCl).

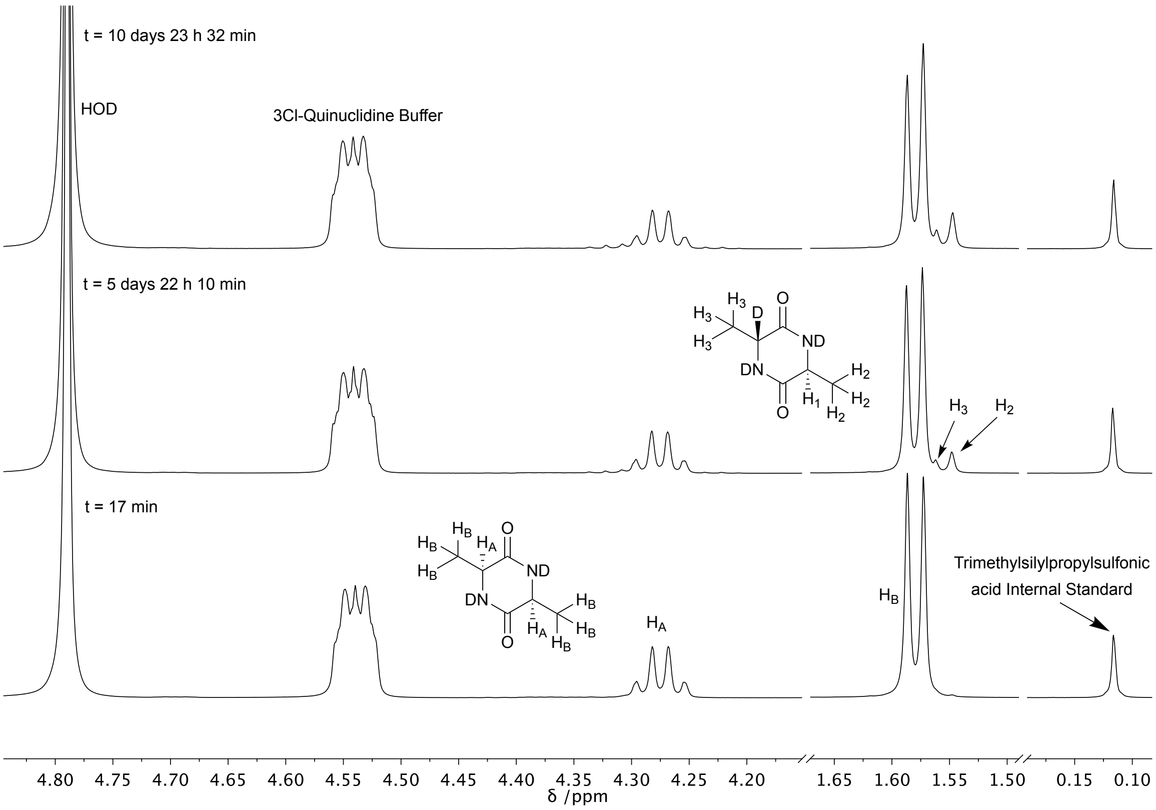

Scheme S8: Outcomes of the deuterium exchange reactions of c(L-Ala-L-Ala).

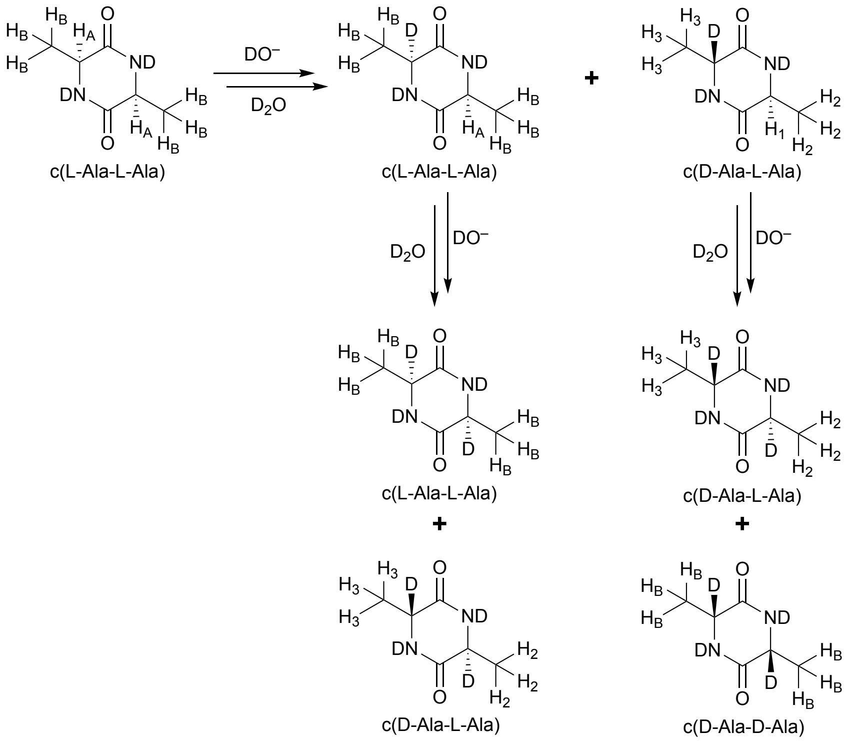

Experimentally observed first order rate constants for deuteroxide catalysed deuterium exchange, k_{ex}, of the alanyl α-proton H_A c(L-Ala-L-Ala) were determined from the fit of the fraction of protonated substrate remaining, *f*(s), for H_A c(L-Ala-L-Ala) to Eqn. S3. Reaction data and k_{ex} values for H_A over the p*D* range p*D* = 9.35 – 10.81 are shown in Table S1.20. Figure S1.40 shows the dependence of k_{ex} on the concentration of deuteroxide for H_A. A value of k_{DO} = 7.80 × 10^{-3} M^{-1} s^{-1} for H_A was obtained.

Table S1.20: Reaction data and observed first order rate constants (k_{ex}) for the deuterium exchange of the alanyl α-proton H_A in c(L-Ala-L-Ala) (20.0 mM) in 3-chloroquinuclidine (0.15 M, p*D* = 9.35 – 10.81), at 25 °C and *I* = 1.0 M (KCl).

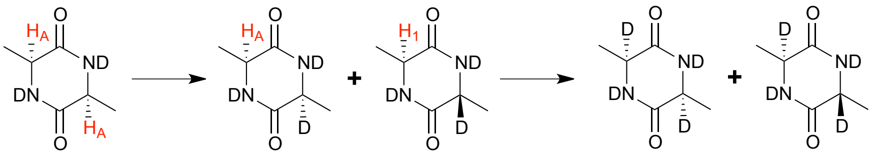

Experiment	Proton Chemical Shift ppm	[DO⁻] /M	Time /s	f(s)[a]	k_{ex} /s^{-1}
90 % fb	4.11-4.17	1.00 × 10^{-4}	1.06 × 10^{3}	1.00	8.52 × 10^{-7}
	H_A	(p*D* 10.81)	1.08 × 10^{5}	0.91	
			1.68 × 10^{5}	0.86	
			2.79 × 10^{5}	0.79	
			3.58 × 10^{5}	0.74	
			4.26 × 10^{5}	0.70	
			5.11 × 10^{5}	0.65	
50 % fb	4.11-4.17	6.32 × 10^{-6}	1.06 × 10^{3}	1.00	1.44 × 10^{-7}
	H_A	(p*D* 9.61)	7.49 × 10^{4}	0.97	
			1.84 × 10^{5}	0.97	
			4.36 × 10^{5}	0.95	
			5.90 × 10^{5}	0.92	
			6.75 × 10^{5}	0.91	
			8.52 × 10^{5}	0.89	
			1.14 × 10^{6}	0.84	
30 % fb	4.11-4.17	3.47 × 10^{-6}	1.06 × 10^{3}	1.00	7.77 × 10^{-8}
	H_A	(p*D* 9.35)	8.27 × 10^{4}	1.00	
			2.72 × 10^{5}	0.98	
			3.38 × 10^{5}	0.98	

4.21×10^5	0.96
5.08×10^5	0.96
6.76×10^5	0.95
9.41×10^5	0.93

[a] fraction of unreacted substrate remaining at each time point.

Figure S1.40: Plot of the dependence of k_{ex} upon $[DO^-]$ for the deuterium exchange of the alanyl α-proton H_A in c(L-Ala-L-Ala) (20.0 mM) in 3-chloroquinuclidine (0.15 M, pD = 9.35 – 10.81), at 25 °C and I = 1.0 M (KCl).

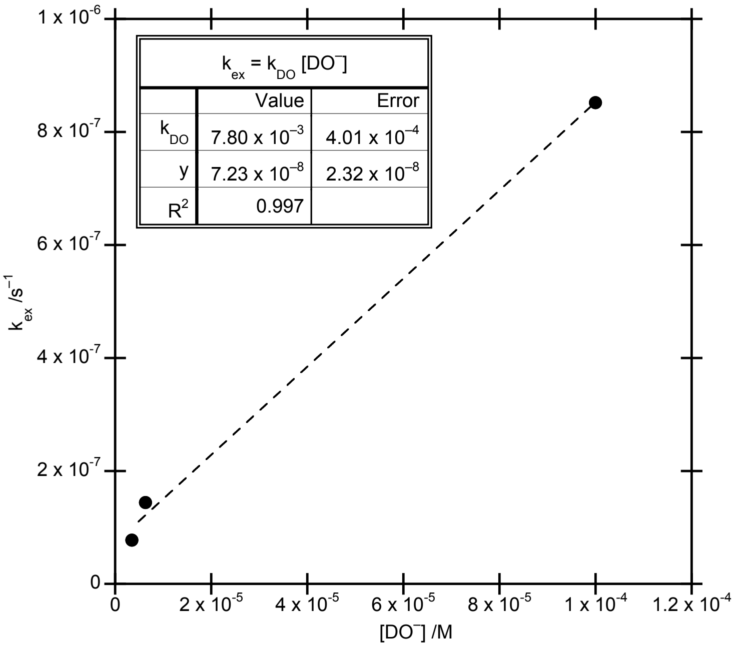

S2 Hydrogen Deuterium Exchange studies on Triketopiperazines

S2.1 General Instrumentation

NMR: Kinetic NMR spectra were recorded on an Oxford Varian Unity Inova 500 MHz NMR spectrometer. NMR samples were prepared in 60 : 40 D_2O : d_3-acetonitrile. All chemical shifts are reported relative to the residual solvent peak of δ_H 4.79 ppm for D_2O.

S2.2 Materials

NMR Solvents: d_2-Deuterium oxide (99.9 atom % D) and d_3-acetonitrile (99.8 atom % D) were purchased from Goss Scientific. Deuterium chloride (99 atom % D, 35 wt %) was purchased from Sigma-Aldrich. A stock solution of 2 M potassium chloride was prepared by dissolving solid potassium chloride in D_2O. A stock solution of deuterium chloride was prepared by a dissolving concentrated deuterium chloride solution in D_2O and then titrating against volumetric sodium hydroxide solutions to determine the concentration of DCl.

Kinetic Experiments: Potassium acetate, potassium chloride and tetramethylammonium hydrogensulfate were purchased from Sigma Aldrich.

S2.3 Synthesis of Triketopiperazines

Synthetic procedure for the glycyl TKP are found in A. Cabanillas *et al.* ***Chem. Sci.***, 2015, **6**, 1350-1354. Synthetic procedures for the prolyl TKP are found in M. Rees. *Org. Lett.*, 2017, **19**, 1338-1341.

S2.4 Kinetic Experiments

The methodology for kinetic experiments using the TKPs was closely similar to that used for the DKP kinetic experiments. The hydrogen deuterium exchange was monitored in aqueous acetate buffers with a 40 % d_3-MeCN co-solvent in the p*D* range 4.76 – 6.29 at 25 °C and ionic strength, I = 0.06 for the prolyl TKP and I = 0.2 for the glycyl TKP.

S2.4.1 Solution Preparation

Acetic acid (AcOH/KOAc) buffer solutions were prepared from dissolving solid potassium acetate in 60 : 40 D_2O : d_3-acetonitrile. Deuterium chloride solution was used to adjust the percentage of free base form of the buffer. A 2 M potassium chloride solution was used to adjust the ionic strength of the solution.
A tetramethylammonium deuteriosulfate internal standard solution was prepared with D_2O.

S2.4.2 Measurement of pH

The pH of the buffered solutions was determined using a MeterLab™ PHM 290 p*H*-Stat Controller equipped with a radiometer combination electrode filled with saturated KCl solution at 25 ºC. The pH meter was calibrated with pH 4 phthalate and pH 7 potassium phosphate solutions. The p*D* values were calculated by adding 0.4 to the pH meter reading.[2] It was necessary to correct the p*D* values as the solutions contained 40 vol% d_3-MeCN. Bosch *et al* have shown that the difference between the pH of a solution containing 40 vol% MeCN and the pH recorded with a glass electrode containing 100 vol% H_2O is $\delta = -0.175$ pH units; this correction was added to the observed p*D* [p*D*(obs)] values to give the corrected p*D* [p*D*(corr)] values for the 60 : 40 D_2O : d_3-MeCN solution (Eqn. S9).[5]

$$\mathrm{p}D(\mathrm{corr}) = \mathrm{p}D(\mathrm{obs}) + \delta = \mathrm{p}D(\mathrm{obs}) - 0.175 \quad \text{(Eqn. S9)}$$

The autoprotolysis constant of water, K_s, varies with solvent composition. Aslan has shown that for a 60 : 40 H_2O : MeCN solution at 25 ºC and $I = 0.1$ ($NaClO_4$), the autoprotolysis constant of water is pK_s(H_2O : MeCN) = 14.74.[6] To convert this into the autoprotolysis constant for D_2O in a 60 : 40 H_2O : MeCN, pK_s(D_2O : MeCN), we assumed that the ratio of $K_s(H_2O)$ / $K_s(D_2O) = 10^{-14} / 10^{-14.87} = 7.40$ was the same for a H_2O / D_2O acetonitrile mixed solvent, i.e. K_s(H_2O : MeCN) / K_s(D_2O : MeCN) = 7.40, and therefore taking pK_s(H_2O : MeCN) = 14.74 from Aslan above the pK_s(D_2O : MeCN) = 15.61. It is assumed that the use of d_3-MeCN rather than MeCN will not substantially affect this value.

In order to determine the concentration of deuteroxide present in a 60 : 40 D_2O : d_3-MeCN solution Eqn. S10 was used. A previously determined activity coefficient for the deuteroxide ion (γ_{DO}) in a 2 : 1 D_2O : d_3-MeCN solution of $\gamma_{DO} = 1.56$ was used in conjunction

with the pD(corr) and pK_s(D_2O : MeCN) values from above to determine the concentration of deuteroxide ion.[7]

$$[DO^-] = \frac{10^{[pD(corr) - pK_s(D_2O\,:\,MeCN)]}}{\gamma_{OD}} \qquad \text{(Eqn. S10)}$$

S2.4.3 NMR experimental conditions

The hydrogen deuterium exchange experiments were usually carried out on an Oxford Varian Unity Inova 500 MHz NMR spectrometer with a thermostated probe. A pulse sequence with a 20 s relaxation delay, an acquisition time of 4 s and a 90 º pulse angle. A total of 32 transients were taken for each spectrum (a total acquisition time of ~ 12 min 45 s). The measurement times, *t*, for the exchange experiments were calculated from the mid-point of the acquisition. ^{1}H NMR spectral baselines were subject to a first-order drift correction before integration of the peak areas. The integration of substrate peak areas was compared to the peaks of the tetramethylammonium deuteriosulfate internal standard with an arbitrary value of 1000 for the integration of the internal standard.

To initiate an exchange reaction the TKP was initially dissolved in a small volume (< 20 μL) of d_3-acetonitrile. 2.0 mL of the buffer solution was then added followed by the internal standard solution. 0.7 mL of this solution was then placed within an NMR tube and this was immediately placed within the thermostated NMR spectrometer. The spectrometer was then reshimmed and the exchange was subsequently monitored. Periodically throughout the experiment ~ 4 acquisitions the probe was reshimmed to correct for any drift in the magnetic field. The remaining 1.3 mL of the solution was used to monitor the pH of the solution throughout the exchange experiment. Changes of less than ± 0.03 units was observed in the pH of the solutions between the beginning and end of the experiments.

S2.4.3.1 Analysis of experimental error

There are several possible sources of experimental error:

- Temperature variation: The deuterium exchange experiments were carried out on a 500 MHz NMR spectrometer that was thermostated to 25 ± 1 °C. Thus the impact of the variation in temperature is expected to be minimal.

- Variation in pD: Acetate buffers were used to maintain a constant pD. Little to no changes in pD (usually < 0.04) were observed for reactions over the same timescale of the reaction.
- Initial values at t = 0 s: the integrals used for the t = 0 values were obtained from data acquired ~ 4 minutes after initiation of the exchange experiment. The error introduced due to this time delay in acquisition is negligible as the exchange experiments typically took >41 min to complete.

Overall the sources of experimental errors were minimised and their effect on the results of the deuterium exchange experiments is therefore believed to be small.

R^2 values of ~0.99 were found for the majority of fits of the data to an exponential first order decay.

S2.4.4 Deuterium exchange reactions of TKPs

Scheme S9: Glycyl and prolyl TKP studied.

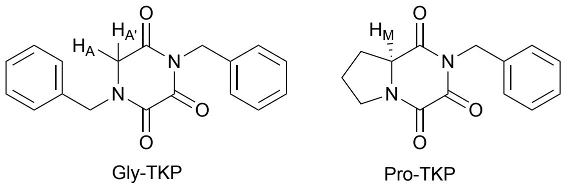

The deuterium exchange reactions for a Glycyl TKP and a Prolyl TKP were monitored by ^{1}H NMR spectroscopy in buffered potassium acetate solutions in D_2O. Owing to the poor solubility of both TKPs in D_2O, deuterated acetonitrile (d_3-MeCN) was employed as a co-solvent (40 % v/v). Exchange reactions were monitored in a thermostated 500 MHz NMR spectrometer at 25 ºC. The ionic strength was maintained at I = 0.06 for Glycyl TKP and I = 0.2 for Prolyl TKP with the lower ionic strength for the former due its lower solubility as a result of having two N-benzyl substituents. Due to lower quantities of both TKPs available for study, exchange reactions were monitored in sealed NMR tubes rather than sampling larger reaction volumes. From these data, observed first order rate constants for deuterium exchange, k_{ex} (s^{-1}), second order rate constants for buffer catalysed exchange, k_{A-} (M^{-1} s^{-1}), and second order rate constants for deuteroxide catalysed exchange, k_{DO} (M^{-1} s^{-1}), were determined. Due to the lability of the C5-proton towards deuterium exchange, these reactions were conducted at relatively low pD values (pD 4.76 – 6.29) as rapid exchange and parallel hydrolysis were

observed at higher p*D* values. By contrast, corresponding DKP exchange reactions were monitored at p*D* 9.35 – 10.98.

The deuterium exchange reaction may potentially be either acid or base catalysed in acetic acid buffers;[vii] both routes are shown in Scheme S9. Both routes are irreversible as the H^+ is diluted substantially in bulk solvent. For the analogous buffer catalysed routes, D_3O^+ and DO^- may be replaced by the general acids and bases, DA and A^-, respectively.

[vii] Solvent catalysed exchange is omitted here because it was negligible for the TKPs (see section S2.4.6).

Scheme S9: a) Acid and b) base catalysed routes for deuterium exchange reactions involving Glycyl TKP. X = H or D.

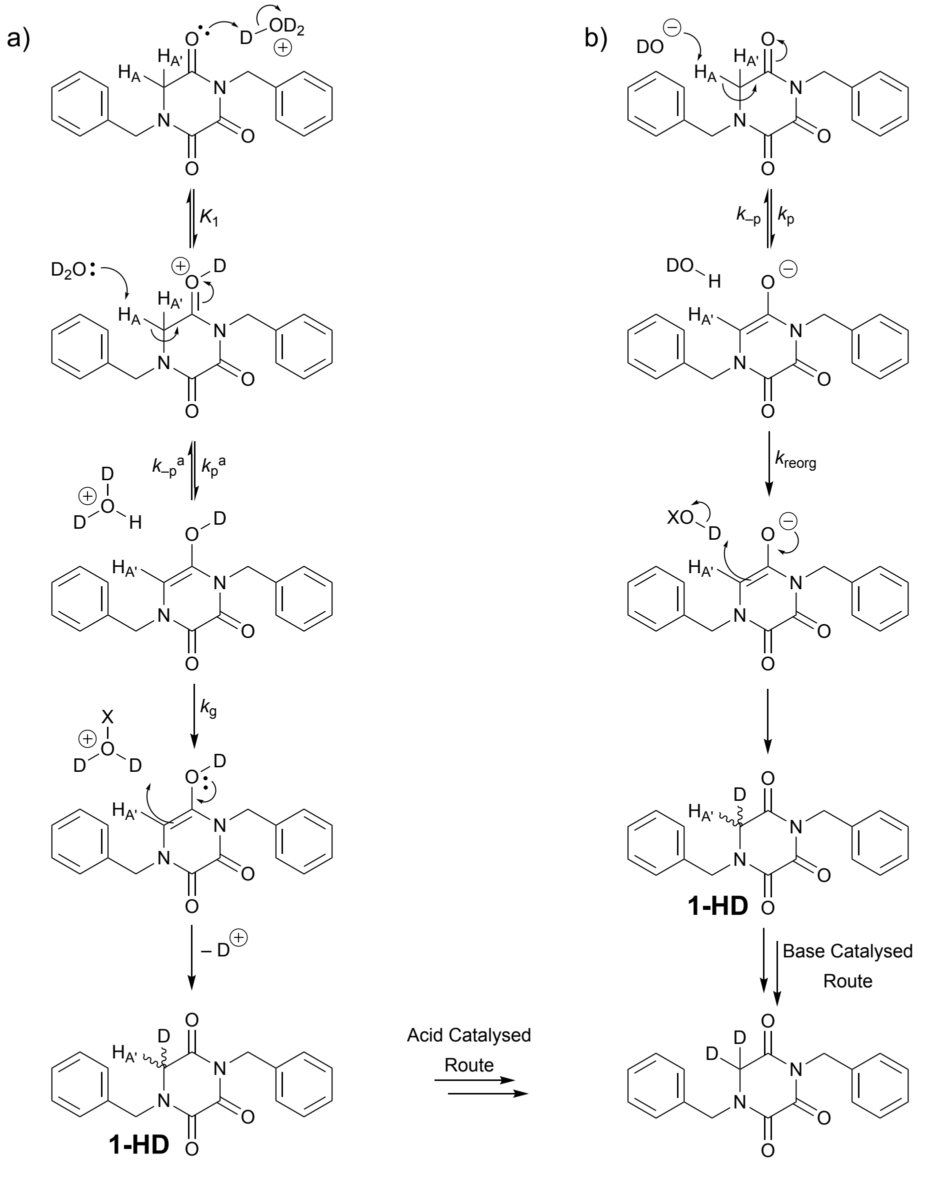

The observed first order rate constant for deuterium exchange, k_{ex}, could be determined by ^{1}H NMR spectroscopy by following the disappearance of signal from the acidic proton(s) (H_A and $H_{A'}$) over time (Eqn. S11).

$$-\frac{d[CH_AH_{A'}]}{dt} = k_{ex}[CH_AH_{A'}] \qquad \text{(Eqn. S11)}$$

The k_{ex} value potentially is comprised of both specific and general catalysis components. Reactions which are specific acid or base catalysed show a kinetic dependence on only $[D_3O^+]$ or $[DO^-]$, respectively. Reactions additionally involving general acid and base

catalysis show a kinetic dependence upon both [D_3O^+] and [DA], or, [DO^-] and [A^-], respectively. If the acid catalysed route in Scheme S9 is followed then the first order rate constant for exchange, k_{ex}, may be described by Eqn. S12.

$$k_{\mathrm{ex}} = k_{\mathrm{D_2O}} + k_{\mathrm{D+}}[\mathrm{D^+}] + k_{\mathrm{DA}}[\mathrm{DA}] \qquad \text{(Eqn. S12)}$$

where k_{D2O} is the second order rate constant for the solvent promoted exchange, k_{D+} is the second order rate constant for D^+ catalysed exchange and k_{DA} is the second order rate constant for buffer acid catalysed exchange by DA. For the base catalysed exchange route in Scheme S9, k_{ex} may be described by Eqn. S13.

$$k_{\mathrm{ex}} = k_{\mathrm{D_2O}} + k_{\mathrm{DO}}[\mathrm{DO^-}] + k_{\mathrm{A-}}[\mathrm{A^-}] \qquad \text{(Eqn. S13)}$$

where k_{DO} is the second order rate constant for deuteroxide-catalysed exchange and k_{A-} is the second order rate constant for buffer base-catalysed exchange by A^-. Overall, the observed rate constants for exchange, k_{ex}, were observed to increase with p*D* (Table S2.1), thus Eqn. S13 is relevant in this case.

Table S2.1: k_{ex} values for deuterium exchange in potassium acetate and deuterium chloride solutions for 6 mM glycyl TKP (I = 0.06 (KCl)) and 6 mM prolyl TKP (I = 0.2 (KCl)) with 40 % d_3-MeCN at 25 ºC.

TKP	Solution[a]	p*D*	k_{ex} / s^{-1}
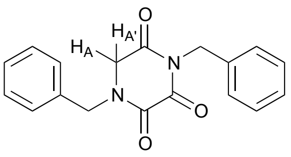	50 % fb 0.05 M KOAc	5.99	3.69×10^{-4}
	30 % fb 0.05 M KOAc	5.67	2.04×10^{-4}
	15 % fb 0.05 M KOAc	5.49	1.65×10^{-4}
	10 % fb 0.05 M KOAc	4.94	8.50×10^{-5}
	5 % fb 0.05 M KOAc	4.40	2.98×10^{-5}
	0.025 M DCl	1.80	25.1×10^{-7}
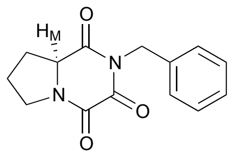	30 % fb 0.05 M KOAc	5.54	6.18×10^{-4}
	15 % fb 0.05 M KOAc	4.77	1.76×10^{-4}
	5 % fb 0.05 M KOAc	4.35	4.64×10^{-5}
	0.025 M DCl	1.85	1.25×10^{-6}

[a] %fb = % base form of the buffer.

S2.4.5 First order rate constants for deuterium exchange, k_{ex}, of Glycyl TKP

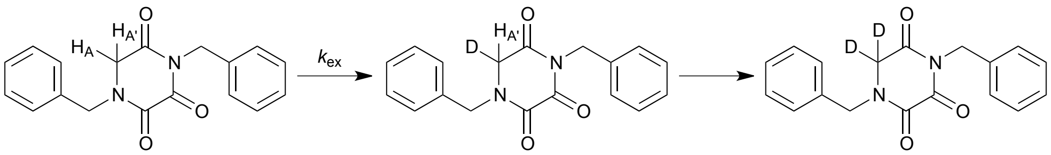

In order to assess the significance of the $k_{A-}[A^-]$ term for the Glycyl TKP, a series of exchange reactions were performed in potassium acetate buffered solutions with an identical buffer ratio (75 % free base) but varying total concentration of buffer (0.025 M – 0.05 M). The identical buffer ratio maintains a consistent p*D* between solutions and thus kept the contribution of the $k_{DO}[DO^-]$ term of Eqn. S13 as constant as possible. Thus any alterations in the rate constant of exchange will be due to the buffer component.

First order rate constants for deuterium exchange, k_{ex}, were determined by ^1H NMR spectroscopy and a representative set of spectra are shown in Figure S2.1. The exchangeable α-protons H_A and $H_{A'}$ appear as a singlet at 4.76 ppm just upfield of the HOD solvent peak. As deuterium exchange progresses, an additional peak from the α-protons appears at 4.74 ppm. This upfield peak is a triplet (albeit poorly resolved in Figure S2.1) that appears due to the coupling between the D and H of α-CHD.[4] The methylene protons from the benzyl methylene protons, H_C and H_D, appear as singlets at 5.10 ppm and 5.38 ppm, respectively, while the aromatic protons appear at 7.71 – 7.86 ppm.

Figure S2.1: Representative ^{1}H NMR spectra at 500 MHz for the hydrogen deuterium exchange of glycyl TKP (3.29 mM) in acetic acid buffer (0.025 M, 75 % fb) with 40 % d_3-MeCN co-solvent at p*D* = 6.28, *I* = 0.06 (KCl) and 25 ºC.

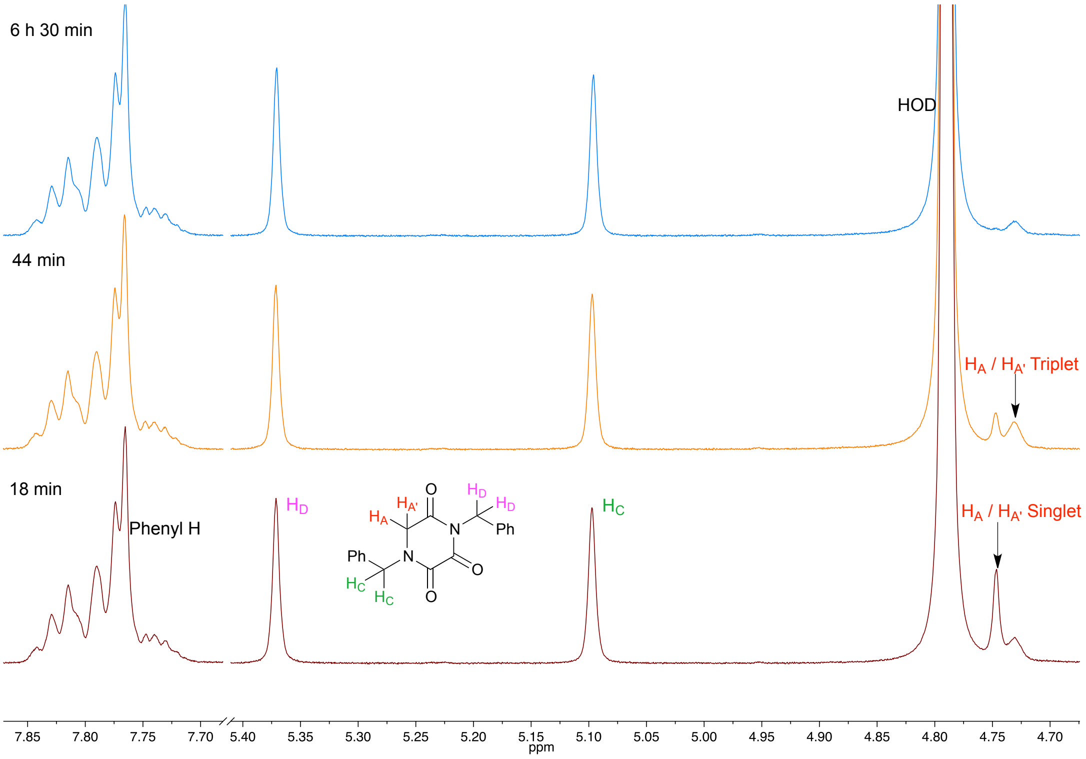

The integrated area of the H_A and $H_{A'}$ singlet ($A_{HA/HA'}$) decreased relative to the methylene peaks (A_{CH2}) and the fraction of unexchanged substrate *f*(s) was determined according to Eqn. S14. No parallel reactions were observed during the course of the exchange reaction and therefore it was acceptable to use the methylene peaks of the N-benzyl substituents as references to monitor the disappearance of H_A and $H_{A'}$. The observed first order rate constants for deuterium exchange, k_{ex}, were determined from a fitting of Eqn. S15 to a plot of *f*(s) vs. time (Figure S2.2).

$$f(\mathrm{s}) = \frac{(A_{\mathrm{H_A/H_{A'}}}/A_{\mathrm{CH_2}})_t}{(A_{\mathrm{H_A/H_{A'}}}/A_{\mathrm{CH_2}})_{t=0}} \quad \text{(Eqn. S14)}$$

$$f(\mathrm{s}) = \mathrm{e}^{-k_{ex}t} \quad \text{(Eqn. S15)}$$

The reaction data and observed first order rate constants for deuterium exchange (k_{ex}) are shown in Table S2.2 and Figures S2.3 – S2.4.

Figure S2.2: Plot of the fraction of remaining unexchanged α-protons H_A and $H_{A'}$ against time for the glycyl TKP (3.29 mM) in acetic acid buffer (0.025 M, 75 % fb) with 40 % d_3-MeCN co-solvent at pD = 6.28, I = 0.06 (KCl) and 25 ºC.

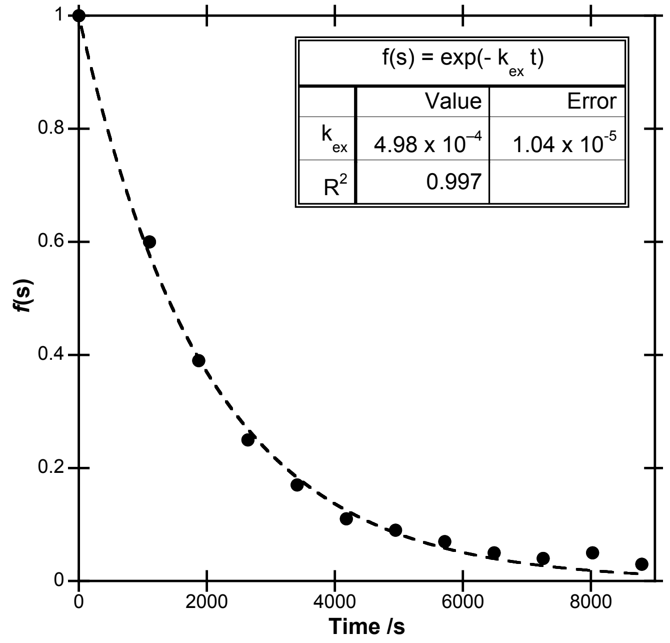

Figure S2.3: Plot of the fraction of unexchanged α-protons H_A and $H_{A'}$ against time for the glycyl TKP in 75 % fb 0.0250 M KOAc with 40 % d_3-MeCN co-solvent, I = 0.06 (KCl) and 25 ºC.

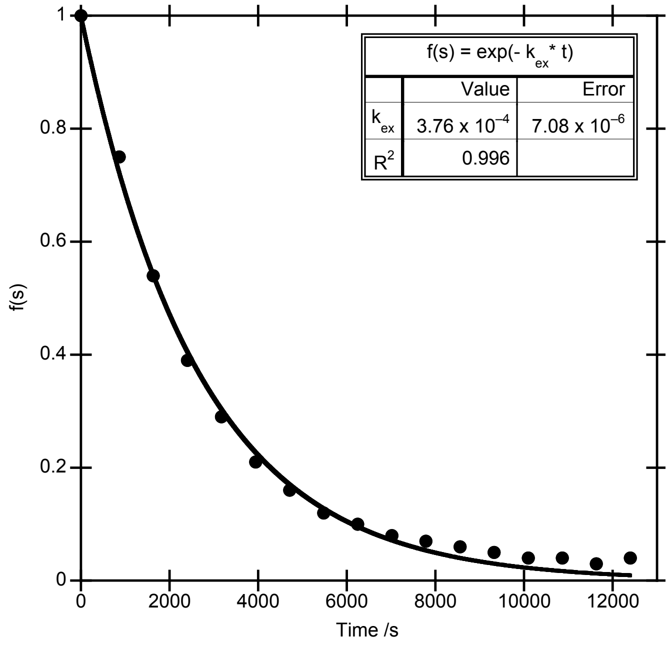

Figure S2.4: Plot of the fraction of unexchanged α-protons H_A and $H_{A'}$ against time for the glycyl TKP in 75 % fb 0.0500 M KOAc with 40 % d_3-MeCN co-solvent, I = 0.06 (KCl) and 25 ºC.

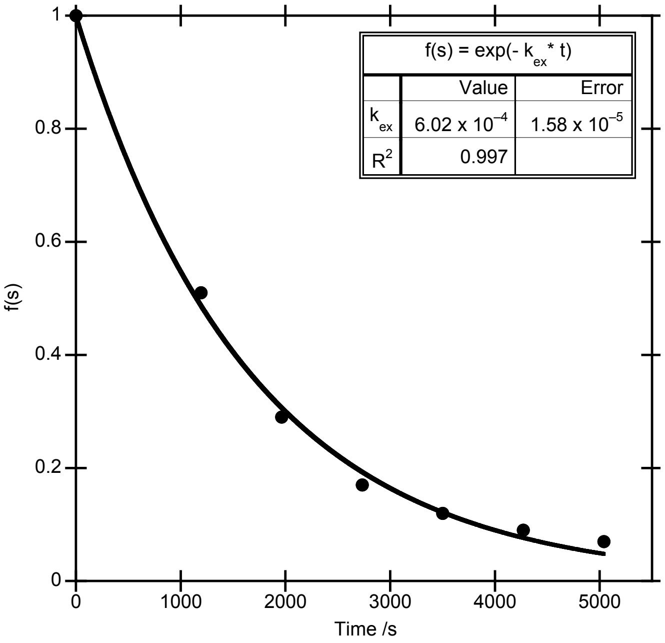

Table S2.2: Reaction data and observed first order rate constants (k_{ex}) for the deuterium exchange of the α-protons H_A and $H_{A'}$ in glycyl TKP (3.29 mM) in acetic acid buffer (0.0250 – 0.0500 M, 75 % fb) with 40 % d_3-MeCN co-solvent, I = 0.06 (KCl) and 25 ºC.

$[KOAc]_{Tot}$ /M	$[AcO^-]$ /M	$[DO^-]$ /M	Time /s	$f(s)^a$	k_{ex} /s^{-1}
0.0500	0.0375	3.03×10^{-10} (pD = 6.29)	0	1.00	6.02×10^{-4}
			1194	0.74	
			1963	0.54	
			2732	0.39	
			3501	0.33	
			4270	0.26	
			5039	0.22	
			5808	0.17	
			6577	0.14	
			7346	0.11	
			8115	0.09	
			8884	0.08	
			9653	0.08	
			10422	0.07	

			11191	0.05	
			11960	0.06	
			12729	0.06	
			13498	0.05	
			14267	0.03	
0.0375	0.0281	3.07×10^{-10} (pD = 6.29)	0	1.00	4.98×10^{-4}
			1104	0.60	
			1873	0.39	
			2642	0.25	
			3411	0.17	
			4180	0.11	
			4949	0.09	
			5718	0.07	
			6487	0.05	
			7256	0.04	
			8025	0.05	
			8794	0.03	
0.0250	0.0188	2.96×10^{-10} (pD = 6.28)	0	1.00	3.76×10^{-4}
			864	0.75	
			1633	0.54	
			2402	0.39	
			3171	0.29	
			3940	0.21	
			4709	0.16	
			5478	0.12	
			6247	0.1	
			7016	0.08	
			7785	0.07	
			8554	0.06	
			9323	0.05	
			10092	0.04	
			10861	0.04	
			11630	0.03	
			12399	0.04	

[a] fraction of unreacted substrate remaining at each time point.

The second order rate constant for the buffer catalysed exchange k_{A-} was determined from plotting k_{ex} against the concentration of the free base form of the buffer and fitting the

data to Eqn. S13 (Figure S2.5). From the plot a value of $k_{A-} = 1.21 \times 10^{-2}$ M^{-1} s^{-1} is found for the α-protons H_A and $H_{A'}$.

Figure S2.5: Plot of the dependence of k_{ex} upon the concentration of the free base form of the buffer for the glycyl TKP (3.29 mM) in acetic acid buffer (0.0250 – 0.0500 M, 75 % fb) with 40 % d_3-MeCN co-solvent, I = 0.06 (KCl) and 25 °C.

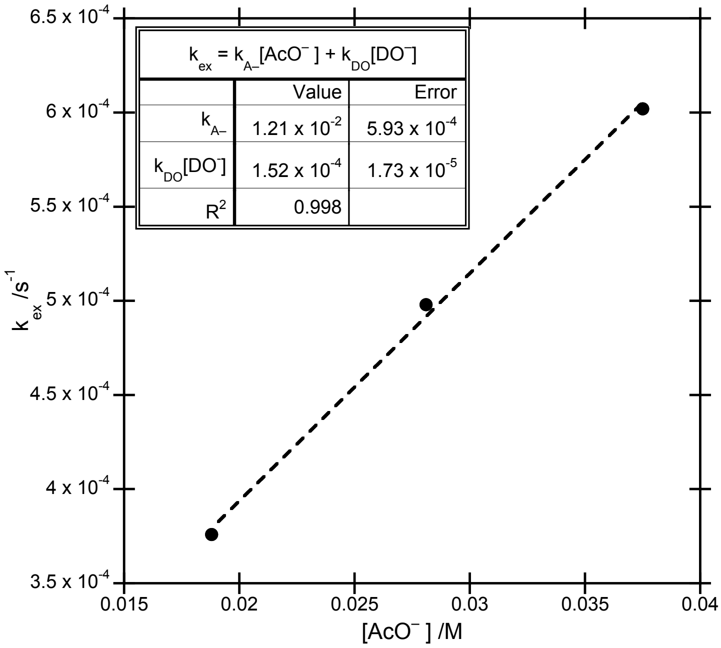

S2.4.6 Estimates for the second order rate constant for deuteroxide catalysed exchange, k_{DO}, for Glycyl TKP

The previously obtained k_{ex} values in Table S2.1 were used in conjunction with k_{A-} to obtain an estimate for the buffer independent first order rate constants for exchange, k_{ex}' (Eqn. S13 and Table S2.3).

An estimate for the second order rate constant for deuteroxide catalysed exchange, k_{DO}, was then found from plotting k_{ex}' against the concentration of deuteroxide (Figure S2.6) and linearly fitting the data according to Eqn. S13. The k_{DO} value for the α-protons H_A and $H_{A'}$ in the glycine TKP is estimated to be $k_{DO} = 5.09 \times 10^5$ M^{-1} s^{-1}. The linear fit crosses the y-axis at ~ 0 and therefore the extent of uncatalysed exchange by D_2O is negligible.

Table S2.3 k_{ex} values and contributions of buffer catalysis (k_{A-}[KOAc]) and deuteroxide catalysis (k_{ex}') to the deuterium exchange of the α-protons H_A and $H_{A'}$ of glycyl TKP (3.29 mM) in acetic acid buffer with 40 % d_3-MeCN co-solvent, I = 0.06 (KCl) and 25 ºC.

[KOAc] /M	[DO⁻] /M	k_{ex} /s^{-1}	k_{A-}[a][KOAc] /s^{-1} [b]	k_{ex}' / s^{-1} [c]	%Buffer catalysis	% DO⁻ catalysis
0.0375	3.03×10^{-10}	6.02×10^{-4}	4.54×10^{-4}	1.48×10^{-4}	75	25
0.0250	1.03×10^{-10}	3.69×10^{-4} [b]	3.03×10^{-4}	6.65×10^{-5}	82	18
0.0150	4.92×10^{-11}	2.04×10^{-4} [b]	1.82×10^{-4}	2.25×10^{-5}	89	11
0.0075	9.16×10^{-12}	8.50×10^{-5} [b]	9.08×10^{-5}	-5.75×10^{-6}	~100	~0
0.0025	2.64×10^{-12}	2.98×10^{-5} [b]	3.03×10^{-5}	-4.50×10^{-7}	~100	~0

[a] $k_{A-} = 1.21 \times 10^{-2}$ M^{-1} s^{-1}. [b] value obtained by multiplying k_{A-} by the concentration of potassium acetate. [c] $k_{ex}' = k_{ex} - k_{A-}$[KOAc].

Figure S2.6: Plot of the buffer independent first order rate constants of exchange (k_{ex}') against the concentration of deuteroxide for glycyl TKP (3.29 mM) in acetic acid buffer with 40 % d_3-MeCN co-solvent, I = 0.06 (KCl) and 25 ºC.

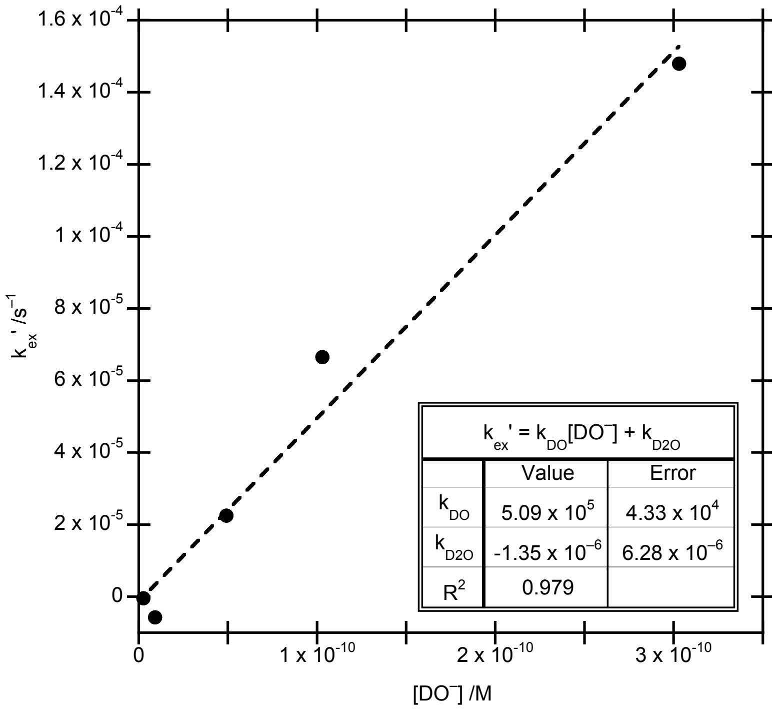

S2.4.7 First order rate constants for deuterium exchange, k_{ex}, for Prolyl TKP

In order to determine the kinetic acidity of the α-proton H_M in the prolyl TKP deuterium exchange reactions were carried out in acetic acid buffered D_2O solutions with 40 % d_3-MeCN co-solvent in the range p*D* 4.76 – 5.28. In order to delineate the contributions of deuteroxide ($k_{DO}[DO^-]$) and buffer ($k_{A-}[KOAc]$) components to the k_{ex} values (Eqn. S13), exchange was monitored in buffered solutions at four different free base percentages (15 %, 20 %, 25 % and 30 %), and four different concentrations of total buffer (0.20 M, 0.15 M, 0.125 M, 0.10 M) were used in each case. First order rate constants for deuterium exchange, k_{ex}, were determined by ^{1}H NMR spectroscopy and a representative set of spectra are shown in Figure S2.7. The α-proton H_M signal is at 4.93 ppm and decreases as exchange progresses.

Figure S2.7: Representative ^{1}H NMR spectra at 500 MHz for the hydrogen deuterium exchange of prolyl TKP (3.93 mM) in acetic acid buffer (0.2 M, 15 % fb, p*D* = 4.78) with 40 % d_3-MeCN co-solvent, I = 0.2 (KCl) and 25 ºC.

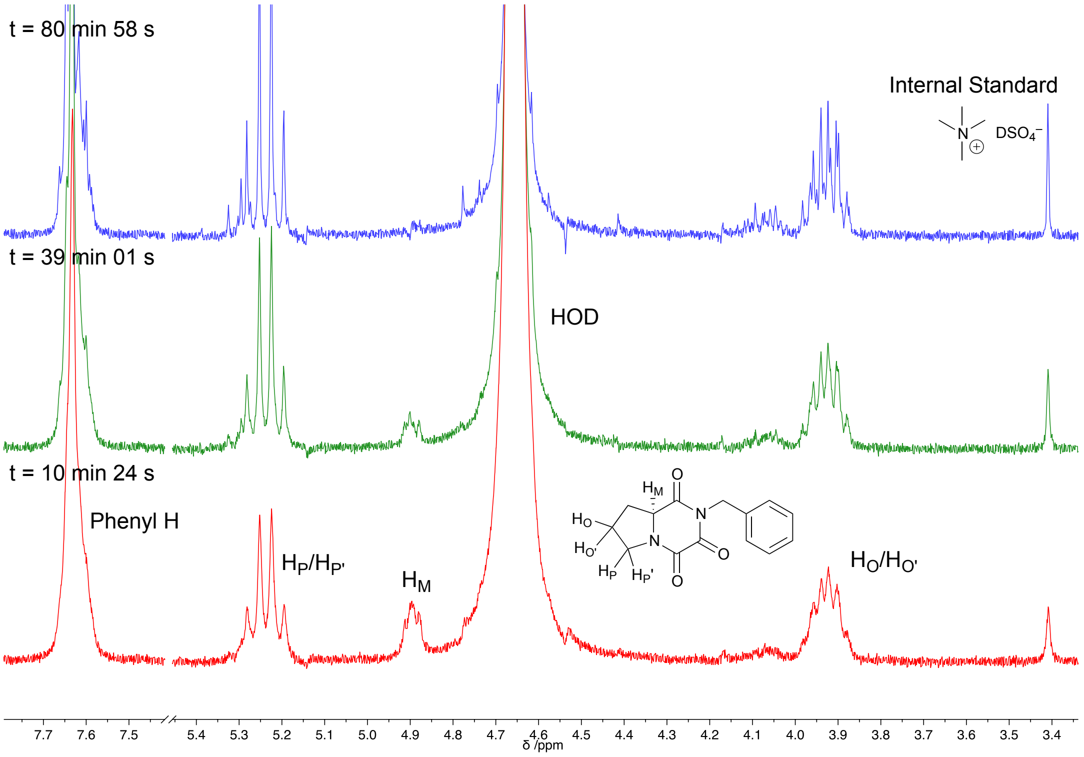

The integrated area of the H_M triplet decreased relative to the tetramethylammonium deuteriosulfate internal standard and the fraction of unexchanged substrate *f*(s) was determined

according to Eqn. S14.[viii] The observed first order rate constants for deuterium exchange, k_{ex}, were determined from fitting the reaction data to Equation S15 (Table S2.8). The reaction data and observed first order rate constants for deuterium exchange (k_{ex}) for all exchange reactions are shown in Figure S2.8 – Figure S2.23 and Table S2.4 – Table S2.7.

Table S2.4: Reaction data and observed first order rate constants (k_{ex}) for the deuterium exchange of the α-proton H_M in prolyl TKP in 15 % free base KOAc solutions with 40 % d_3-MeCN co-solvent, I = 0.2 (KCl) and 25 ºC.

[KOAc] /M	[fb KOAc] /M	[DO⁻] /M	Time /s	f(s)[a]	k_{ex} /s^{-1}
0.200	0.03 (15 % fb)	8.95×10^{-12} (pD 4.76)	624	0.75	4.06×10^{-4}
			1497	0.63	
			1923	0.53	
			2346	0.39	
			2763	0.29	
			3183	0.25	
			3598	0.24	
			4019	0.17	
			4442	0.13	
			4858	0.09	
0.150	0.023 (15 % fb)	9.05×10^{-12} (pD 4.76)	624	0.78	4.21×10^{-4}
			1061	0.65	
			1478	0.56	
			1895	0.43	
			2312	0.38	
			2728	0.36	
			3146	0.27	
			3566	0.22	
			3984	0.15	
			4399	0.13	
			4817	0.15	
			5233	0.08	
0.125	0.019 (15 % fb)	8.95×10^{-12} (pD 4.76)	504	0.84	4.18×10^{-4}
			937	0.62	
			1353	0.59	
			1767	0.44	

[viii] In Eqn. S14, A_{CH2} is replaced by A_{Int} for the area of the internal standard peak for prolyl TKP.

			2182	0.40	
			2602	0.31	
			3016	0.27	
			3437	0.24	
			3850	0.24	
			4266	0.16	
			4685	0.14	
			5105	0.17	
			5534	0.12	
			5982	0.10	
			624	0.79	
			1051	0.69	
			1467	0.64	
			1882	0.52	
			2379	0.43	
			2799	0.40	
			3219	0.33	
0.100	0.015 (15 % fb)	8.85×10^{-13} (p*D* 4.75)	3641	0.29	3.41×10^{-4}
			4055	0.22	
			4472	0.23	
			4887	0.20	
			5305	0.18	
			5725	0.14	
			6143	0.12	
			6560	0.10	

[a] fraction of unreacted substrate remaining at each time point.

Figure S2.8: Plot of the fraction of unexchanged α-proton H_M against time for the prolyl TKP in 15 % fb 0.200 M KOAc (p*D* 4.76) with 40 % d_3-MeCN co-solvent, *I* = 0.2 (KCl) and 25 ºC.

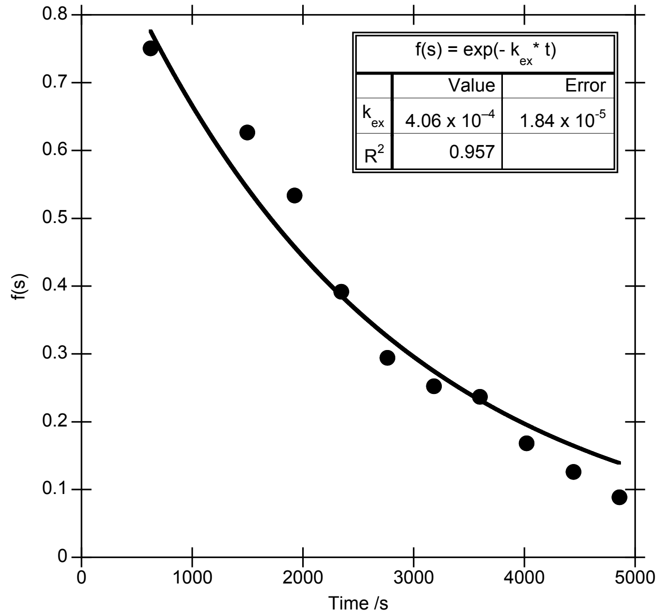

Figure S2.9: Plot of the fraction of unexchanged α-proton H_M against time for the prolyl TKP in 15 % fb 0.150 M KOAc (p*D* 4.76) with 40 % d_3-MeCN co-solvent, *I* = 0.2 (KCl) and 25 ºC.

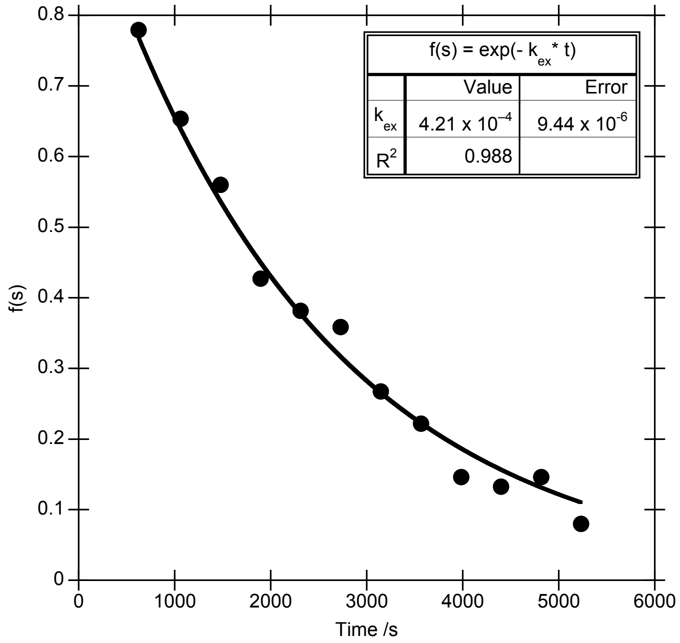

Figure S2.10: Plot of the fraction of unexchanged α-proton H_M against time for the prolyl TKP in 15 % fb 0.125 M KOAc (p*D* 4.76) with 40 % d_3-MeCN co-solvent, *I* = 0.2 (KCl) and 25 ºC.

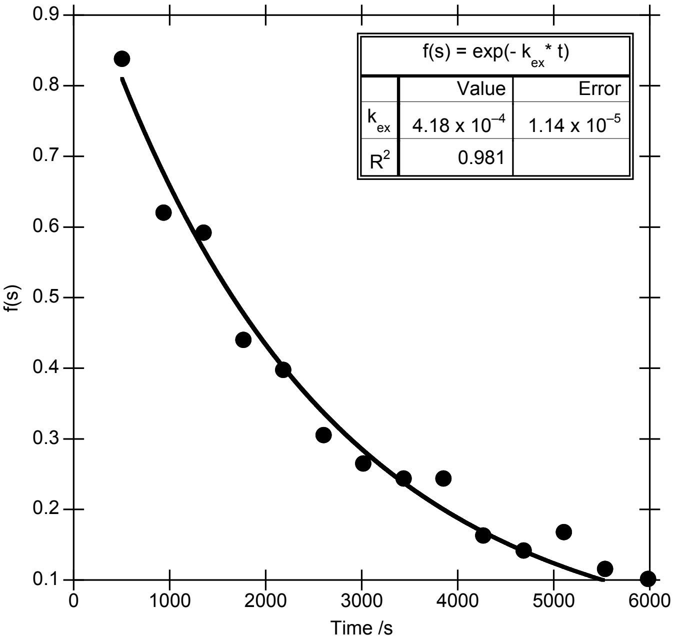

Figure S2.11: Plot of the fraction of unexchanged α-proton H_M against time for the prolyl TKP in 15 % fb 0.100 M KOAc (p*D* 4.75) with 40 % d_3-MeCN co-solvent, *I* = 0.2 (KCl) and 25 ºC.

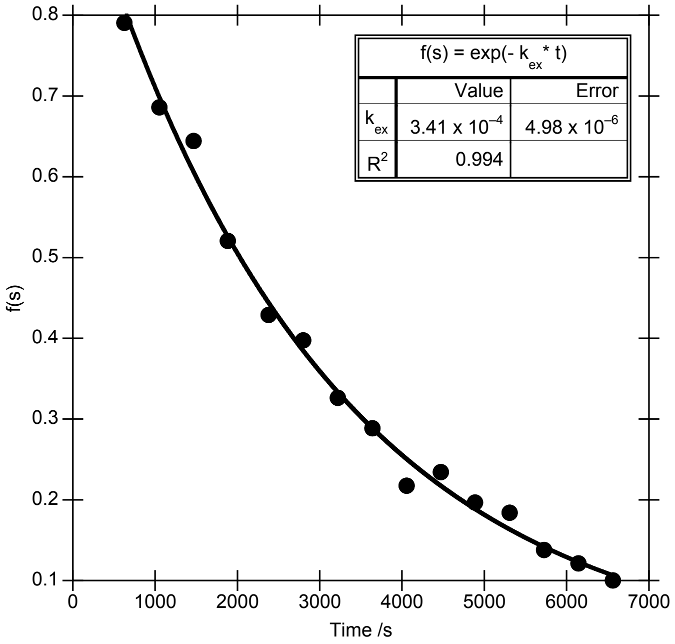

Table S2.5: Reaction data and observed first order rate constants (k_{ex}) for the deuterium exchange of the α-proton H_M in prolyl TKP in 20 % free base KOAc solutions with 40 % d_3-MeCN co-solvent, I = 0.2 (KCl) and 25 ºC.

[KOAc] /M	[fb KOAc] /M	[DO⁻] /M	Time /s	$f(s)^a$	k_{ex} /s^{-1}
			624	0.54	
			1048	0.40	
			1466	0.27	
0.200	0.04 (20 % fb)	1.50×10^{-11} (pD 4.98)	1882	0.19	8.93×10^{-4}
			2313	0.14	
			2736	0.08	
			3155	0.07	
			3577	0.06	
			624	0.67	
			1048	0.43	
			1466	0.30	
0.150	0.03 (20 % fb)	1.49×10^{-11} (pD 4.98)	1883	0.24	7.53×10^{-4}
			2303	0.17	
			2724	0.15	
			3145	0.11	
			3564	0.05	
			504	0.69	
			932	0.50	
			1352	0.40	
			1770	0.29	
0.125	0.025 (20 % fb)	1.47×10^{-11} (pD 4.97)	2186	0.24	6.96×10^{-4}
			2605	0.16	
			3021	0.11	
			3441	0.10	
			3863	0.07	
			4287	0.06	
			624	0.70	
			1045	0.60	
0.100	0.02 (20 % fb)	1.49×10^{-11} (pD 4.98)	1491	0.48	5.42×10^{-4}
			1914	0.35	
			2340	0.28	
			2755	0.22	

3174	0.16
3590	0.12
4013	0.10
4428	0.07
4852	0.08
5277	0.06
5698	0.06

[a] fraction of unreacted substrate remaining at each time point.

Figure S2.12: Plot of the fraction of unexchanged α-proton H_M against time for the prolyl TKP in 20 % fb 0.200 M KOAc (p*D* 4.98) with 40 % d_3-MeCN co-solvent, I = 0.2 (KCl) and 25 ºC.

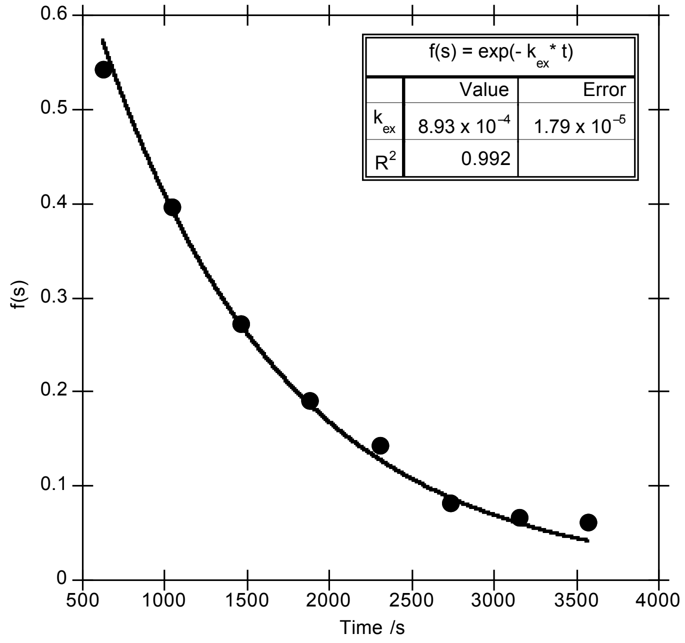

Figure S2.13: Plot of the fraction of unexchanged α-proton H_M against time for the prolyl TKP in 20 % fb 0.150 M KOAc (p*D* 4.98) with 40 % d_3-MeCN co-solvent, I = 0.2 (KCl) and 25 ºC.

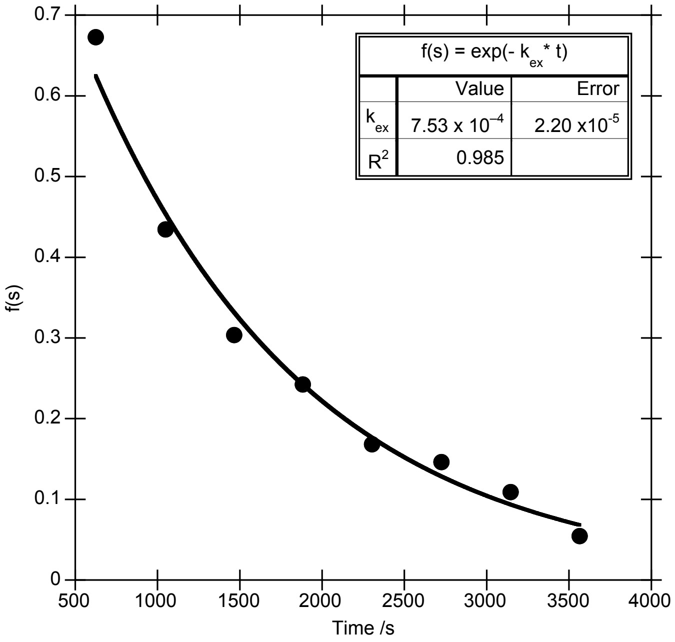

Figure S2.14: Plot of the fraction of unexchanged α-proton H_M against time for the prolyl TKP in 20 % fb 0.125 M KOAc (p*D* 4.97) with 40 % d_3-MeCN co-solvent, I = 0.2 (KCl) and 25 ºC.

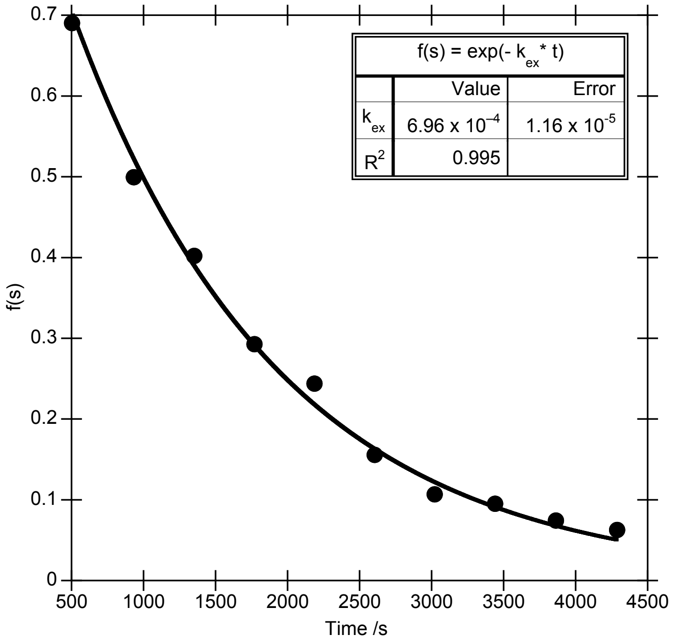

Figure S2.15: Plot of the fraction of unexchanged α-proton H_M against time for the prolyl TKP in 20 % fb 0.100 M KOAc (p*D* 4.98) with 40 % d_3-MeCN co-solvent, I = 0.2 (KCl) and 25 ºC.

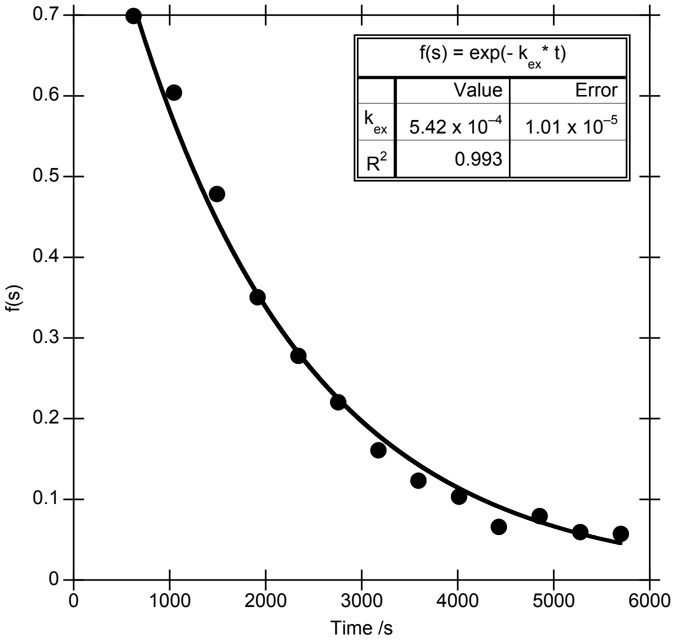

Table S2.6: Reaction data and observed first order rate constants (k_{ex}) for the deuterium exchange of the α-proton H_M in prolyl TKP in 25 % free base KOAc solutions with 40 % d_3-MeCN co-solvent, I = 0.2 (KCl) and 25 ºC.

[KOAc] /M	[fb KOAc] /M	[DO$^-$] /M	Time /s	f(s)[a]	k_{ex} /s^{-1}
			624	0.56	
			1041	0.39	
0.200	0.05 (25 % fb)	2.25×10^{-11} (p*D* 5.16)	1464	0.21	9.73×10^{-4}
			1881	0.15	
			2337	0.10	
			2752	0.06	
			624	0.60	
			1036	0.38	
			1456	0.24	
0.150	0.038 (25 % fb)	2.17×10^{-11} (p*D* 5.14)	1877	0.16	9.22×10^{-4}
			2296	0.12	
			2717	0.07	
			3135	0.06	
			3552	0.05	
0.125	0.031	2.15×10^{-11}	624	0.62	8.12×10^{-4}

	(25 % fb)	(p*D* 5.14)	1044	0.43	
			1462	0.31	
			1878	0.20	
			2294	0.14	
			2711	0.12	
			3126	0.09	
			3550	0.06	
0.10	0.025 (25 % fb)	2.15×10^{-11} (p*D* 5.14)	624	0.67	7.22×10^{-4}
			1042	0.46	
			1457	0.34	
			1875	0.26	
			2331	0.17	
			2755	0.15	
			3172	0.09	
			3592	0.06	
			4009	0.05	

[a] fraction of unreacted substrate remaining at each time point.

Figure S2.16: Plot of the fraction of unexchanged α-proton H_M against time for the prolyl TKP in 25 % fb 0.200 M KOAc (p*D* 5.16) with 40 % d_3-MeCN co-solvent, *I* = 0.2 (KCl) and 25 ºC.

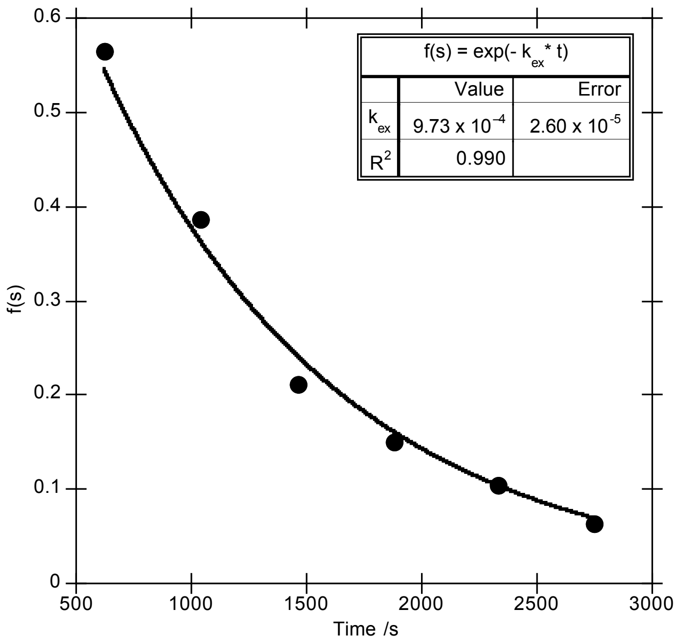

Figure S2.17: Plot of the fraction of unexchanged α-proton H_M against time for the prolyl TKP in 25 % fb 0.150 M KOAc (p*D* 5.14) with 40 % d_3-MeCN co-solvent, I = 0.2 (KCl) and 25 ºC.

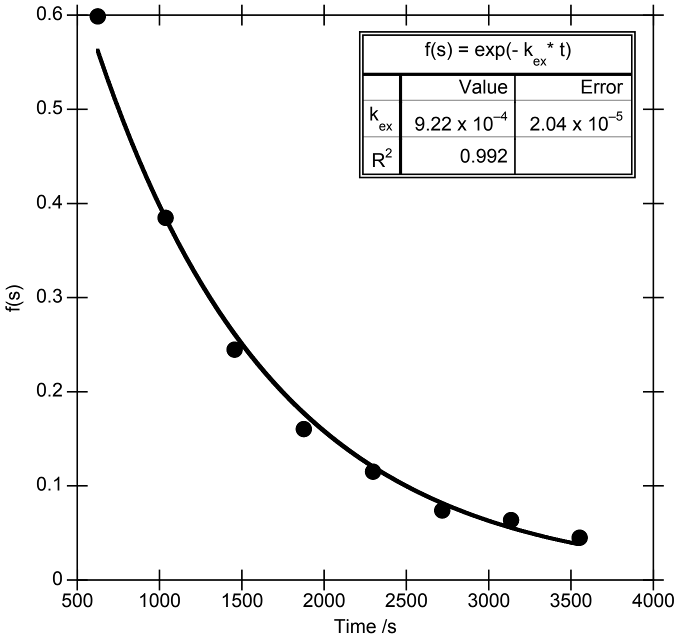

Figure S2.18: Plot of the fraction of unexchanged α-proton H_M against time for the prolyl TKP in 25 % fb 0.125 M KOAc (p*D* 5.14) with 40 % d_3-MeCN co-solvent, I = 0.2 (KCl) and 25 ºC.

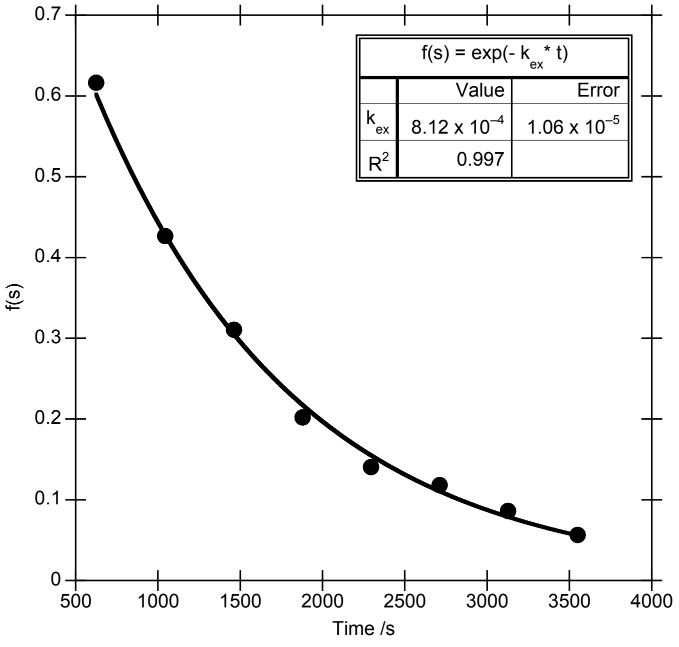

Figure S2.19: Plot of the fraction of unexchanged α-proton H_M against time for the prolyl TKP in 25 % fb 0.100 M KOAc (p*D* 5.14) with 40 % d_3-MeCN co-solvent, I = 0.2 (KCl) and 25 ºC.

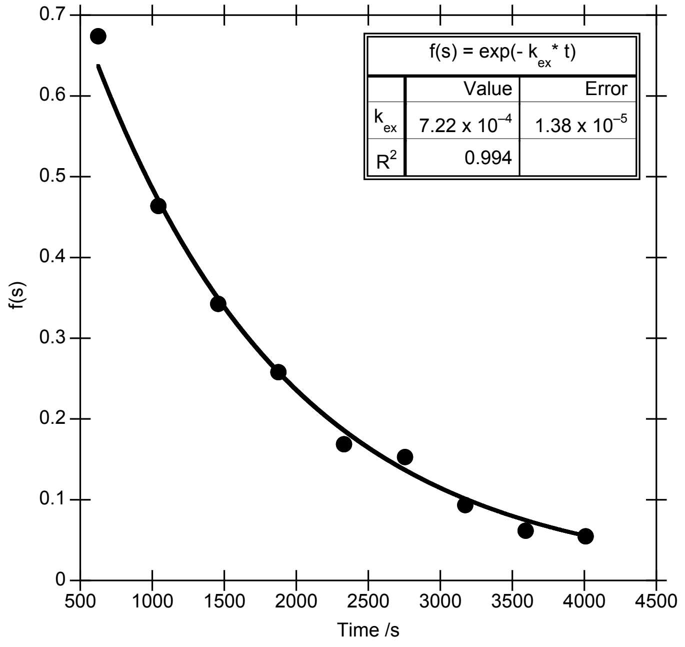

Table S2.7: Reaction data and observed first order rate constants (k_{ex}) for the deuterium exchange of the α-proton H_M in prolyl TKP in 30 % free base KOAc solutions with 40 % d_3-MeCN co-solvent, $I = 0.2$ (KCl) and 25 ºC.

[KOAc] /M	[fb KOAc] /M	[DO⁻] /M	Time /s	f(s)[a]	k_{ex} /s^{-1}
0.200	0.06 (30 % fb)	3.03×10^{-11} (pD 5.29)	744	0.38	1.34×10^{-3}
			1548	0.10	
			2352	0.07	
			3161	0.00	
0.150	0.045 (30 % fb)	3.00×10^{-11} (pD 5.28)	564	0.53	1.14×10^{-3}
			981	0.31	
			1410	0.18	
			1839	0.14	
			2264	0.09	
			2714	0.07	
0.125	0.038 (30 % fb)	2.93×10^{-11} (pD 5.27)	624	0.58	9.68×10^{-4}
			1084	0.34	
			1543	0.20	
			1965	0.14	
			2385	0.10	
			2806	0.05	
			3227	0.05	
			3971	0.03	
0.100	0.03 (30 % fb)	2.90×10^{-11} (pD 5.27)	564	0.61	8.67×10^{-4}
			984	0.42	
			1401	0.29	
			1819	0.21	
			2242	0.14	
			2673	0.11	
			3093	0.07	
			3510	0.05	
			3939	0.04	
			4378	0.02	

[a] fraction of unreacted substrate remaining at each time point.

Figure S2.20: Plot of the fraction of unexchanged α-proton H_M against time for the prolyl TKP in 30 % fb 0.200 M KOAc (p*D* 5.29) with 40 % d_3-MeCN co-solvent, *I* = 0.2 (KCl) and 25 ºC.

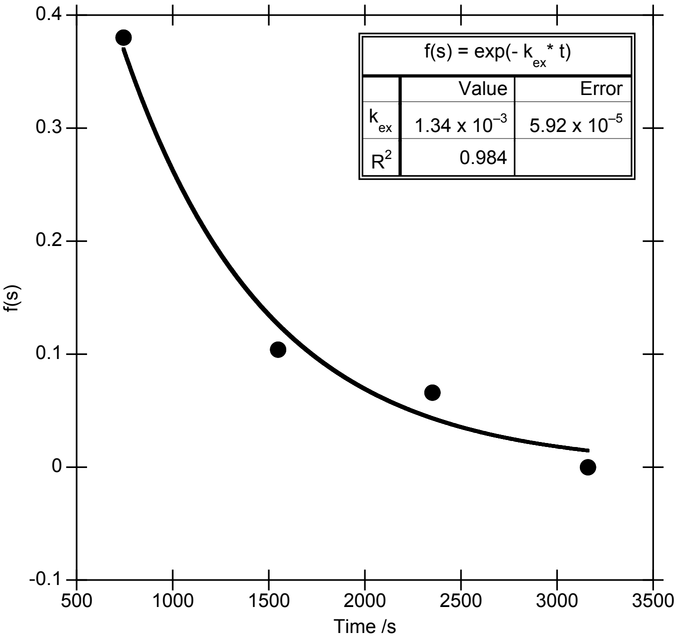

Figure S2.21: Plot of the fraction of unexchanged α-proton H_M against time for the prolyl TKP in 30 % fb 0.150 M KOAc (p*D* 5.28) with 40 % d_3-MeCN co-solvent, *I* = 0.2 (KCl) and 25 ºC.

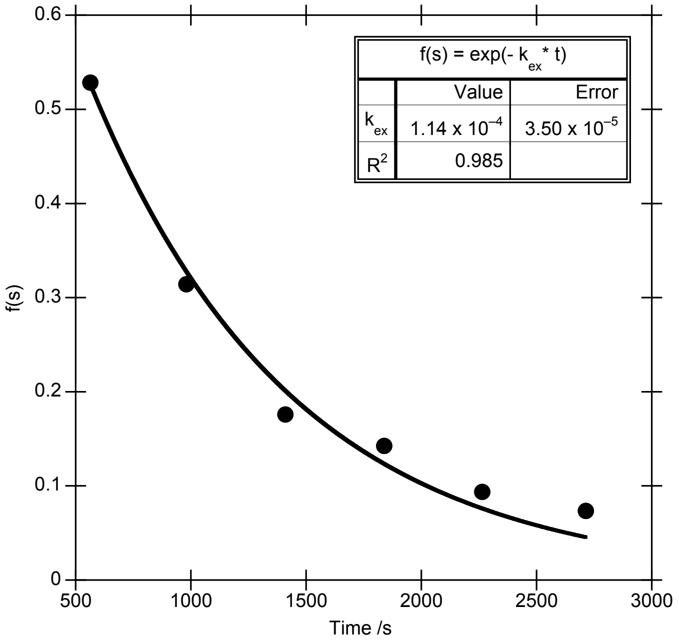

Figure S2.22: Plot of the fraction of unexchanged α-proton H_M against time for the prolyl TKP in 30 % fb 0.125 M KOAc (p*D* 5.27) with 40 % d_3-MeCN co-solvent, *I* = 0.2 (KCl) and 25 ºC.

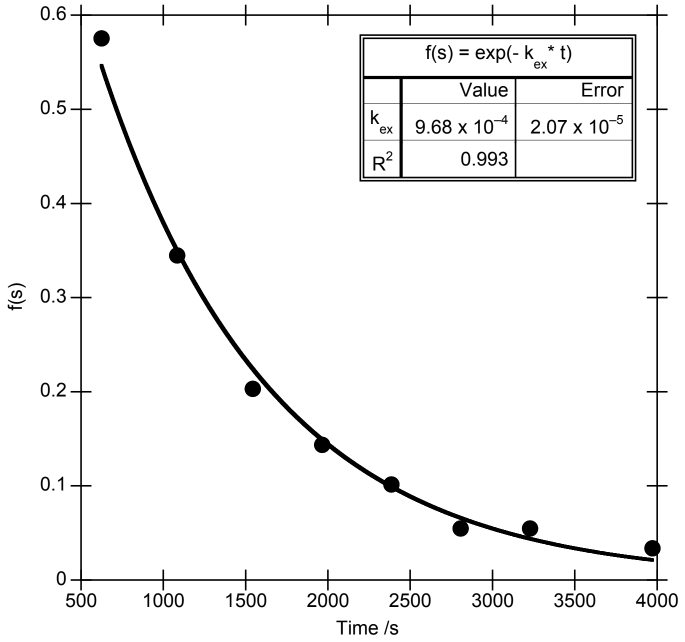

Figure S2.23: Plot of the fraction of unexchanged α-proton H_M against time for the prolyl TKP in 30 % fb 0.100 M KOAc (p*D* 5.27) with 40 % d_3-MeCN co-solvent, *I* = 0.2 (KCl) and 25 ºC.

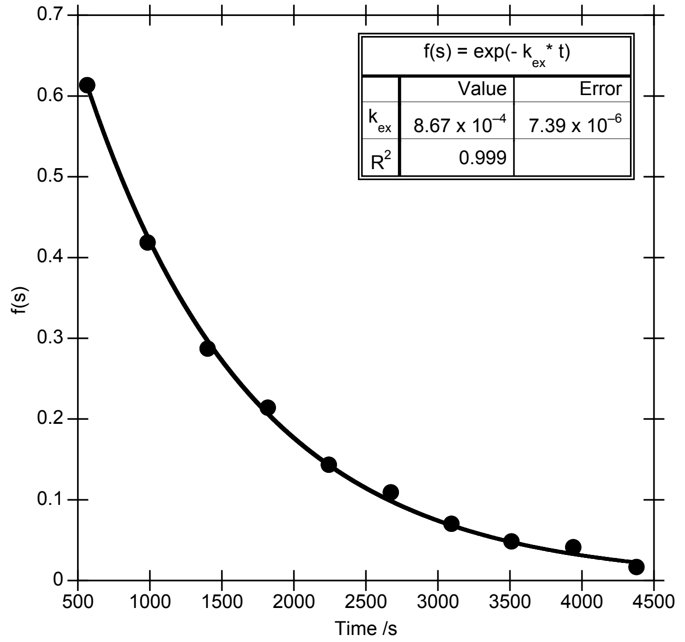

S2.4.8 Second order rate constants for buffer catalysed, k_{A-}, and deuteroxide catalysed, k_{DO}, exchange for Prolyl TKP

Buffer catalysis of deuterium exchange is clearly observed for prolyl TKP under all conditions. The second order rate constants for buffer catalysed exchange were determined using Eqn. S13 and plotting the k_{ex} against the concentration of the free base form of the buffer Figure S2.24 and Table S2.25. Figure S2.25 is identical to Figure S2.24 but with two anomalous data points (15 % fb 0.20 M KOAc, and, 25 % fb 0.20 M KOAc) removed.
The value for k_{A-} should not change between the different percentage free base solutions.[ix] Gratifyingly, for experiments in 20 % and 30 % free base, the values for k_{A-} are closely similar at 1.66×10^{-2} M^{-1} s^{-1} and 1.62×10^{-2} M^{-1} s^{-1} respectively (Figure S2.24). The k_{A-} values for 15 % and 25 % free base in Figure S2.24 were below this k_{A-} value ($k_{A-} = 3.43 \times 10^{-3}$ M^{-1} s^{-1} and $k_{A-} = 1.00 \times 10^{-2}$ M^{-1} s^{-1}, respectively). For 25 % free base it was found that omitting the k_{ex} value for 0.20 M 25 % fb KOAc (Figure S2.25) led to a rise in k_{A-} to 1.54×10^{-2} M^{-1} s^{-1} which is consistent with the k_{A-} values for 20 % and 30 % free base above. For 15 % free base omitting the k_{ex} value for 0.20 M 15 % fb KOAc gave $k_{A-} = 1.00 \times 10^{-2}$ M^{-1} s^{-1} which still remains below the k_{A-} values for 20 %, 25 % and 30 % free base above (Figure S2.25).
The origin of the inconsistent k_{A-} value for 15 % free base is presumed to be due to the poor fitting of the data for the 15 % free base. Owing to the limited amounts of material available the experiment could not be repeated.

[ix] Assuming only general base catalysis was active and no general acid catalysis was present.

Figure S2.24: Plot of k_{ex} of the α-proton H_M in prolyl TKP (3.93 mM) against free base buffer concentration in a series of acetic acid buffer solutions with 40 % d_3-MeCN co-solvent, I = 0.2 (KCl) and 25 °C.

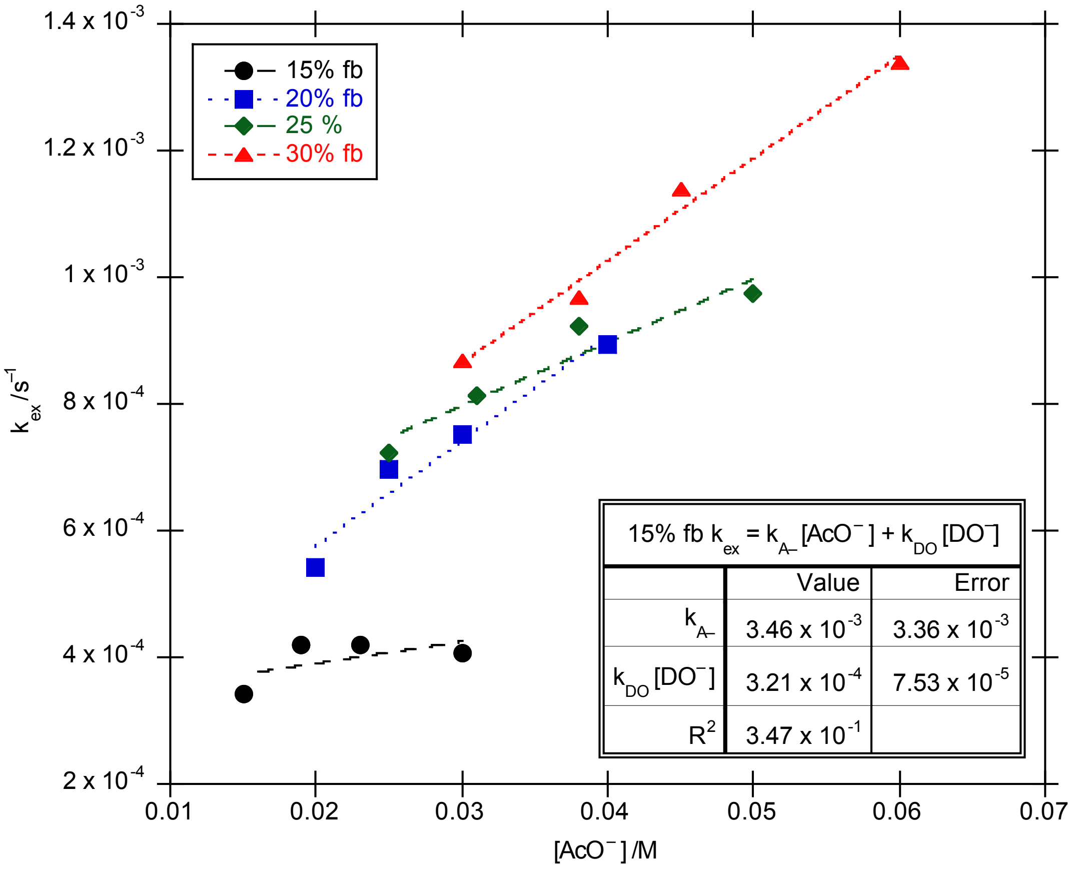

15% fb $k_{ex} = k_{A-}[AcO^-] + k_{DO}[DO^-]$	Value	Error
k_{A-}	3.46 x 10^{-3}	3.36 x 10^{-3}
$k_{DO}[DO^-]$	3.21 x 10^{-4}	7.53 x 10^{-5}
R^2	3.47 x 10^{-1}	

20% fb $k_{ex} = k_{A-}[AcO^-] + k_{DO}[DO^-]$	Value	Error
k_{A-}	1.66 x 10^{-2}	2.56 x 10^{-3}
$k_{DO}[DO^-]$	2.43 x 10^{-4}	7.6 x 10^{-5}
R^2	0.955	

25% fb $k_{ex} = k_{A-}[AcO^-] + k_{DO}[DO^-]$	Value	Error
k_{A-}	1.00 x 10^{-2}	2.17 x 10^{-3}
$k_{DO}[DO^-]$	4.97 x 10^{-4}	8.06 x 10^{-5}
R^2	0.914	

30% fb $k_{ex} = k_{A-}[AcO^-] + k_{DO}[DO^-]$	Value	Error
k_{A-}	1.62 x 10^{-2}	1.38 x 10^{-3}
$k_{DO}[DO^-]$	3.79 x 10^{-4}	6.16 x 10^{-5}
R^2	0.986	

Figure S2.25: Plot of k_{ex} of the α-proton H_M in prolyl TKP (3.93 mM) against free base buffer concentration in a series of acetic acid buffer solutions with 40 % d_3-MeCN co-solvent, I = 0.2 (KCl) and 25 ºC. The value for 15% fb the [KOAc] = 0.2 M and 25% fb the [KOAc] = 0.2 M have been omitted from this plot. Full data is in Figure S2.7.

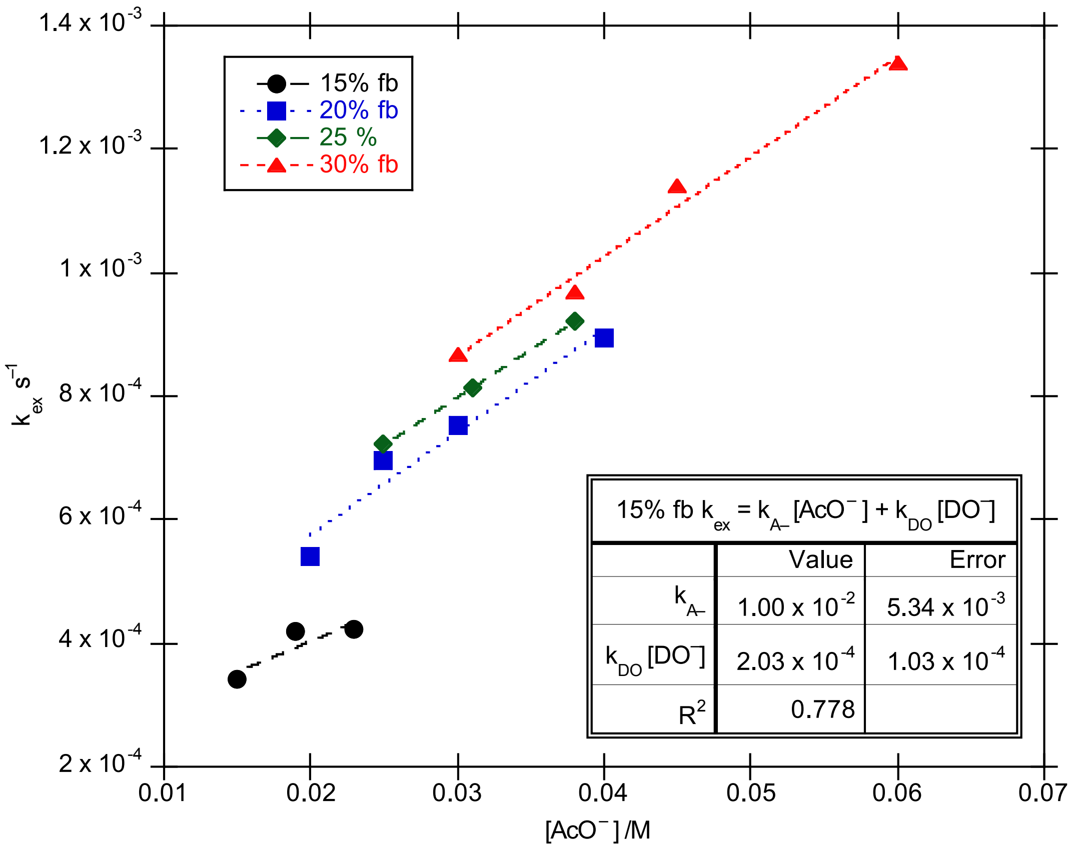

20% fb $k_{ex} = k_{A-}[AcO^-] + k_{DO}[DO^-]$		
	Value	Error
k_{A-}	1.66×10^{-2}	2.56×10^{-3}
$k_{DO}[DO^-]$	2.43×10^{-4}	7.6×10^{-5}
R^2	0.955	

25% fb $k_{ex} = k_{A-}[AcO^-] + k_{DO}[DO^-]$		
	Value	Error
k_{A-}	1.54×10^{-2}	2.05×10^{-4}
$k_{DO}[DO^-]$	3.36×10^{-4}	6.5×10^{-6}
R^2	1.00	

30% fb $k_{ex} = k_{A-}[AcO^-] + k_{DO}[DO^-]$		
	Value	Error
k_{A-}	1.62×10^{-2}	1.38×10^{-3}
$k_{DO}[DO^-]$	3.79×10^{-4}	6.16×10^{-5}
R^2	0.986	

Table S2.8: k_{ex}, k_{buff} and k_{ex}' values for the deuterium exchange of the α-proton H_M in 3.93 mM prolyl TKP in a set of different percentage free base acetic acid buffer solutions with 40 % d_3-MeCN co-solvent, I = 0.2 (KCl) and 25 ºC.

% fb	[KOAc] /M	[AcO⁻] /M	[DO⁻] /M	k_{ex} /s⁻¹	k_{A-} /M⁻¹ s⁻¹	k_{ex}' /s⁻¹
15	0.200	0.030	8.95×10^{-12}	4.06×10^{-4} [a]	1.00×10^{-2} [a] (3.46×10^{-3}) [b]	2.03×10^{-4}
	0.150	0.023	9.05×10^{-12}	4.21×10^{-4}		
	0.125	0.019	8.95×10^{-12}	4.18×10^{-4}		
	0.100	0.015	8.85×10^{-13}	3.41×10^{-4}		
20	0.200	0.040	1.50×10^{-11}	8.93×10^{-4}	1.66×10^{-2}	2.43×10^{-4}
	0.150	0.030	1.49×10^{-11}	7.53×10^{-4}		
	0.125	0.025	1.47×10^{-11}	6.96×10^{-4}		
	0.100	0.020	1.49×10^{-11}	5.42×10^{-4}		
25	0.200	0.050	2.25×10^{-11}	9.73×10^{-4} [a]	1.54×10^{-2} [a] (1.00×10^{-2}) [b]	3.36×10^{-4}
	0.150	0.038	2.17×10^{-11}	9.22×10^{-4}		
	0.125	0.031	2.15×10^{-11}	8.12×10^{-4}		
	0.100	0.025	2.15×10^{-11}	7.22×10^{-4}		
30	0.200	0.060	3.03×10^{-11}	1.34×10^{-3}	1.62×10^{-2}	3.79×10^{-4}
	0.150	0.045	3.00×10^{-11}	1.14×10^{-3}		
	0.125	0.038	2.93×10^{-11}	9.68×10^{-4}		
	0.100	0.030	2.90×10^{-11}	8.67×10^{-4}		

[a] Anomalous results omitted during fitting in Figure S2.8. [b] Value for k_{A-} without anomalous results removed.

From the y-intercepts in Figure S2.26 buffer independent first order rate constants for exchange, k_{ex}' (s⁻¹), were determined. The gradient of a plot of k_{ex}' against the concentration of deuteroxide gave the second order rate constant for deuteroxide catalysed exchange for the α-proton H_M in the proline TKP of $k_{DO} = 8.99 \times 10^6$ M⁻¹ s⁻¹.

Figure S2.26: Plot of the buffer independent first order rate constants of exchange (k_{ex}') against the concentration of deuteroxide for prolyl TKP (3.93 mM) in acetic acid buffer solutions with 40 % d_3-MeCN co-solvent, I = 0.2 (KCl) and 25 ºC.

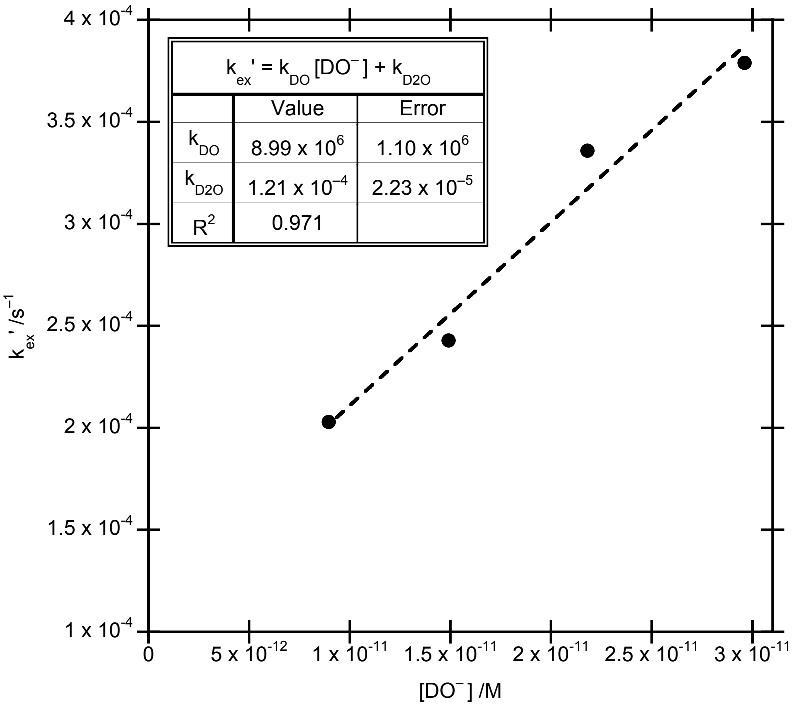

S2.4.9 Converting the experimental k_{DO} values to k_{HO} values in Table 3 of the Main Text

The k_{DO} values above are for a 60 : 40 D_2O : d_3-MeCN solution. In order to make comparisons to the other carbon acids whose k_{DO} values were recorded in 100 % D_2O it is necessary to adjust the k_{DO} values of the TKPs to account for this. A previous study by O'Donoghue on the carbon acidity of triazolium ions showed that there was between a 1.2 – 5.4-fold increase in k_{DO} upon moving from 100 % D_2O to a 2 : 1 D_2O : d_3-MeCN solution.[7] Deuteroxide is a stronger base in a mixed solvent than it is in pure D_2O as the poorer hydrogen bonding ability of the mixed solvent destabilises the deuteroxide. Unfortunately, due to solubility, it was not possible to determine a k_{DO} value in 100 % D_2O for the TKPs. To overcome this we have used the 5.4-fold difference in k_{DO} determined by O'Donoghue to convert our k_{DO} values for 60 : 40 D_2O : d_3-MeCN into k_{DO} values for 100 % D_2O. This gives $k_{DO} = 9.43 \times 10^4$ M^{-1} s^{-1} for glycyl TKP **10** and 1.66×10^6 M^{-1} s^{-1} for prolyl TKP **11**. A secondary solvent isotope effect of $k_{DO}/k_{HO} = 1.46$ was then used to convert k_{DO} to k_{HO} for the TKPs in Table 3 of the Main Text.

S3 Computational Results

S3.1 Computational Methods

Calculations were performed using either Spartan 10 (Windows) or Q-Chem 4.0 (Windows) at the BMK/6-31+G(d) level of theory. Zero point vibrational energies were calculated for all geometry optimised structures, and the absence of imaginary frequencies was used to characterise them as minima on their potential energy surfaces. NBO calculations were performed using the NBO keyword within Q-Chem 4.0 (Windows).

S3.2 Geometry Optimisations using BMK/6-31+G(d)

S3.2.1 Gly-L-Pro

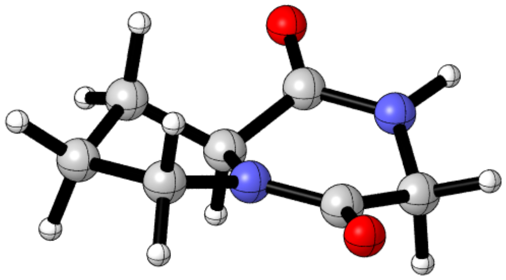

H	-2.130988	-0.993553	1.413890
C	-1.951230	-0.889783	0.330235
C	-0.985954	1.372019	-0.038095
N	0.448006	-0.659627	0.085625
C	0.312749	0.745013	0.482150
C	-0.586948	-1.521496	0.005653
N	-2.025162	0.499421	-0.097572
H	0.284381	0.831485	1.584458
H	-2.713779	-1.475566	-0.189848
H	-2.911141	0.880280	-0.406419
C	1.604602	1.384617	-0.055010
H	1.469417	1.628131	-1.116641
H	1.863811	2.302254	0.480402
C	1.825387	-1.026834	-0.252871
H	1.897197	-1.248071	-1.327213
H	2.125798	-1.923598	0.300613
C	2.625872	0.238442	0.133220
H	3.526502	0.360412	-0.477755
H	2.929288	0.177961	1.187455
O	-1.070821	2.548924	-0.341032
O	-0.475086	-2.702694	-0.283592

E = -532.469614 au (BMK/6-31+G(d)//BMK/6-31+G(d))

This Molecule has 0 Imaginary Frequencies
Zero point vibrational energy: 110.994 kcal/mol

S3.2.2 Gly-L-Pro (Pro enolate)

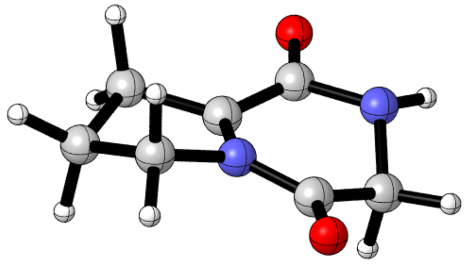

N	2.053442	-0.049110	0.278407
C	-0.097245	0.834302	-0.155334
C	0.189506	-1.610398	-0.033358
N	-0.569596	-0.519008	0.065691
C	1.629378	-1.253783	-0.426376
C	1.245379	1.124309	0.077358
C	-1.992755	-0.536010	0.350564
H	-2.163559	-0.534111	1.440631
H	-2.453179	-1.439072	-0.066263
O	-0.204474	-2.779320	0.130585
O	1.804962	2.249515	0.141070
H	3.025908	0.193764	0.119211
H	1.645138	-1.120399	-1.532139
H	2.270328	-2.100177	-0.159179
C	-1.289526	1.759166	-0.073852
H	-1.277300	2.556734	-0.829968
H	-1.381153	2.263549	0.909806
C	-2.482554	0.786760	-0.284362
H	-2.650670	0.628068	-1.358863
H	-3.419434	1.140841	0.167011

E = -531.876653 au (BMK/6-31+G(d)//BMK/6-31+G(d))
This Molecule has 0 Imaginary Frequencies
Zero point vibrational energy: 101.143 kcal/mol

S3.2.3 Gly-L-Pro (Gly enolate)

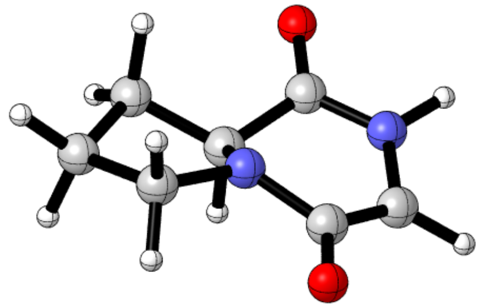

C	-1.436373	-1.550345	0.207036
C	-1.456954	0.902036	0.004785
N	0.567169	-0.412612	-0.260790
C	0.000168	0.734221	0.437586
C	-0.058023	-1.676675	-0.014776
N	-2.078439	-0.285481	-0.017714
H	-0.003316	0.563267	1.542215
H	-2.071740	-2.398290	0.426062
H	-3.039125	-0.266745	-0.338553
C	0.960165	1.871036	0.076901
H	0.741513	2.206631	-0.945444
H	0.882069	2.734233	0.748111
C	2.021640	-0.306567	-0.331138
H	2.365025	-0.475275	-1.363582
H	2.497598	-1.070342	0.300634
C	2.324149	1.141247	0.155516
H	3.112360	1.627276	-0.434658
H	2.657739	1.113575	1.203628
O	-1.982905	1.988610	-0.289303
O	0.646422	-2.718285	-0.011240

E = -531.872423 au (BMK/6-31+G(d)//BMK/6-31+G(d))
This Molecule has 0 Imaginary Frequencies
Zero point vibrational energy: 101.089 kcal/mol

S3.2.4 2,3,6-Trihydroxypyrazine

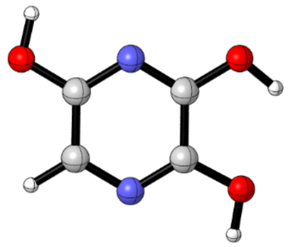

C	-0.612787	-0.792898	0.000000
C	-1.070843	0.549016	0.000000
N	0.678280	-1.055038	0.000000
C	1.520170	-0.020054	0.000000
C	1.076050	1.306361	0.000000
H	1.764598	2.145682	0.000000
N	-0.239737	1.561784	0.000000
O	-1.463296	-1.823352	0.000000
H	-2.374382	-1.492036	0.000000
O	-2.412124	0.740121	0.000000
H	-2.582780	1.693951	0.000000
O	2.837442	-0.280067	0.000000
H	2.951041	-1.242982	0.000000

E = -489.777261 au (BMK/6-31+G(d)//BMK/6-31+G(d))
This Molecule has 0 Imaginary Frequencies
Zero point vibrational energy: 57.871 kcal/mol

S3.2.5 TKP enolate

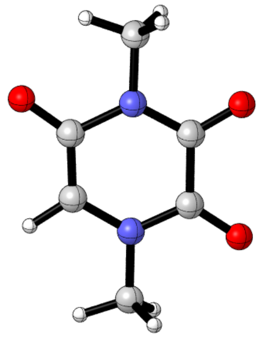

C	-0.259717	-1.176734	0.000019
O	-0.539893	-2.383675	-0.000059
C	1.173267	-0.706882	0.000062
O	2.137926	-1.501047	-0.000015
N	1.347594	0.644884	0.000102
N	-1.243277	-0.208760	0.000026
C	-1.026453	1.230627	-0.000144
C	0.308821	1.600256	-0.000044
H	0.599014	2.639572	-0.000341
O	-2.034300	1.992045	-0.000266
C	-2.620823	-0.688635	-0.000007
H	-2.804161	-1.310430	0.882874
H	-2.804452	-1.309612	-0.883415
H	-3.263859	0.190843	0.000474
C	2.728943	1.108736	-0.000010
H	3.257059	0.729530	0.881105
H	2.735586	2.199923	0.001468
H	3.256178	0.731969	-0.882735

E = -567.819520 au (BMK/6-31+G(d)//BMK/6-31+G(d))
This Molecule has 0 Imaginary Frequencies
Zero point vibrational energy: 85.143 kcal/mol

S3.2.6 c(D-Pro-D-Pro) + LiOH

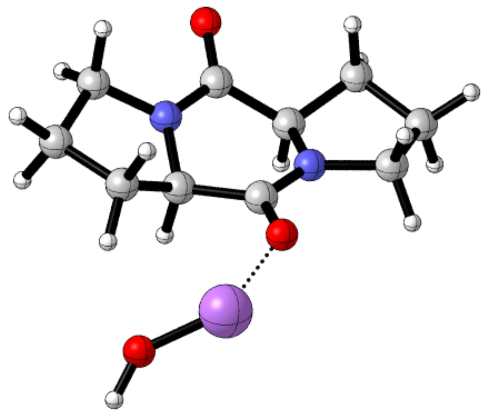

C	1.791983	-0.216599	0.589395
C	-0.254272	1.028534	-0.072169
N	-0.245552	-1.447985	0.166703
C	-1.024737	-0.212011	0.365712
C	1.105286	-1.529758	0.205317
N	1.074439	0.957309	0.051625
H	1.811209	-0.152066	1.689368
H	-1.259293	-0.077233	1.435741
O	1.742962	-2.550484	-0.029105
C	3.206955	-0.036891	0.029832
H	3.253081	-0.459443	-0.979312
H	3.954229	-0.551083	0.637384
C	1.988227	2.031162	-0.378318
H	1.728038	2.971542	0.116078
H	1.879497	2.182599	-1.458286
C	3.379380	1.493724	0.007078
H	3.659208	1.857801	1.002393
H	4.151993	1.822828	-0.693045
C	-2.328529	-0.486593	-0.403998
H	-3.160989	0.127975	-0.033174
H	-2.172641	-0.259277	-1.466733
C	-1.087113	-2.575566	-0.267375
H	-0.929564	-3.439900	0.384932
H	-0.798403	-2.872662	-1.282858
C	-2.513104	-2.000644	-0.202029
H	-2.953609	-2.195261	0.782876
H	-3.172145	-2.447471	-0.951225
O	-0.822562	2.054554	-0.498786
Li	-2.604151	2.556010	-0.187637
O	-4.014982	1.993082	0.487653
H	-4.808164	2.308994	0.931693

E = -732.5408966 au (BMK/6-31+G(d) (H_2O)//B3LYP/6-31G(d))
This Molecule has 0 Imaginary Frequencies
Zero point vibrational energy: 159.33 kcal/mol

S3.2.7 c(D-Pro-L-Pro) + LiOH

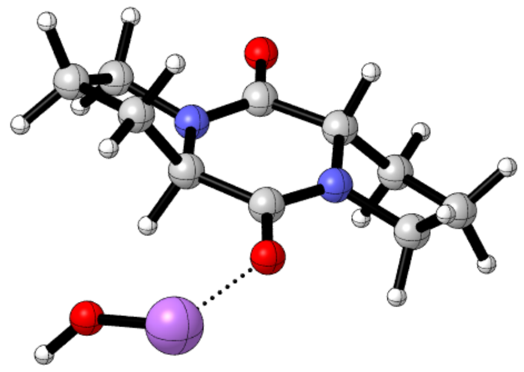

N	-0.683987	-1.194813	0.270884
C	-0.005196	1.136662	-0.392934
C	1.664035	-0.716030	-0.249036
N	1.253165	0.683333	-0.421222
C	0.573478	-1.688038	0.186173
C	-1.113801	0.167615	-0.071581
H	2.012153	-1.100742	-1.220142
C	-2.191045	-0.084215	-1.142628
H	-1.726688	-0.345549	-2.102142
H	-2.841971	0.781296	-1.277704
C	-2.933092	-1.291983	-0.529708
H	-3.429473	-1.905939	-1.286525
H	-3.697640	-0.926111	0.162303
C	-1.850112	-2.094680	0.242466
H	-1.550348	-3.023324	-0.250431
H	-2.171892	-2.346102	1.258520
O	0.866626	-2.866283	0.370584
C	2.875020	-0.587352	0.684289
H	3.504311	-1.478806	0.659939
H	2.528693	-0.439234	1.713874
C	3.569199	0.679755	0.144362
H	4.231004	0.418900	-0.688871
H	4.177910	1.181043	0.901615
C	2.417146	1.588046	-0.356798
H	2.622178	2.027349	-1.338543
H	2.178681	2.407450	0.327408
H	-1.643392	0.631175	0.787415
O	-2.815088	2.192413	1.306661
H	-3.384482	2.357232	2.066146
O	-0.256557	2.357216	-0.537385
Li	-1.728980	3.072092	0.377665

E = -732.5308946 au (BMK/6-31+G(d) (H_2O)//B3LYP/6-31G(d))
This Molecule has 0 Imaginary Frequencies
Zero point vibrational energy: 159.55 kcal/mol

S3.2.8 c(D-Pro-D-Pro) + LiOH (Transition State)

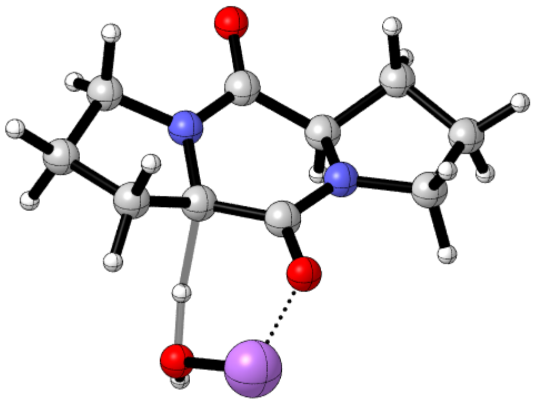

N	-0.814661	-1.163452	0.116556
C	-0.048436	1.099193	-0.428316
C	1.478647	-0.587425	0.563397
N	1.208151	0.631332	-0.209553
C	0.420147	-1.662985	0.323776
C	-1.146511	0.257558	-0.053612
C	-2.424368	0.237605	-0.898892
H	-2.199922	0.172148	-1.975203
H	-3.062676	1.113923	-0.748321
C	-3.112250	-1.055084	-0.419484
H	-3.844410	-1.441595	-1.135142
H	-3.634153	-0.865208	0.525731
C	-1.950975	-2.037860	-0.189532
H	-1.724121	-2.632484	-1.083758
H	-2.129374	-2.741526	0.630077
O	-0.209661	2.300636	-0.859868
O	0.692264	-2.865799	0.358133
H	-1.664118	1.217472	1.132555
O	-2.045496	2.165891	1.592077
H	-1.853247	2.124192	2.540733
Li	-1.279298	3.177808	0.280895
H	1.473237	-0.349305	1.645464
C	2.900181	-0.945582	0.127269
H	3.405409	-1.592850	0.847786
H	2.859682	-1.477989	-0.829047
C	2.432225	1.376475	-0.514583
H	2.445181	2.349056	-0.006220
H	2.483707	1.575754	-1.591180
C	3.564271	0.438770	-0.025963
H	4.412529	0.427281	-0.717008
H	3.941305	0.782720	0.944735

E = -732.4968815 au (BMK/6-31+G(d) (H_2O)//B3LYP/6-31G(d))
This Molecule has 1 Imaginary Frequency (i237 cm^{-1})
Zero point vibrational energy: 157.076 kcal/mol

S3.2.9 c(D-Pro-L-Pro) + LiOH (Transition State)

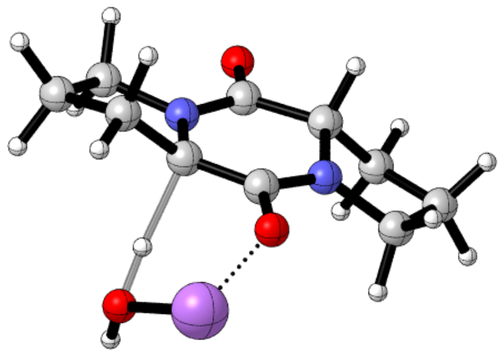

N	-0.833572	-1.108057	0.084061
C	0.021599	1.115945	-0.531841
C	1.548491	-0.861807	-0.350088
N	1.266738	0.577917	-0.416645
C	0.364822	-1.726194	0.083025
C	-1.100667	0.293412	-0.221499
H	1.838551	-1.234287	-1.348504
C	-2.405318	0.251882	-1.008834
H	-2.240181	-0.035554	-2.059883
H	-2.952964	1.198902	-1.006195
C	-3.174161	-0.863633	-0.265595
H	-3.939657	-1.339176	-0.887129
H	-3.672475	-0.436357	0.612220
C	-2.084984	-1.869605	0.181801
H	-2.014192	-2.746842	-0.470460
H	-2.245525	-2.238376	1.201672
O	-0.081627	2.381011	-0.762185
O	0.532894	-2.919050	0.347447
C	2.780108	-0.919348	0.565351
H	3.340440	-1.849253	0.446731
H	2.461680	-0.846102	1.612330
C	3.560915	0.335864	0.136735
H	4.147090	0.119241	-0.764189
H	4.258126	0.689926	0.901995
C	2.470910	1.384599	-0.183377
H	2.719093	1.997223	-1.057028
H	2.296521	2.073020	0.654808
H	-1.609248	1.232913	1.018299
O	-1.974531	2.118350	1.572394
H	-1.826535	1.948487	2.514740
Li	-1.099020	3.229343	0.421121

E = -732.4968815 au (BMK/6-31+G(d) (H_2O)//B3LYP/6-31G(d))
This Molecule has 1 Imaginary Frequency (i117 cm^{-1})
Zero point vibrational energy: 157.432 kcal/mol

S3.2 Analysis of Computational Results

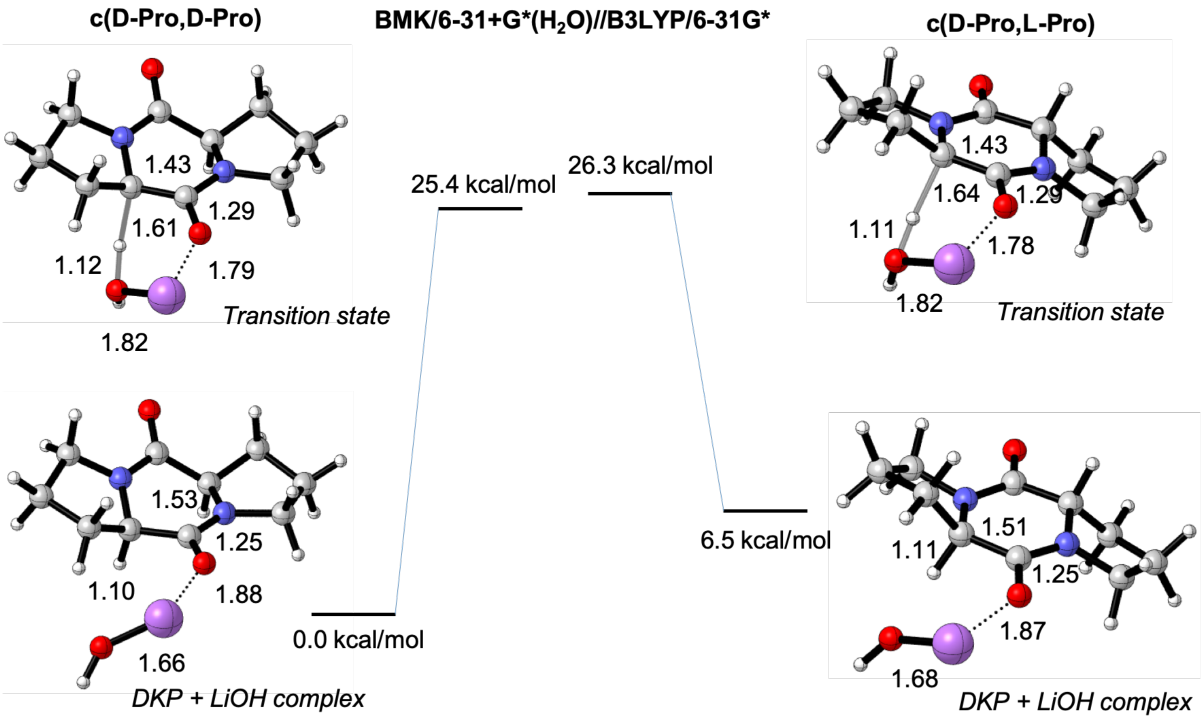

Figure S3.1: Free energy profile for the deprotonation of c(D-Pro-D-Pro) (left) and c(D-Pro-L-Pro) (right) by lithium hydroxide to form their respective enolates.

Figure S3.1 shows the free energy profile for the deprotonation of c(D-Pro-D-Pro) and c(D-Pro-L-Pro) by lithium hydroxide to form the enolates. The reactant state of c(D-Pro-D-Pro) is calculated to be more stable than c(D-Pro-L-Pro) by 6.5 kcal mol^{-1} due to the pyrrolidine rings in c(D-Pro-D-Pro) adopting the more stable envelope conformation while the pyrrolidine rings in c(D-Pro-L-Pro) are in the less stable half-chair conformation. The energy of both transition states are near equivalent (25.4 kcal mol^{-1} for c(D-Pro-D-Pro) and 26.3 kcal mol^{-1} for c(D-Pro-L-Pro)) which is expected as both compounds share a mutual enolate structure.

S4 References

1. P. E. Young, V. Madison and E. R. Blout, *J. Am. Chem. Soc.*, 1976, **98**, 5365-5371.
2. P. K. Glasoe and F. A. Long, *J. Phys. Chem.*, 1960, **64**, 188-190.
3. C. Eguchi and A. Kakuta, *J. Am. Chem. Soc.*, 1974, **96**, 3985-3989.
4. A. Rios, J. P. Richard and T. L. Amyes, *J Am Chem Soc*, 2002, **124**, 8251-8259.
5. L. G. Gagliardi, C. B. Castells, C. Ràfols, M. Rosés and E. Bosch, *Anal. Chem.*, 2007, **79**, 3180-3187.
6. E. Kiliç and N. Aslan, *Microchimica Acta*, 2005, **151**, 89-92.
7. R. S. Massey, C. J. Collett, A. G. Lindsay, A. D. Smith and A. C. O'Donoghue, *J. Am. Chem. Soc.*, 2012, **134**, 20421-20432.
8. C. J. T. de Grotthuss, *Ann. Chim.*, 1806, **58**, 54.
